# Developing and Validating a Patient‐Centered Measure of Well‐Being in Bipolar Disorder: The WELL‐B

**DOI:** 10.1111/bdi.70193

**Published:** 2026-10-24

**Authors:** Audrey R. Stromberg, Alexandra H. Vinson, Claudia Diaz‐Byrd, Ravleen Kaur, Melvin G. McInnis, Sarah H. Sperry

**Affiliations:** ^1^ Department of Psychiatry University of Michigan Ann Arbor Michigan USA; ^2^ Department of Learning Health Sciences University of Michigan Ann Arbor Michigan USA

**Keywords:** bipolar disorder, patient‐centered, psychometrics, thriving, validation, well‐being

## Abstract

**Objectives:**

Traditional measures of well‐being in bipolar disorder (BD) often equate well‐being with the absence of mood symptoms, overlooking strengths‐based indicators like acceptance, agency, and positive emotion that individuals with BD identify as central to their lived experience of wellness. To address this misalignment, we developed and psychometrically validated a new well‐being measure, the Well‐being Evaluation for Living with Bipolar (WELL‐B), grounded in the concept of thriving.

**Methods:**

The WELL‐B was co‐designed with individuals with BD and caregivers through a collaborative learning health system and piloted in the Prechter Longitudinal Study for Bipolar Disorder (PLS‐BD). Participants (*N* = 192) completed the WELL‐B twice over 2 months. The WELL‐B captured personalized, qualitative examples of well‐being across eight domains and corresponding quantitative ratings using four overarching prompts. Using both Classical Test Theory and Modern Test Theory frameworks, we evaluated factor structure, internal consistency, item‐level properties, test–retest reliability, and convergent and divergent validity.

**Results:**

Exploratory factor analysis revealed a stable three‐factor solution: general well‐being, time availability, and work‐related well‐being factors. All factors showed strong internal consistency (alphas > 0.85), and item response theory analyses indicated moderate to high item discrimination. The well‐being factor was strongly associated with better mental health functioning and lower depression, anxiety, and alcohol use. Most participants rated the WELL‐B as acceptable, feasible, and meaningful for reflecting on their wellness.

**Conclusions:**

The WELL‐B offers a strengths‐based, patient‐centered approach to assessing well‐being in BD. Future directions include refinement, broader validation, and testing clinical utility for treatment planning and monitoring.

## 1 Introduction

Affecting nearly 4% of the global population, bipolar disorder (BD) is a severe psychiatric condition defined by extreme shifts in mood, including episodes of (hypo)mania and depression [1]. Approximately 50% of individuals with BD report that BD significantly impairs their well‐being, disrupting the ability to work, maintain family and friend relationships, and carry out daily activities, regardless of the mood symptoms they experience [2, 3]. As improving well‐being is a central goal of research and clinical interventions in BD, it is essential to have accurate and meaningful assessments of well‐being. However, relatively little attention has been given to how well‐being is measured in research or clinical contexts.

Historically, well‐being in BD has been assessed using measures that capture the absence of mood symptoms, overlooking positive indicators like acceptance, resilience, and agency. For example, one popular well‐being measure is the WHO‐5 Well‐being Index, which consists of five items assessing the absence of negative symptoms as a proxy for well‐being (e.g., “I have felt calm and relaxed.”). Another popular measure of well‐being is the short‐form health survey (SF‐36) [4], where items ask about the absence of impairment (e.g., “Have you felt calm and peaceful?”). Inherent in these measures is the assumption that the absence of negative symptoms or impairments equates to well‐being. While practical, this translation may not hold true and may oversimplify the complex emotions and lived experiences of individuals with BD. For instance, someone might feel full of energy (as assessed by the SF‐36) yet still lack a sense of well‐being, highlighting that deficits‐based well‐being measures could be missing the mark.

Indeed, evidence has emerged suggesting there is a significant misalignment between how well‐being is traditionally measured in BD and how individuals with BD actually define their own well‐being. A national Depression and Bipolar Support Alliance (DBSA) survey of 3337 individuals with BD revealed that the absence of negative mood symptoms is not the most important aspect of their well‐being. Instead, respondents emphasized the importance of positive emotions (e.g., contentment), acceptance of symptoms, and agency in decision‐making in defining their well‐being [5]. These results echo the concept of human flourishing or thriving, which moves beyond symptom management alone. Positive psychology defines thriving as encompassing a complex and broad range of states and outcomes—including happiness and life satisfaction, meaning and purpose, character strengths, close social relationships, in addition to classic markers of mental and physical health [6]. Therefore, the findings from the DBSA survey highlight the need for well‐being measures that align with how individuals with BD conceptualize well‐being—that is, through the presence of thriving.

To address this misalignment, there is a critical need for a new well‐being measure that not only reflects the lived experiences of individuals with BDs, but also captures broader constructs of thriving. To accomplish this, rigorous psychometric validation is necessary to ensure the measure is both reliable and meaningful. Because well‐being is a latent construct, the survey must reliably assess observable aspects of well‐being that map onto this underlying construct. However, transitioning from raw item responses to a single score involves assumptions about how well the items reflect the latent characteristic of well‐being. Testing these assumptions is the goal of psychometric validation, which aims to ensure that the survey can make meaningful inferences about well‐being and therefore provide actionable insights for research and clinical practice.

To that end, the goal of this study was to psychometrically validate a newly developed well‐being measure for individuals with BD that was (1) co‐designed with and centered around the lived experience of individuals with BD and caregivers, and (2) tested and validated by individuals with BD and clinical psychologists, social workers, and medical doctors specializing in BD. This survey provides a patient‐centered framework for assessing well‐being in BD, emphasizing thriving over absence of symptoms. Our psychometric validation process included components from both Classical Test Theory (CTT), to examine test‐level properties like item‐scale correlations, and Modern Test Theory (MTT), to explore item‐level properties using methods such as item response theory and differential item functioning [7]. Lastly, we examined convergent and divergent validity with other mood and functioning measures administered and test re‐test reliability over 2 months. Given the profound impact of BD on well‐being and lack of adequate measurements thereof, the present study fills a critical gap within the BD research and clinical landscape.

## 2 Materials and Method

### 2.1 Survey Development

The process of survey creation, piloting, and testing was conducted in multiple stages involving different cohorts and settings. The survey was created and finalized through collaboration with the Bipolar Disorder Learning Community (BDLC) at the University of Michigan. The BDLC is a multi‐stakeholder group comprised of 15–20 individuals with lived experience of BD, family members, researchers, administrators, and clinicians at the University of Michigan [8]. At any given time, approximately 1/3 of BDLC members have lived experience or family experience of BD. Using a Learning Health System model, the members of the BDLC engage in co‐production of healthcare improvement efforts to enhance the well‐being of people living with BD. Co‐production is embedded into the BDLC at an organizational level; rather than engaging participants to provide feedback on ideas generated by researchers and clinicians, the BDLC as a multi‐stakeholder group generates ideas, scopes them to identify common ground across lived experience, clinical care, and research, and carries out initiatives as integrated multi‐stakeholder teams. Based on common ground articulated between members with lived experience and clinicians who identified shortcomings with conventional ways of assessing well‐being and thriving in BD care, the BDLC iteratively drafted a well‐being measure that emphasizes thriving and incorporates person‐centered benchmarks of well‐being. We used iterative round‐table discussions to build group consensus, using these discussions in place of cognitive pretesting to refine terms. This process involved synchronous review of existing well‐being measures, discussing their strengths and limitations, and analyzing the DBSA report on well‐being [5]. A recurring theme that emerged throughout this iterative process was the importance of enabling individuals with BD to recognize daily activities that typically enhance their well‐being and to reflect on their engagement in those activities when completing the measure. This insight informed the development of the two‐part questionnaire described below.

The Well‐being Evaluation for Living with Bipolar (WELL‐B) measure is comprised of two parts: qualitative open response (Part A) and quantitative ratings of well‐being (Part B). In Part A, participants are asked to provide self‐generated examples of what well‐being looks like for them across seven domains: (1) Achievement/Work/School, (2) Daily Responsibilities (e.g., chores), (3) Leisure (e.g., hobbies), (4) Personal (e.g., purpose in life, personal growth, pursuit of goals), (5) Relationships, (6) Physical Health, (7) Emotional (e.g., general mood, contentment, stability), and (8) Other. For each of these eight domains, participants are instructed to write at least one example (with the option to provide up to three) illustrating how they conceptualize and experience well‐being in that domain. The purpose of this qualitative, participant‐centered approach is to capture personalized and context‐specific understandings of well‐being for each individual across different life domains.

Next, in Part B, participants are asked to quantitatively evaluate their well‐being in each of the eight domains from Part A using four overarching prompts. These prompts are: (1) “Over the last month, I feel content with how I'm doing in the following areas:,” (2) “Over the last month, I have made progress on or had personal growth related to:,” (3) “Over the last month, I have had a sense of control over my:,” and (4) “Over the last month, I have had enough time to spend on:.” For each prompt, participants rate their experiences in each domain from Part A (e.g., Achievement/Work/School, Daily Responsibilities, Leisure, etc.) on a 5‐point Likert scale ranging from 1 (“Strongly Disagree”) to 5 (“Strongly Agree”). This results in 8 ratings per prompt for a total of 32 quantitative ratings per participant, allowing for a multidimensional and individualized understanding of well‐being over the past month.

The final Likert‐scale question on the survey asks participants to rate their agreement with the statement: “Over the last month, I have been living well.” Based on their response, branching logic directs them to one of two open‐ended questions. If they select 3 or lower (indicating disagreement or neutrality), they are asked, “What do you need to change to feel like your well‐being is at a higher level?” If they select 4 or 5 (indicating agreement), they are asked, “What are the biggest contributors to your well‐being?” The final version of the WELL‐B is provided in Supplemental Appendix 1.

### 2.2 Survey Validation

#### 2.2.1 Participants and Procedures

The WELL‐B was piloted in the Prechter Longitudinal Study for Bipolar Disorder (PLS‐BD) at the University of Michigan, an open cohort study initiated in 2005 which includes individuals with BD I, BD II, BD not otherwise specified (NOS), and healthy controls [9]. All participants with a BD diagnosis in the PLS‐BD were given the option to complete the WELL‐B at their regular bi‐monthly assessment point.

Those who consented (*N* = 276) were provided with both Parts A (qualitative write‐in responses) and B (quantitative ratings) of the WELL‐B at baseline. Of the 276 participants who consented, 192 participants completed both Parts A and B at baseline. Two months later, participants were asked to complete Part B again, this time providing quantitative ratings specifically about the domains they had written about in Part A during the baseline assessment. Of the 192 participants who completed the baseline assessment, 192 completed this 2‐month follow‐up. Following this second assessment, participants completed the satisfaction survey (Supplemental Appendix 1) to evaluate the acceptability and feasibility of the WELL‐B. Participants were provided compensation for participating. All research methods were approved by the Michigan Medicine Institutional Review Board.

#### 2.2.2 Measures

Convergent and divergent validity measures were drawn from the PLS‐BD bi‐monthly assessments. Participants completed the Patient Health Questionnaire (PHQ‐9). The PHQ‐9 [10] assesses depressive symptoms over the past 2 weeks with scores ranging from 0 (no depression) to 27 (severe depression). Mania was measured with the Altman Self‐Rating Mania Scale (ASRM). The five‐item ASRM [11] assesses hypomania/mania over the past week with scores ranging from 0 (no hypomania/mania) to 20 (severe hypomania/mania). The Generalized Anxiety Disorder Scale (GAD‐7) [12] assesses symptoms of generalized anxiety over the past two weeks. Scores range from 0 (no anxiety) to 21 (severe anxiety). The 12‐Item Short‐Form Health Survey (SF‐12) [13] assesses health‐related quality of life, capturing physical and mental health domains over the past 4 weeks. It generates two summary T scores: the Physical Component Summary (PCS) and the Mental Component Summary (MCS), each ranging from 0 to 100 (M = 50, SD = 10). Higher scores indicate better health‐related quality of life. The Alcohol Use Disorders Identification Test—Consumption (AUDIT‐C) [14] is a 3‐item questionnaire composed of the three items from the larger 10‐item AUDIT [15] which measure consumption of alcohol over the 2 months. Scores range from 0 (no alcohol use) to 12 (severe alcohol use). The Drug Use Disorders Identification Test—Consumption (DUDIT‐C) [16] is an 4‐item questionnaire composed of the first four consecutive consumption‐related questions from the larger 11‐item DUDIT, designed to measure drug use over the 2 months. Scores range from 0 (no drug use) to 16 (severe drug use). The short form version of the Cannabis Use Disorders Identification Test—Revised (CUDIT‐R) [17] is a 3‐item questionnaire that measures intensity and frequency of cannabis use over the last 2 months. Scores range from 0 (no cannabis use) to 12 (severe cannabis use).

A satisfaction survey was developed to assess the acceptability (e.g., response rate, completion time, clarity of instructions, ability to provide individualized examples) and feasibility (e.g., deployment via REDCap software) of the well‐being survey. A detailed description of all satisfaction survey questions and response options is provided in Supplement Appendix 1.

## 3 Results

### 3.1 Demographics

Of the 192 participants who completed the WELL‐B components, the majority (78%) were female. Approximately 73% of participants had BD I, 20% had BD II, and around 7% had BD NOS. The sample was predominantly White (85%), with 4.7% identifying as Black or African‐American, 4.7% as more than one race, 2.6% as Asian, 1.0% as Native American or Alaskan Native, and 1.6% as unknown or unreported. Additionally, most participants (94%) did not identify as Hispanic or Latino. Participants' ages at study entry ranged from 20 to 78 years old, with an average and median age of 49 years.

### 3.2 Descriptive Results

Quantitative ratings of well‐being—assessing participants' perceptions of having enough time for, a sense of control over, progress or personal growth on, and overall contentment with the well‐being domains—are presented in Figure 1. Across all well‐being domains, “agree” was the most commonly selected response, indicating generally positive perceptions. However, a notable exception emerged in the ‘other’ category, where “neutral” was the most frequent response, diverging from the pattern observed in other domains. When asked to rank their agreement with the statement, “Over the last month, I have been living well,” participants responded as follows: 16.4% selected “strongly agree,” 43.1% selected “agree,” 11.8% selected “neutral,” 17.4% selected “disagree,” and 10.8% selected “strongly disagree.”

**FIGURE 1:**
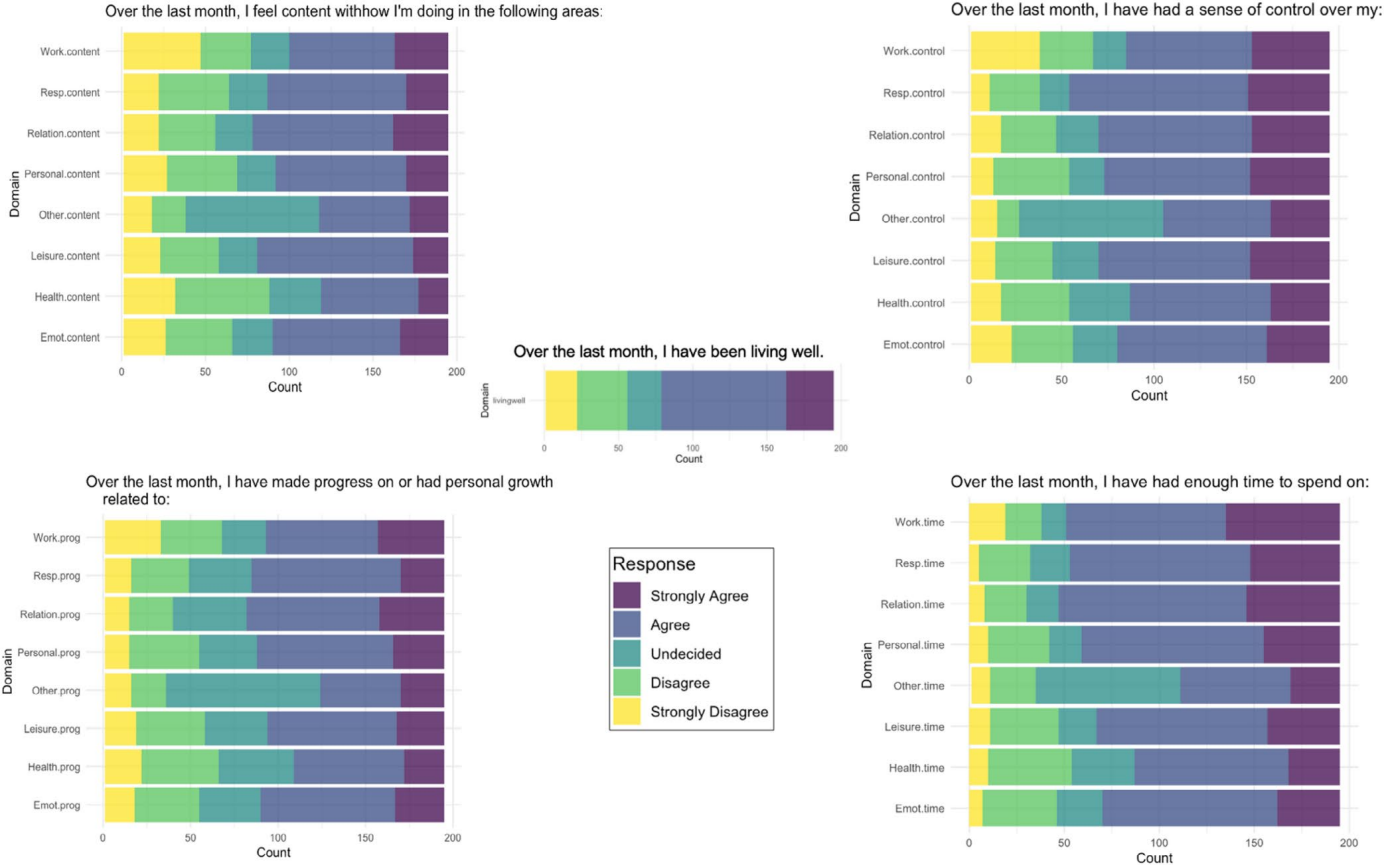
Quantitative ratings of well‐being. Counts are listed on the *x* axis with responses indicated in different colors according to the legend. Upper left reflects the feeling content around each of the activities identified across the WELL‐B domains. Upper right reflects having control over each of the activities identified across the WELL‐B domains. Bottom left reflects making progress on activities identified across the WELL‐B domains. Bottom right reflects having enough time to spend on activities identified across the WELL‐B domains.

### 3.3 Factor Structure

An exploratory factor analysis (EFA) was conducted using the *fa.parallel* function in R (Version 4.4.1). Parallel analysis suggested that the best‐fitting model consisted of six factors and three components. This was followed by a detailed EFA using Oblimin rotation for each of the six factor solutions. Fit indices (Chi Square, RMSEA, TLI, BIC, RMSR, Fit, and Fit off diagonal) for each potential solution were obtained. Next, item loadings were extracted for each item for each factor solution with a cutoff of 0.30. Items were flagged for cross‐loadings (an item has two or more loadings ≥ 0.30), ambiguity (the difference between the first and second strongest loadings was less than 0.20), and weak items (no loadings > = 0.30). All outputs are available in the Supplemental Appendix 2.

While model fit improved as additional factors were retained, all factor solutions showed imperfect fit based on RMSEA (all < 0.10) and TLI (all < 0.80). Given this, a final solution was selected prioritizing a model that (1) minimized the number of cross‐loaded, ambiguous, and weak items, (2) ensured an adequate number of items per factor, and (3) demonstrated the most theoretical interpretability. Although the six‐factor solution had the relative best fit based on fit indices, this solution produced 9 cross‐loaded items, 10 ambiguous items, and one weak item. The next best fits, five factor (12 cross‐loaded, 12 ambiguous, one weak) and 4 factor (5 cross‐loaded, 5 ambiguous), had similar problems. The three‐factor solution, which had the next best fit, only had one cross‐loaded item (time for work/school/achievement), no ambiguous items, and no weak items. The one cross‐loaded item showed no ambiguity with a clear preference toward one factor (0.71 vs. 0.36). Conceptually, the three‐factor solution produced factors that had conceptual coupling and theoretical interpretability, which factors 4 through 6 did not. As such, a three‐factor solution was selected as it minimized cross‐loaded items and offered best theoretical coherence (Table 1). The three factors were defined as follows:

- Well‐being factor: Includes most items, encompassing various aspects of well‐being and includes items focused on contentment, personal growth, and overall satisfaction.
- Time factor: Includes items assessing whether participants feel they have enough time to devote to the eight domains of well‐being.
- Work factor: Includes items specific to the achievement/work/school domain of well‐being.

**TABLE 1:** Exploratory factor analysis three‐factor solution loadings.

Item	Factor 1 well‐being	Factor 2 time	Factor 3 work
Responsibility.control	**0.64**	−0.12	0.24
Leisure.control	**0.59**	0.25	−0.02
Personal. control	**0.74**	0.16	−0.06
Relation.control	**0.61**	0.06	0.08
Health.control	**0.55**	0.06	0.10
Emot.control	**0.60**	0.04	0.22
Responsibility.progress	**0.49**	−0.09	0.25
Leisure.progress	**0.79**	0.05	−0.11
Personal.progress	**0.84**	0.01	−0.14
Relation.progress	**0.67**	0.01	0.03
Health.prog	**0.59**	0.03	−0.02
Emot.progress	**0.75**	−0.01	0.12
Responsibility.content	**0.71**	−0.09	0.21
Leisure.content	**0.80**	0.13	−0.14
Personal.content	**0.84**	0.10	−0.06
Relation.content	**0.69**	0.05	0.04
Health.content	**0.65**	0.01	0.00
Emot.content	**0.69**	0.05	0.15
livingwell	**0.72**	−0.10	0.17
Responsibility.time	0.00	**0.54**	0.24
Leisure.time	0.13	**0.75**	−0.06
Personal.time	0.11	**0.81**	−0.03
Relation.time	0.19	**0.55**	0.09
Health.time	0.00	**0.63**	−0.01
Emot.time	0.09	**0.61**	0.22
Work.time	−0.20	0.36	**0.71**
Work.control	0.12	0.03	**0.75**
Work.prog	0.19	−0.05	**0.65**
Work.content	0.30	−0.05	**0.71**

*Note:* Items are sorted by primary factor loading, and horizontal lines are used to denote groupings of items that loaded most strongly on each factor. Values are bolded in the column of the factor that they were assigned to.

The three factors showed small to large correlations with each other. The well‐being factor was correlated with time (*r* = 0.51) and work (*r* = 0.46). The time and work correlation was smaller (*r* = 0.21). Notably, items in the “Other” category had inconsistent quantitative ratings and feedback from the satisfaction survey indicated difficulty rating this category. Based on this feedback the other category was excluded from quantitative analyses in efforts to reduce item dimensions.

### 3.4 Psychometric Analysis

All psychometric analyses were performed on the three factors identified above. First, within the framework of CTT, item‐scale correlations were evaluated for each factor. For the well‐being factor, item‐scale correlations ranged from 0.59 to 0.94. The lowest correlations were observed for ratings of progress/personal growth in the domains of daily responsibilities (0.59) and physical health (0.64); however, these values are still considered adequate. For the time factor, item‐scale correlations ranged from 0.70 to 0.85, while for the work factor, correlations ranged from 0.77 to 0.88.

Internal consistency was assessed using Cronbach's alpha to evaluate how reliably the items within each factor measure their respective constructs. All factors demonstrated strong reliability, with Cronbach's alpha values of 0.95 for the well‐being factor, 0.87 for the time factor, and 0.86 for the work factor, indicating very good to excellent internal consistency.

Next, using the *mirt* package in R to conduct multidimensional item response theory (MIRT), we analyzed the relationship between individuals' responses to items within each factor and the underlying latent traits those items were designed to measure. Our focus was on the discrimination parameter *(a)*, which reflects how well an item distinguishes between individuals with varying levels of the latent trait. Discrimination values between 0.00 and 0.50 are considered low, 0.50 to 1.50 are moderate, and values above 1.50 indicate very high discrimination.

For the well‐being factor, discrimination parameters ranged from 1.18 to 3.07, indicating all items demonstrated moderate to high discrimination. Items with moderate discrimination (*a* < 1.50) included those assessing control over the physical health domain (1.41), progress/personal growth in the daily responsibilities domain (1.18), progress/personal growth in the physical health domain (1.26), and feeling content with the physical health domain (1.45). For the time factor, all items exhibited high discrimination, with values ranging from 1.76 to 3.66. Similarly, for the work factor, all items demonstrated high discrimination, with values ranging from 1.76 to 4.01.

Lastly, we conducted differential item functioning (DIF) analyses to examine whether individuals with different characteristics—specifically, gender identity—had different probabilities of selecting specific responses to an item, independent of their latent trait levels. To perform DIF, at least five responses are required in every combination of cells (i.e., response categories by group). Because fewer than five individuals endorsed a gender identity other than man or woman, our analysis was limited to participants identifying as either men or women. No items were flagged for DIF based on gender identity (man vs. woman).

### 3.5 Convergent and Divergent Validity

Participants completed the WELL‐B within two months of other assessments of mood, substance use, and mental and physical health functioning administered in the PLS‐BD. Zero‐order correlations between subscale scores for each factor (calculated as the average score of items within each factor) and established measures are presented in Figure 2.

**FIGURE 2:**
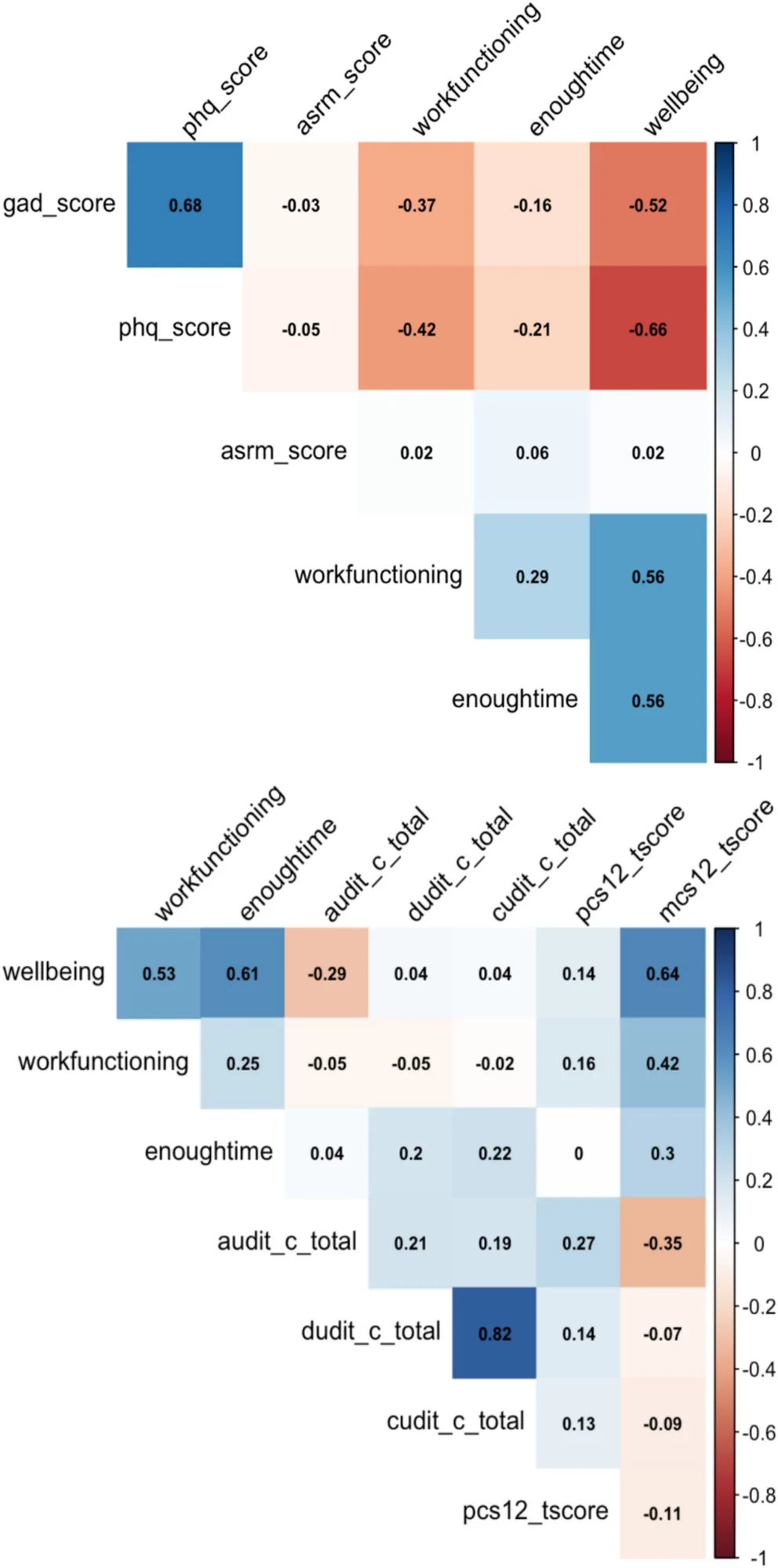
Correlations with three factors. Correlations in red are negative, correlations in blue are positive. The darkness of the color corresponds to the strength of the association (darker = stronger).

The associations between the three factors (well‐being, time, and work) and mood assessments (anxiety, depression, and mania symptoms) revealed different patterns of correlation. Scores on the well‐being factor demonstrated a strong negative correlation with anxiety and depression symptoms but no significant correlation with mania symptoms. This suggests that higher well‐being is associated with lower levels of anxiety and depression but is not related to mania symptoms. Scores on the time factor showed a small negative correlation with anxiety and depression symptoms and a very small but significant positive correlation with mania symptoms. This indicates that individuals who reported having enough time to spend on domains of well‐being experienced lower anxiety and depression symptoms but may also have slightly higher levels of manic symptoms. Finally, scores on the work factor showed a moderate negative correlation with anxiety and depression symptoms and no significant correlation with mania symptoms. This finding suggests that participants who felt content with, made progress on, and had a sense of control over the achievement/work/school domain of well‐being reported lower levels of anxiety and depression symptoms.

Substance use results (Figure 2) revealed that scores on the well‐being factor had a small negative association with alcohol use, suggesting that higher well‐being was linked to less alcohol use. However, well‐being factor scores were not significantly associated with cannabis or other drug use. Scores on the time factor demonstrated a small positive association with cannabis and drug use, indicating that individuals who felt they had enough time to dedicate to well‐being domains might also engage in slightly higher cannabis or drug use. In contrast, scores on the work subscale were not significantly associated with any substance use measures.

Functioning outcomes (Figure 2) showed that scores on the well‐being factor were positively correlated with both physical and mental health functioning, demonstrating a small positive association with physical health and a large positive association with mental health functioning. This suggests that individuals reporting high well‐being also reported better overall functioning across these domains. Scores on the time factor were unrelated to physical health functioning but had a moderate positive association with mental health functioning, suggesting a connection between perceived time availability and better mental health. Finally, scores on the work factor demonstrated a small positive association with physical health functioning and a moderate positive association with mental health functioning, indicating that satisfaction and progress in the achievement/work/school domain were linked to better overall functioning.

Next, linear regression models were conducted to examine the predictive utility of the three factors for mood, substance use, and physical and mental health functioning measures, resulting in a total of eight models. Results for mood measures are summarized in Table 2. When all three factors were included as predictors, higher well‐being factor scores were strongly associated with lower depression and anxiety symptoms (large effects), while no association was observed with mania symptoms. Interestingly, higher scores on the time factor were associated with higher depression symptoms (small effect), suggesting there may be a suppression effect caused by multicollinearity among the three factors. Similarly, scores on the work factor were not significantly associated with mood symptoms in these models, further supporting the possibility that shared variance between the three subscales might suppress individual effects. Due to this, we examined Variance Inflation Factors (VIF) for each of our models. VIFs ranged from 1.45 to 1.92, which suggests low multicollinearity.

**TABLE 2:** Three well‐being factors and depression, mania, and anxiety symptoms.

Predictors	PHQ‐9					ASRM					GAD‐7				
	Estimates	Std. beta	CI	Standardized CI	*p*	Estimates	Std. beta	CI	Standardized CI	*p*	Estimates	Std. beta	CI	Standardized CI	*p*
Intercept	21.00	0.00	16.66–25.34	−0.14 to 0.14	**< 0.001**	1.09	0.00	−1.53 to 3.70	−0.19 to 0.19	0.411	17.23	0.00	12.51–21.94	−0.16 to 0.16	**< 0.001**
Well‐being	−5.41	−0.75	−6.83 to −4.00	−0.94 to −0.55	**< 0.001**	−0.09	−0.03	−0.95 to 0.76	−0.30 to 0.24	0.832	−3.88	−0.57	−5.42 to −2.34	−0.79 to −0.34	**< 0.001**
Work	−0.42	−0.07	−1.39 to 0.55	−0.24 to 0.10	0.389	0.03	0.01	−0.55 to 0.62	−0.22 to 0.25	0.906	−0.63	−0.12	−1.68 to 0.43	−0.31 to 0.08	0.240
Time	1.64	0.23	0.41–2.88	0.06–0.40	**0.010**	0.24	0.07	−0.51 to 0.98	−0.16 to 0.31	0.530	1.30	0.19	−0.05 to 2.64	−0.01 to 0.38	0.059
Observations	107					107					107				
*R* ^2^/*R* ^2^ adjusted	0.478/0.463					0.004/−0.025					0.310/0.290				

*Note: p* values < 0.05 are bolded. Cis represent 95% confidence intervals.

Abbreviation: std. = standardized.

Results for the regressions related to substance use are presented in Table 3. Higher well‐being factor scores were strongly associated with fewer problems related to alcohol use (large effect). Conversely, higher scores on the time factor were moderately associated with greater problems related to both alcohol and cannabis use. Other potential associations were likely suppressed by the inclusion of all three factor scores as predictors in the same model. VIFs ranged from 1.41 to 2.12, which suggests low multicollinearity for work functioning and time and moderate (but acceptable) for the well‐being factor.

**TABLE 3:** Three factors and alcohol, drug, and cannabis consumption.

Predictors	AUDIT‐C					DUDIT‐C					CUDIT‐C				
	Estimates	Std. beta	CI	Standardized CI	*p*	Estimates	Std. beta	CI	Standardized CI	*p*	Estimates	Std. beta	CI	Standardized CI	*p*
Intercept	2.17	−0.00	0.22–4.12	−0.19 to 0.19	**0.030**	−0.46	0.00	−2.51 to 1.60	−0.21 to 0.21	0.661	−0.57	0.00	−2.90 to 1.76	−0.21 to 0.21	0.627
Well‐being	−1.50	−0.62	−2.18 to −0.81	−0.90 to −0.33	**< 0.001**	−0.18	−0.08	−0.91 to 0.54	−0.38 to 0.23	0.617	−0.40	−0.15	−1.22 to 0.42	−0.45 to 0.16	0.333
Work	0.34	0.18	−0.09 to 0.77	−0.05 to 0.41	0.118	−0.14	−0.07	−0.59 to 0.32	−0.32 to 0.17	0.554	−0.04	−0.02	−0.55 to 0.47	−0.27 to 0.23	0.880
Time	0.88	0.37	0.29–1.47	0.12–0.62	**0.004**	0.61	0.26	−0.02 to 1.23	−0.01 to 0.53	0.056	0.85	0.32	0.14–1.55	0.05–0.59	**0.019**
Observations	89					89					89				
*R* ^2^/*R* ^2^ adjusted	0.185/0.157					0.051/0.018					0.066/0.034				

*Note: p* values < 0.05 are bolded. Cis represent 95% confidence intervals. Note that the number of observations is lower as the AUDIT‐C, DUDIT‐C, and CUDIT‐C began being administered after the initiation of the well‐being survey.

Abbreviation: std. = standardized.

For physical and mental health functioning (Table 4), higher well‐being factor scores were significantly associated with better mental health functioning (large effect). Neither the time factor nor the work factor showed significant associations with physical or mental health functioning, again highlighting potential suppression effects when including all three factor scores as concurrent predictors in the model.

**TABLE 4:** Three factors and mental and physical well‐being.

Predictors	MCS‐12					PCS‐12				
	Estimates	Std. beta	CI	Standardized CI	*p*	Estimates	Std. beta	CI	Standardized CI	*p*
Intercept	13.33	0.00	4.04–22.62	−0.16 to 0.16	**0.005**	40.47	0.00	27.92–53.02	−0.21 to 0.21	**< 0.001**
Well‐being	9.26	0.67	5.99–12.52	0.43–0.91	**< 0.001**	2.13	0.15	−2.28 to 6.54	−0.16 to 0.46	0.341
Work	1.08	0.10	−0.97 to 3.12	−0.09 to 0.29	0.298	1.21	0.11	−1.55 to 3.98	−0.14 to 0.36	0.385
Time	−1.81	−0.13	−4.62 to 1.00	−0.34 to 0.07	0.204	−1.64	−0.12	−5.43 to 2.16	−0.39 to 0.15	0.394
Observations	89					89				
*R* ^2^/*R* ^2^ adjusted	0.433/0.413					0.038/0.004				

*Note: p* values < 0.05 are bolded. Cis represent 95% confidence intervals.

Abbreviations: MCS‐12 = mental component summary; PCS‐12 = physical component summary; std = standardized.

### 3.6 Test–Retest Reliability

Given that the goal is to create a measure that can capture fluctuations (both improvements and diminutions in well‐being) we re‐administered the measure approximately 2 months after the initial administration, with a mean interval of 86 days (range: 60–167 days). Intraclass correlation coefficient (ICC) values were calculated to assess the proportion of total variance attributable to within‐ versus between‐person differences across repeated measures. Within‐person ICC values between 0.7 and 0.8 are considered high, with values above 0.8 indicating excellent consistency. Between‐person ICC values between 0.4 and 0.6 are considered moderate to high, with values above 0.6 indicating high consistency.

For the well‐being factor, the within‐person ICC was 0.58, and the between‐person ICC was 0.42. For the time factor, the within‐person ICC was 0.48, and the between‐person ICC was 0.52. For the work factor, the within‐person ICC was 0.57, and the between‐person ICC was 0.43. Consistent with our expectations and goals, these values suggest considerable variability in scores on the well‐being measure over a 2‐month span, indicating that it captures fluctuations in functioning rather than reflecting a static or trait‐based construct.

### 3.7 Satisfaction Survey

Participants completed a satisfaction survey to evaluate their experience with the WELL‐B. When asked if they would take the WELL‐B again, 91.1% (153 participants) responded affirmatively, 6.5% (11 participants) were undecided, and 2.4% (4 participants) indicated they would not take it again. Regarding the ideal frequency of completing the measure, 71% (119 participants) preferred taking it every 2 months, 26.3% (44 participants) suggested every 6 months, and 3% (5 participants) indicated once per year.

Participants also reported how easy or difficult they found it to rate various aspects of well‐being. The percentages of responses across categories are summarized below:

- Time spent in each wellness category: 64.2% rated it “easy” or “very easy,” 25.6% were “neutral,” and 10.2% rated it “difficult” or “very difficult.”
- Progress made in each wellness category: 63.5% rated it “easy” or “very easy,” 24.6% were “neutral,” and 11.9% rated it “difficult” or “very difficult.”
- Control over each wellness category: 67.2% rated it “easy” or “very easy,” 31.3% were “neutral,” and 1.5% rated it “difficult” or “very difficult.”
- Contentment with performance in each wellness category: 74.6% rated it “easy” or “very easy,” 23.4% were “neutral,” and 2% rated it “difficult” or “very difficult.”

The satisfaction survey also assessed the perceived impact of completing the measure on participants' wellness attitudes and behaviors:

- Impact on thinking about personal wellness: 80.3% responded affirmatively (“agree” or “strongly agree”), 18.1% were neutral, and 1.6% responded negatively (“disagree” or “strongly disagree”).
- Impact on wellness behaviors: 45.1% responded affirmatively, 45.2% were neutral, and 9.7% responded negatively.
- Impact on refining wellness goals: 59.7% responded affirmatively, 34.5% were neutral, and 5.8% responded negatively.

These findings suggest that most participants found the WELL‐B user‐friendly and impactful, particularly in influencing how they think about wellness. Fewer participants reported an effect on their behaviors or wellness goal setting.

## 4 Discussion

The goal of this study was to develop and psychometrically validate a novel well‐being measure for individuals with BD, co‐designed with and centered around the lived experiences of individuals with BD and their caregivers. As a collaborative learning health system, the BDLC practices co‐production of healthcare improvement initiatives in order to identify and address priorities that are relevant to clinicians, researchers, and people with lived experience and their loved ones [8, 18, 19]. Fostering an ethos of co‐production in the BDLC allowed individuals with lived experience to express dissatisfaction with current approaches to measuring wellness and to participate in developing a new way forward, resulting in a measure of well‐being that is strengths‐based and individually tailored to the life activities that a person with BD may already use to benchmark their well‐being. The WELL‐B was also inspired by findings from the DBSA survey [5], which emphasized thriving over the mere absence of symptoms. Using CTT and MTT frameworks, we evaluated test‐ and item‐level properties, as well as convergent and divergent validity and test–retest reliability. Together, these results indicated that the WELL‐B is a reliable and valid tool for assessing individualized aspects of well‐being in BD.

Our EFA identified a three‐factor solution with strong theoretical interpretability. The well‐being factor emerged as the most robust, encompassing the majority of items and reflecting core aspects of well‐being, including contentment, personal growth, and life satisfaction. The time factor captured perceptions of having enough time to dedicate to well‐being domains, while the work factor represented achievement‐related aspects, such as work, school, or goal progress.

The well‐being measure demonstrated strong construct validity through convergent and divergent validity analyses. Scores on the well‐being factor were positively associated with mental and physical health functioning and negatively associated with depression, anxiety, and alcohol use, highlighting its alignment with related constructs. The time factor showed small positive associations with manic symptoms and substance use, suggesting that perceptions of time availability may have nuanced relationships with specific outcomes. The work factor, in contrast, was primarily linked to reduced anxiety and depression symptoms. Of note, the small or non‐significant associations with manic symptoms across factors may reflect the low incidence of increased manic symptoms during the study period rather than indicating a lack of relationship between well‐being and mania. Overall, these findings underscore the measure's ability to capture meaningful aspects of well‐being that are distinct yet interconnected.

Regarding reliability, the well‐being measure demonstrated strong internal consistency, with Cronbach's alpha values exceeding 0.85 for all factors (0.86, 0.87, 0.95). These findings indicate good to excellent reliability and suggest that the items within each factor are coherent. However, the alpha of **0.95** may also indicate potential item redundancy. Future work should examine whether the well‐being factor could be shortened without compromising reliability, validity, or construct coverage. Item‐scale correlations were generally high, with moderate correlations observed for a few items within the well‐being factor, such as progress on and control over daily responsibilities and physical health domains. Additionally, MIRT analyses revealed moderate to high discrimination across factors, supporting the measure's ability to differentiate between individuals with varying levels of the latent traits assessed. Test–retest reliability analyses revealed moderate consistency over a 2‐month period. These findings suggest that the measure captures meaningful fluctuations in well‐being over time, particularly for the well‐being factor, which exhibited greater variability compared to the time and work factors.

Together, these findings indicate that the WELL‐B is a strong measure of well‐being for individuals with BD and the measure demonstrates solid psychometric properties that align with established standards for reliability and validity. Beyond its psychometric performance, this measure has important implications for improving how well‐being is assessed in BD. Traditional approaches often focus on the absence of symptoms or impairments, potentially missing important aspects of thriving. By centering the lived experiences of individuals with BD and incorporating multidimensional domains, this measure shifts the focus toward promoting positive well‐being and thriving. Clinicians and researchers could use this tool to identify specific areas for intervention and monitor progress over time.

Despite these strengths, several limitations warrant consideration. Notably, the observed suppression effects in regression analyses suggest multicollinearity among the three factors. While this is intuitive given the conceptual overlap between the factors (e.g., well‐being as a broad construct encompassing time and work‐related domains), it highlights a need to refine the measure further. Of note, VIF values indicated that there were low to moderate levels of multicollinearity both of which are considered acceptable. One potential future direction could involve simplifying the measure by focusing exclusively on the well‐being factor, which accounted for the majority of the variance and showed the strongest associations with key outcomes. This would also potentially benefit participants by reducing the amount of ratings needed on the measure, which some may find burdensome (although, note that survey length was not brought up as a concern on the Satisfaction Survey). Next, our sample size of 192 participants was smaller than ideal for the planned statistical analyses, which typically require at least 500 participants to ensure robust factor solutions and generalizability. While our results were promising, future research should replicate these analyses with larger and more diverse samples to confirm the measure's psychometric properties and improve its generalizability to underrepresented groups, including individuals from non‐White racial/ethnic backgrounds and those with BD NOS. Third, while the measure captures fluctuations in well‐being over time, the 2‐month test–retest interval may not fully capture long‐term changes or stability. Future studies could explore shorter or longer follow‐up periods to better understand how well‐being evolves and whether the measure remains sensitive to changes in response to interventions. Finally, while the satisfaction survey indicated high acceptability and ease of use, a minority of participants reported difficulty rating their control over and contentment with well‐being domains. Additional qualitative research could help refine these items to ensure clarity and relevance. Of note, a larger study is currently underway which administered the WELL‐B to a larger more diverse sample which will aid in confirming the factor structure of the measure and assessing the feasibility of just measuring the well‐being factor and conducting item refinement and item reduction.

This study offers initial validation for a novel well‐being measure that aligns with the lived experiences and priorities of individuals with BD. Its strong psychometric properties, patient‐centered design, and multidimensional focus make the WELL‐B a valuable tool for advancing research and clinical care. Future efforts should refine the WELL‐B, expand validation to larger and more diverse populations, and explore its role in treatment settings. Ultimately, this measure has the potential to illuminate factors that promote thriving and resilience in BD, contributing to personalized, strengths‐based care.

## Author Contributions

**Audrey R. Stromberg:** conceptualization, methodology, formal analysis, investigation, writing – original draft, visualization. **Alexandra H. Vinson:** conceptualization, methodology, writing – original draft, supervision. **Claudia Diaz‐Byrd:** conceptualization, project administration, writing – review and editing. **Ravleen Kaur:** data curation, formal analysis, writing – review and editing. **Melvin G. McInnis:** funding acquisition, supervision, writing – review and editing. **Sarah H. Sperry:** conceptualization, methodology, formal analysis, investigation, writing‐original draft, validation, supervision, funding acquisition.

## Funding

This work was supported by the National Institute of Mental Health, Prechter Family Charitable Fund, and Richard Tam Foundation.

## Conflicts of Interest

S.H.S. has grant funded support from a Brain and Behavior Foundation Young Investigator Award (#30719), a National Institute of Mental Health Loan Repayment Award (L30MH127613), and National Institute of Mental Health K23 Career Development Award (K23MH131601). S.H.S. is a consultant for Boehringer‐Ingelheim International gmbh with no direct relation to the current project. M.G.M. has support for the present manuscript from the Prechter Family Charitable Fund, the Richard Tam Foundation, and the National Institute of Mental Health (R01MH130411). M.G.M. also discloses a grant from Janssen Pharmaceuticals and two US Patents (#9,685,174; #11,545,173). The other authors declare no conflicts of interest.

## Supporting information


**Supplemental Appendix 1** WELL‐B—Well‐being Evaluation for Living with Bipolar.

**Supplemental Appendix 2** Supporting Information.

## Data Availability

The data that support the findings of this study are available on request from the corresponding author. The data are not publicly available due to privacy or ethical restrictions.
