# Supplementary material for: Developing and Validating a Patient‐Centered Measure of Well‐Being in Bipolar Disorder: The WELL‐B
Source: Bipolar Disord. 2026 Oct 24;28(7):e70193. doi: 10.1111/bdi.70193 (PMC13695044; doi:10.1111/bdi.70193)
Supplement: Supplementary file 1 [file BDI-28-0-s002.docx]

Supplemental Appendix 1

**Developing and Validating a Patient-Centered Measure of Well-Being in Bipolar Disorder**

Audrey R. Stromberg, M.S.,^1^ Alexandra H. Vinson, Ph.D.,^1,2^ Claudia Diaz-Byrd, M.S.,^1,2^ Ravleen Kaur, M.S.,^1^ Melvin G. McInnis, M.D.,^1^ Sarah H. Sperry, Ph.D.^1^

^1^Department of Psychiatry, University of Michigan, Ann Arbor, MI, USA

^2^Department of Learning Health Sciences, University of Michigan, Ann Arbor, MI, USA

WELL-B Measure

Satisfaction Survey

**WELL-B** – **Well-being Evaluation for Living with Bipolar**

We are interested in learning about your well-being and what you do to live well across numerous areas in your life. Please give examples of **what it looks like for YOU to be living well.** Now, think about times when you felt well. What types of things were you doing when you were feeling well? There are 8 categories of wellness below. If you can't think of an example for a certain category or three examples within a category, that is okay. Provide all the examples you can in each of the categories below.

**Here is an example of how to fill out this form:**

***Example:***

| Category | Example 1 | Example 2 | Example 3 |
| --- | --- | --- | --- |
| A. Achievement/work/school   (e.g., Meeting goals for work or school) | Not missing work | Meeting deadlines at work | Feeling like I’m achieving things |
| B. Daily responsibilities   (e.g., Chores, taking care of pets) | Doing laundry at least once a week | Walking the dog three times a day |  |
| C. Leisure   (e.g., Hobbies, crafts, private or social leisure activities) | Having time to do my pottery | Sometimes relaxing and watching my favorite TV |  |
| D. Personal   (e.g., Purpose in life, personal growth, setting and reaching goals, self-acceptance, follow through on ideas and intentions) | Learning a new skill in pottery |  |  |
| E. Relationships   (e.g., Positive and supportive relations with others) | Spending time with friends at least twice per month | Calling my best friend weekly |  |
| F. Physical Health   (e.g., general, self-care, fitness/activity, diet, sleep, energy, chronic medical conditions, taking medications) | Taking a walk 5 days per week | Taking my medications daily |  |
| G. Emotional   (e.g., General mood, contentment, stability, BD symptoms) | Feeling strong | Feeling calm | Feeling at peace |
| H. Other   (e.g., Spirituality/religion) | Going to church every Sunday |  |  |

Now, please give examples of ***what it looks like for you to be living well****.* Remember, if you can’t think of an example for a certain category or three examples within a category, that is okay. Provide examples in the categories that you can!

| Category | Example 1 | Example 2 | Example 3 |
| --- | --- | --- | --- |
| A. Achievement/work/school   (e.g., Meeting goals for work or school) |  |  |  |
| B. Daily responsibilities   (e.g., Chores, taking care of pets) |  |  |  |
| C. Leisure   (e.g., Hobbies, crafts, private or social leisure activities) |  |  |  |
| D. Personal   (e.g., Purpose in life, personal growth, setting and reaching goals, self-acceptance, follow through on ideas and intentions) |  |  |  |
| E. Relationships   (e.g., Positive and supportive relations with others) |  |  |  |
| F. Physical Health   (e.g., general, self-care, fitness/activity, diet, sleep, energy, chronic medical conditions) |  |  |  |
| G. Emotional   (e.g., General mood, contentment, stability, BD symptoms) |  |  |  |
| H. Other   (e.g., Spirituality/religion) |  |  |  |

Next, we want to know information about how well you’ve been doing in each of the categories listed above. Rate the extent to which you agree or disagree with the following questions **over the last month**:

|  | I have had enough time to spend on: | | | | |
| --- | --- | --- | --- | --- | --- |
|  | Strongly Disagree (1) | Disagree (2) | Undecided (3) | Agree (4) | Strongly Agree (5) |
| Achievement/Work/School |  |  |  |  |  |
| Daily Responsibilities |  |  |  |  |  |
| Leisure |  |  |  |  |  |
| Personal |  |  |  |  |  |
| Relationships |  |  |  |  |  |
| Physical Health |  |  |  |  |  |
| Emotional |  |  |  |  |  |
| Other |  |  |  |  |  |

|  | I have had a sense of control over my: | | | | |
| --- | --- | --- | --- | --- | --- |
|  | Strongly Disagree (1) | Disagree (2) | Undecided (3) | Agree (4) | Strongly Agree (5) |
| Achievement/Work/School |  |  |  |  |  |
| Daily Responsibilities |  |  |  |  |  |
| Leisure |  |  |  |  |  |
| Personal |  |  |  |  |  |
| Relationships |  |  |  |  |  |
| Physical Health |  |  |  |  |  |
| Emotional |  |  |  |  |  |
| Other |  |  |  |  |  |

|  | I have made progress on or had personal growth related to: | | | | |
| --- | --- | --- | --- | --- | --- |
|  | Strongly Disagree (1) | Disagree (2) | Undecided (3) | Agree (4) | Strongly Agree (5) |
| Achievement/Work/School |  |  |  |  |  |
| Daily Responsibilities |  |  |  |  |  |
| Leisure |  |  |  |  |  |
| Personal |  |  |  |  |  |
| Relationships |  |  |  |  |  |
| Physical Health |  |  |  |  |  |
| Emotional |  |  |  |  |  |
| Other |  |  |  |  |  |

|  | I feel content with how I’m doing in the following areas: | | | | |
| --- | --- | --- | --- | --- | --- |
|  | Strongly Disagree (1) | Disagree (2) | Undecided (3) | Agree (4) | Strongly Agree (5) |
| Achievement/Work/School |  |  |  |  |  |
| Daily Responsibilities |  |  |  |  |  |
| Leisure |  |  |  |  |  |
| Personal |  |  |  |  |  |
| Relationships |  |  |  |  |  |
| Physical Health |  |  |  |  |  |
| Emotional |  |  |  |  |  |
| Other |  |  |  |  |  |

5. Over the last month, I have been living well:

| Strongly Disagree (1) | Disagree (2) | Undecided (3) | Agree (4) | Strongly Agree (5) |
| --- | --- | --- | --- | --- |

5.1 (*Logic: If strongly disagree, disagree, undecided)*: What do you need to change to feel like your well-being is at a higher level? *Free Text*

5.2: (*Logic: If Agree or Strongly Agree*) What are the things you are doing that are the biggest contributors to your well-being? *Free Text*

**WELL-B: Satisfaction Survey**

Thank you for taking the wellness survey! Your responses will help us understand the relationship between everyday activities and wellbeing for people living with bipolar disorder.

To complete your participation, please fill out these questions. We will use your responses to improve this wellness survey so that it is as useful and easy to fill out as possible.

1. Would you be willing to take this survey on an ongoing basis as part of the longitudinal study?
   i. Yes/No/Undecided
   b. If yes: How often would you be willing to complete it?
      i. every 2 months, every 6 months, once per year
2. How easy or difficult did you find it to think of examples for each wellness category?
   i. very difficult - difficult - neither difficult nor easy - easy - very easy
3. How easy or difficult did you find it to:
   a. Rate your **time spent** for each wellness category
      i. very difficult - difficult - neutral - easy - very easy
   b. Rate your **progress made** for each wellness category
      i. very difficult - difficult - neutral - easy - very easy
   c. Rate your **control over** each wellness category
      i. very difficult - difficult - neutral - easy - very easy
   d. Rate your **contentment** with how you are doing in each wellness category
      i. very difficult - difficult - neutral - easy - very easy
4. How much do you agree or disagree with the following statements:
a. Completing this survey impacted the way I think about my personal wellness
   i. Strongly disagree, disagree, neutral, agree, Strongly agree
b. Completing this survey impacted my wellness behaviors
   i. Strongly disagree, disagree, neutral, agree, Strongly agree
c. Completing this survey has helped me refine my wellness goals
   i. Strongly disagree, disagree, neutral, agree, Strongly agree
5. What did you like or dislike about this survey? (Open response)
6. Is there anything else you would like us to know about your experience taking this survey? (Open response)
