# Supplementary material for: Developing and Validating a Patient‐Centered Measure of Well‐Being in Bipolar Disorder: The WELL‐B
Source: Bipolar Disord. 2026 Oct 24;28(7):e70193. doi: 10.1111/bdi.70193 (PMC13695044; doi:10.1111/bdi.70193)
Supplement: Supplementary file 2 [file BDI-28-0-s001.html]

Developing and Validating a Patient-Centered Measure of Well-Being in Bipolar Disorder: The WELL-B


# Developing and Validating a Patient-Centered Measure of Well-Being in Bipolar Disorder: The WELL-B

#### Sarah Sperry

#### 2026-08-05

## 1 Load relevant packages

Load in packages

```
library(mirt)
library(lavaan)
#library(semPlot)
library(OpenMx)
library(tidyverse)
#library(GGally)
library(foreign)
library(tidyverse)
library(ggplot2)
library(data.table)
library(Hmisc)
library(MASS)
library(optimx)
library(lme4)
library(lmerTest)
#library(texreg)
library(xtable)
library(lattice)
library(stringi)
#library(HLMdiag)
library(sjPlot)
library(table1)
library(dplyr)
library(readr)
```

## 2 Read in data

Initial number in baseline is 276. But we have some people with NA’s
on the quantitative part (n = 81). So final sample size for quantitative
part 1 is 195.

```
WB.df <- read.csv("/Users/sperrys/University of Michigan Dropbox/Sarah Sperry/EmoTe Lab/Audrey Stromberg Research/Well-being Project/Data/WellbeingStudy_DATA_2024-07-02_1046.csv")

## Baseline assessment

WB.baseline <- WB.df %>%
  filter(redcap_event_name == "baseline_arm_1" & online_consent_complete == 2 & !is.na(wb_1_1))

## Make a shorter file with variables that we are including 

WB.base.filter <- WB.baseline[c(1,2,36:69,72)]

## rename variables to be more usable

WB.base.filt.V2 <- WB.base.filter %>%
  rename(Work.time = wb_1_1, 
         Resp.time = wb_2_1, 
         Leisure.time = wb_3_1, 
         Personal.time = wb_4_1, 
         Relation.time = wb_5_1, 
         Health.time = wb_6_1, 
         Emot.time = wb_7_1, 
         Other.time = wb_8_1, 
         Work.control = wb_1_2, 
         Resp.control = wb_2_2, 
         Leisure.control = wb_3_2, 
         Personal.control = wb_4_2, 
         Relation.control = wb_5_2, 
         Health.control = wb_6_2, 
         Emot.control = wb_7_2, 
         Other.control = wb_8_2,
         Work.prog = wb_1_3, 
         Resp.prog = wb_2_3, 
         Leisure.prog = wb_3_3, 
         Personal.prog = wb_4_3, 
         Relation.prog = wb_5_3, 
         Health.prog = wb_6_3, 
         Emot.prog = wb_7_3, 
         Other.prog = wb_8_3, 
         Work.content = wb_1_4, 
         Resp.content = wb_2_4, 
         Leisure.content = wb_3_4, 
         Personal.content = wb_4_4, 
         Relation.content = wb_5_4, 
         Health.content = wb_6_4, 
         Emot.content = wb_7_4, 
         Other.content = wb_8_4,
         livingwell = wb_5)

WB.base.filt.V2$date <- as.POSIXct(WB.base.filt.V2$wb_part_b_timestamp, format = "%m/%d/%y")

## Make these variables ordinal 

variable_names <- c("Work.time", "Resp.time", "Leisure.time", "Personal.time", "Relation.time",
                    "Health.time", "Emot.time", "Other.time", "Work.control",
                    "Resp.control", "Leisure.control", "Personal.control", "Relation.control",
                    "Health.control", "Emot.control", "Other.control", "Work.prog",
                    "Resp.prog", "Leisure.prog", "Personal.prog", "Relation.prog",
                    "Health.prog", "Emot.prog", "Other.prog", "Work.content",
                    "Resp.content", "Leisure.content", "Personal.content", "Relation.content",
                    "Health.content", "Emot.content", "Other.content", "livingwell")

# Convert each variable to factor
WB.base.filt.V2[variable_names] <- lapply(WB.base.filt.V2[variable_names], factor)
```

## 3 Baseline

### 3.1 Summary Statistics

```
## Quant numbers
table1::table1(~ Work.time + Resp.time + Leisure.time + Personal.time + Relation.time +
Health.time + Emot.time + Other.time + Work.control +
Resp.control + Leisure.control + Personal.control + Relation.control +
Health.control + Emot.control + Other.control + Work.prog +
Resp.prog + Leisure.prog + Personal.prog + Relation.prog +
Health.prog + Emot.prog + Other.prog + Work.content +
Resp.content + Leisure.content + Personal.content + Relation.content +
Health.content + Emot.content + Other.content + livingwell , data = WB.base.filt.V2)
```

|  | Overall (N=195) |
| --- | --- |
| Work.time |  |
| 1 | 19 (9.7%) |
| 2 | 19 (9.7%) |
| 3 | 13 (6.7%) |
| 4 | 84 (43.1%) |
| 5 | 60 (30.8%) |
| Resp.time |  |
| 1 | 5 (2.6%) |
| 2 | 27 (13.8%) |
| 3 | 21 (10.8%) |
| 4 | 95 (48.7%) |
| 5 | 47 (24.1%) |
| Leisure.time |  |
| 1 | 11 (5.6%) |
| 2 | 36 (18.5%) |
| 3 | 20 (10.3%) |
| 4 | 90 (46.2%) |
| 5 | 38 (19.5%) |
| Personal.time |  |
| 1 | 10 (5.1%) |
| 2 | 32 (16.4%) |
| 3 | 17 (8.7%) |
| 4 | 96 (49.2%) |
| 5 | 40 (20.5%) |
| Relation.time |  |
| 1 | 8 (4.1%) |
| 2 | 22 (11.3%) |
| 3 | 17 (8.7%) |
| 4 | 99 (50.8%) |
| 5 | 49 (25.1%) |
| Health.time |  |
| 1 | 10 (5.1%) |
| 2 | 44 (22.6%) |
| 3 | 33 (16.9%) |
| 4 | 81 (41.5%) |
| 5 | 27 (13.8%) |
| Emot.time |  |
| 1 | 7 (3.6%) |
| 2 | 39 (20.0%) |
| 3 | 24 (12.3%) |
| 4 | 92 (47.2%) |
| 5 | 33 (16.9%) |
| Other.time |  |
| 1 | 10 (5.1%) |
| 2 | 24 (12.3%) |
| 3 | 76 (39.0%) |
| 4 | 58 (29.7%) |
| 5 | 26 (13.3%) |
| Missing | 1 (0.5%) |
| Work.control |  |
| 1 | 37 (19.0%) |
| 2 | 29 (14.9%) |
| 3 | 18 (9.2%) |
| 4 | 68 (34.9%) |
| 5 | 42 (21.5%) |
| Missing | 1 (0.5%) |
| Resp.control |  |
| 1 | 10 (5.1%) |
| 2 | 27 (13.8%) |
| 3 | 16 (8.2%) |
| 4 | 97 (49.7%) |
| 5 | 44 (22.6%) |
| Missing | 1 (0.5%) |
| Leisure.control |  |
| 1 | 13 (6.7%) |
| 2 | 31 (15.9%) |
| 3 | 25 (12.8%) |
| 4 | 82 (42.1%) |
| 5 | 43 (22.1%) |
| Missing | 1 (0.5%) |
| Personal.control |  |
| 1 | 12 (6.2%) |
| 2 | 41 (21.0%) |
| 3 | 19 (9.7%) |
| 4 | 79 (40.5%) |
| 5 | 43 (22.1%) |
| Missing | 1 (0.5%) |
| Relation.control |  |
| 1 | 16 (8.2%) |
| 2 | 30 (15.4%) |
| 3 | 23 (11.8%) |
| 4 | 83 (42.6%) |
| 5 | 42 (21.5%) |
| Missing | 1 (0.5%) |
| Health.control |  |
| 1 | 16 (8.2%) |
| 2 | 37 (19.0%) |
| 3 | 33 (16.9%) |
| 4 | 76 (39.0%) |
| 5 | 32 (16.4%) |
| Missing | 1 (0.5%) |
| Emot.control |  |
| 1 | 22 (11.3%) |
| 2 | 33 (16.9%) |
| 3 | 24 (12.3%) |
| 4 | 81 (41.5%) |
| 5 | 34 (17.4%) |
| Missing | 1 (0.5%) |
| Other.control |  |
| 1 | 14 (7.2%) |
| 2 | 12 (6.2%) |
| 3 | 78 (40.0%) |
| 4 | 58 (29.7%) |
| 5 | 32 (16.4%) |
| Missing | 1 (0.5%) |
| Work.prog |  |
| 1 | 32 (16.4%) |
| 2 | 35 (17.9%) |
| 3 | 25 (12.8%) |
| 4 | 64 (32.8%) |
| 5 | 38 (19.5%) |
| Missing | 1 (0.5%) |
| Resp.prog |  |
| 1 | 15 (7.7%) |
| 2 | 33 (16.9%) |
| 3 | 36 (18.5%) |
| 4 | 85 (43.6%) |
| 5 | 25 (12.8%) |
| Missing | 1 (0.5%) |
| Leisure.prog |  |
| 1 | 18 (9.2%) |
| 2 | 39 (20.0%) |
| 3 | 36 (18.5%) |
| 4 | 74 (37.9%) |
| 5 | 27 (13.8%) |
| Missing | 1 (0.5%) |
| Personal.prog |  |
| 1 | 14 (7.2%) |
| 2 | 40 (20.5%) |
| 3 | 33 (16.9%) |
| 4 | 78 (40.0%) |
| 5 | 29 (14.9%) |
| Missing | 1 (0.5%) |
| Relation.prog |  |
| 1 | 14 (7.2%) |
| 2 | 25 (12.8%) |
| 3 | 42 (21.5%) |
| 4 | 76 (39.0%) |
| 5 | 37 (19.0%) |
| Missing | 1 (0.5%) |
| Health.prog |  |
| 1 | 21 (10.8%) |
| 2 | 44 (22.6%) |
| 3 | 43 (22.1%) |
| 4 | 63 (32.3%) |
| 5 | 23 (11.8%) |
| Missing | 1 (0.5%) |
| Emot.prog |  |
| 1 | 17 (8.7%) |
| 2 | 37 (19.0%) |
| 3 | 35 (17.9%) |
| 4 | 77 (39.5%) |
| 5 | 28 (14.4%) |
| Missing | 1 (0.5%) |
| Other.prog |  |
| 1 | 15 (7.7%) |
| 2 | 20 (10.3%) |
| 3 | 88 (45.1%) |
| 4 | 46 (23.6%) |
| 5 | 25 (12.8%) |
| Missing | 1 (0.5%) |
| Work.content |  |
| 1 | 46 (23.6%) |
| 2 | 30 (15.4%) |
| 3 | 23 (11.8%) |
| 4 | 63 (32.3%) |
| 5 | 32 (16.4%) |
| Missing | 1 (0.5%) |
| Resp.content |  |
| 1 | 21 (10.8%) |
| 2 | 42 (21.5%) |
| 3 | 23 (11.8%) |
| 4 | 83 (42.6%) |
| 5 | 25 (12.8%) |
| Missing | 1 (0.5%) |
| Leisure.content |  |
| 1 | 22 (11.3%) |
| 2 | 35 (17.9%) |
| 3 | 23 (11.8%) |
| 4 | 93 (47.7%) |
| 5 | 21 (10.8%) |
| Missing | 1 (0.5%) |
| Personal.content |  |
| 1 | 26 (13.3%) |
| 2 | 42 (21.5%) |
| 3 | 23 (11.8%) |
| 4 | 78 (40.0%) |
| 5 | 25 (12.8%) |
| Missing | 1 (0.5%) |
| Relation.content |  |
| 1 | 21 (10.8%) |
| 2 | 34 (17.4%) |
| 3 | 22 (11.3%) |
| 4 | 84 (43.1%) |
| 5 | 33 (16.9%) |
| Missing | 1 (0.5%) |
| Health.content |  |
| 1 | 31 (15.9%) |
| 2 | 56 (28.7%) |
| 3 | 31 (15.9%) |
| 4 | 58 (29.7%) |
| 5 | 18 (9.2%) |
| Missing | 1 (0.5%) |
| Emot.content |  |
| 1 | 25 (12.8%) |
| 2 | 40 (20.5%) |
| 3 | 24 (12.3%) |
| 4 | 76 (39.0%) |
| 5 | 29 (14.9%) |
| Missing | 1 (0.5%) |
| Other.content |  |
| 1 | 17 (8.7%) |
| 2 | 20 (10.3%) |
| 3 | 80 (41.0%) |
| 4 | 54 (27.7%) |
| 5 | 23 (11.8%) |
| Missing | 1 (0.5%) |
| livingwell |  |
| 1 | 21 (10.8%) |
| 2 | 34 (17.4%) |
| 3 | 23 (11.8%) |
| 4 | 84 (43.1%) |
| 5 | 32 (16.4%) |
| Missing | 1 (0.5%) |

### 3.2 Stacked bar plot - Q1 (time)

```
timedf <- WB.base.filt.V2[c("Work.time", "Resp.time", "Leisure.time", "Personal.time", "Relation.time", "Health.time", "Emot.time", "Other.time")]

# Reshape data from wide to long format
df_long <- gather(timedf, key = "Variable", value = "Response")

df_long <- df_long %>%
  mutate(Response = case_when(
    Response == 1 ~ "Strongly Disagree",
    Response == 2 ~ "Disagree",
    Response == 3 ~ "Undecided",
    Response == 4 ~ "Agree",
    Response == 5 ~ "Strongly Agree"
  ))


# Reorder levels of Response variable
df_long$Response <- factor(df_long$Response, 
                           levels = c("Strongly Agree", "Agree", "Undecided", "Disagree", "Strongly Disagree"))

# Plotting using ggplot2
plot.q1 <- ggplot(df_long, aes(x = Variable, y = ..count.., fill = as.factor(Response))) +
  
  # Horizontal layered bar graph
  geom_bar(position = "stack", alpha = 0.8) +
  
  # Adjusting bar width and colors
  scale_fill_viridis_d(name = "Response") +
  
  # Styling and labels
  theme_minimal() +
  labs(
    x = "Domain",
    y = "Count",
    fill = "Response",
    title = "Over the last month, I have had enough time to spend on:"
  ) +
  
  # Flip the coordinates to make it horizontal
  coord_flip()

# Display the plot
print(plot.q1)
```

### 3.3 Stacked bar plot - Q2 (control)

```
timedf2 <- WB.base.filt.V2[c("Work.control", "Resp.control", "Leisure.control", "Personal.control", "Relation.control", "Health.control", "Emot.control", "Other.control")]

# Reshape data from wide to long format
df_long2 <- gather(timedf2, key = "Variable", value = "Response")

df_long2 <- df_long2 %>%
  mutate(Response = case_when(
    Response == 1 ~ "Strongly Disagree",
    Response == 2 ~ "Disagree",
    Response == 3 ~ "Undecided",
    Response == 4 ~ "Agree",
    Response == 5 ~ "Strongly Agree"
  ))


# Reorder levels of Response variable
df_long2$Response <- factor(df_long2$Response, 
                           levels = c("Strongly Agree", "Agree", "Undecided", "Disagree", "Strongly Disagree"))

# Plotting using ggplot2
plot.q2 <- ggplot(df_long2, aes(x = Variable, y = ..count.., fill = as.factor(Response))) +
  
  # Horizontal layered bar graph
  geom_bar(position = "stack", alpha = 0.8) +
  
  # Adjusting bar width and colors
  scale_fill_viridis_d(name = "Response") +
  
  # Styling and labels
  theme_minimal() +
  labs(
    x = "Domain",
    y = "Count",
    fill = "Response",
    title = "Over the last month, I have had a sense of control over my:"
  ) +
  
  # Flip the coordinates to make it horizontal
  coord_flip()

# Display the plot
print(plot.q2)
```

### 3.4 Stacked bar plot - Q3 (progress)

```
timedf3 <- WB.base.filt.V2[c("Work.prog", "Resp.prog", "Leisure.prog", "Personal.prog", "Relation.prog", "Health.prog", "Emot.prog", "Other.prog")]

# Reshape data from wide to long format
df_long3 <- gather(timedf3, key = "Variable", value = "Response")

df_long3 <- df_long3 %>%
  mutate(Response = case_when(
    Response == 1 ~ "Strongly Disagree",
    Response == 2 ~ "Disagree",
    Response == 3 ~ "Undecided",
    Response == 4 ~ "Agree",
    Response == 5 ~ "Strongly Agree"
  ))


# Reorder levels of Response variable
df_long3$Response <- factor(df_long3$Response, 
                           levels = c("Strongly Agree", "Agree", "Undecided", "Disagree", "Strongly Disagree"))

# Plotting using ggplot2
plot.q3 <- ggplot(df_long3, aes(x = Variable, y = ..count.., fill = as.factor(Response))) +
  
  # Horizontal layered bar graph
  geom_bar(position = "stack", alpha = 0.8) +
  
  # Adjusting bar width and colors
  scale_fill_viridis_d(name = "Response") +
  
  # Styling and labels
  theme_minimal() +
  labs(
    x = "Domain",
    y = "Count",
    fill = "Response",
    title = "Over the last month, I have made progress on or had personal growth
    related to:"
  ) +
  
  # Flip the coordinates to make it horizontal
  coord_flip()

# Display the plot
print(plot.q3)
```

### 3.5 Stacked bar plot - Q4 (content)

```
timedf4 <- WB.base.filt.V2[c("Work.content", "Resp.content", "Leisure.content", "Personal.content", "Relation.content", "Health.content", "Emot.content", "Other.content")]

# Reshape data from wide to long format
df_long4 <- gather(timedf4, key = "Variable", value = "Response")

df_long4 <- df_long4 %>%
  mutate(Response = case_when(
    Response == 1 ~ "Strongly Disagree",
    Response == 2 ~ "Disagree",
    Response == 3 ~ "Undecided",
    Response == 4 ~ "Agree",
    Response == 5 ~ "Strongly Agree"
  ))


# Reorder levels of Response variable
df_long4$Response <- factor(df_long4$Response, 
                           levels = c("Strongly Agree", "Agree", "Undecided", "Disagree", "Strongly Disagree"))

# Plotting using ggplot2
plot.q4 <- ggplot(df_long4, aes(x = Variable, y = ..count.., fill = as.factor(Response))) +
  
  # Horizontal layered bar graph
  geom_bar(position = "stack", alpha = 0.8) +
  
  # Adjusting bar width and colors
  scale_fill_viridis_d(name = "Response") +
  
  # Styling and labels
  theme_minimal() +
  labs(
    x = "Domain",
    y = "Count",
    fill = "Response",
    title = "Over the last month, I feel content withhow I'm doing in the following areas:"
  ) +
  
  # Flip the coordinates to make it horizontal
  coord_flip()

# Display the plot
print(plot.q4)
```

### 3.6 Stacked bar plot - Q5 (living well)

```
timedf5 <- WB.base.filt.V2[c("livingwell")]

# Reshape data from wide to long format
df_long5 <- gather(timedf5, key = "Variable", value = "Response")

df_long5 <- df_long5 %>%
  mutate(Response = case_when(
    Response == 1 ~ "Strongly Disagree",
    Response == 2 ~ "Disagree",
    Response == 3 ~ "Undecided",
    Response == 4 ~ "Agree",
    Response == 5 ~ "Strongly Agree"
  ))

# Reorder levels of Response variable
df_long5$Response <- factor(df_long5$Response, 
                           levels = c("Strongly Agree", "Agree", "Undecided", "Disagree", "Strongly Disagree"))

# Plotting using ggplot2
plot.q5 <- ggplot(df_long5, aes(x = Variable, y = ..count.., fill = as.factor(Response))) +
  
  # Horizontal layered bar graph
  geom_bar(position = "stack", alpha = 0.8) +
  
  # Adjusting bar width and colors
  scale_fill_viridis_d(name = "Response") +
  
  # Styling and labels
  theme_minimal() +
  labs(
    x = "Domain",
    y = "Count",
    fill = "Response",
    title = "Over the last month, I have been living well."
  ) +
  
  # Flip the coordinates to make it horizontal
  coord_flip() +
  
  # Adjust the aspect ratio
  theme(aspect.ratio = 1/4)  # You can adjust the ratio as needed

# Display the plot
print(plot.q5)
```

## 4 Parallel Analysis

```
## Make these variables ordinal 

variable_names_int <- c("Work.time", "Resp.time", "Leisure.time", "Personal.time", "Relation.time",
                    "Health.time", "Emot.time", "Other.time", "Work.control",
                    "Resp.control", "Leisure.control", "Personal.control", "Relation.control",
                    "Health.control", "Emot.control", "Other.control", "Work.prog",
                    "Resp.prog", "Leisure.prog", "Personal.prog", "Relation.prog",
                    "Health.prog", "Emot.prog", "Other.prog", "Work.content",
                    "Resp.content", "Leisure.content", "Personal.content", "Relation.content",
                    "Health.content", "Emot.content", "Other.content", "livingwell")

# Convert each variable to factor
WB.base.filt.V2[variable_names_int] <- lapply(WB.base.filt.V2[variable_names_int], as.numeric)
## Create data frame of items only 
WB.EFA <- WB.base.filt.V2[c(4:36)]

## Use only complete data

WB.EFA <- WB.EFA[complete.cases(WB.EFA), ]

## we are going to remove the "other" items

exclude_columns <- c("Other.time", "Other.control", "Other.prog", "Other.content")

WB.EFA.2 <- WB.EFA[, -which(names(WB.EFA) %in% exclude_columns)]
names(WB.EFA.2)
```

```
##  [1] "Work.time"        "Resp.time"        "Leisure.time"     "Personal.time"   
##  [5] "Relation.time"    "Health.time"      "Emot.time"        "Work.control"    
##  [9] "Resp.control"     "Leisure.control"  "Personal.control" "Relation.control"
## [13] "Health.control"   "Emot.control"     "Work.prog"        "Resp.prog"       
## [17] "Leisure.prog"     "Personal.prog"    "Relation.prog"    "Health.prog"     
## [21] "Emot.prog"        "Work.content"     "Resp.content"     "Leisure.content" 
## [25] "Personal.content" "Relation.content" "Health.content"   "Emot.content"    
## [29] "livingwell"
```

```
# Perform parallel analysis using fa.parallel()
pa_result <- fa.parallel(WB.EFA.2, fm = "ml", fa = "both", n.iter = 1000, show.legend = TRUE)
```

```
## Parallel analysis suggests that the number of factors =  6  and the number of components =  3
```

```
pa_result
```

```
## Call: fa.parallel(x = WB.EFA.2, fm = "ml", fa = "both", n.iter = 1000, 
##     show.legend = TRUE)
## Parallel analysis suggests that the number of factors =  6  and the number of components =  3 
## 
##  Eigen Values of 
##   Original factors Resampled data Simulated data Original components
## 1            12.81           0.95           0.95               13.33
## 2             1.59           0.71           0.71                2.26
## 3             1.26           0.62           0.61                1.92
## 4             0.98           0.54           0.54                1.57
## 5             0.73           0.47           0.47                1.27
## 6             0.63           0.41           0.41                1.20
##   Resampled components Simulated components
## 1                 1.80                 1.80
## 2                 1.68                 1.68
## 3                 1.59                 1.59
## 4                 1.52                 1.52
## 5                 1.45                 1.45
## 6                 1.39                 1.39
```

### 4.1 Factor analysis

```
library(psych)

# -----------------------------
# User settings
# -----------------------------

data_for_fa <- WB.EFA.2   # replace raw_data with your dataset name
max_factors <- 6

loading_cutoff <- 0.30    # loading size considered meaningful
diff_cutoff <- 0.20       # primary vs secondary loading difference threshold

# -----------------------------
# Run 1- through 6-factor models
# -----------------------------

fa_models_oblimin <- lapply(1:max_factors, function(nf) {
  fa(
    r = data_for_fa,
    nfactors = nf,
    fm = "ml",
    rotate = "oblimin"
  )
})

names(fa_models_oblimin) <- paste0("FA_", 1:max_factors, "_factors_oblimin")
```

### 4.2 Fit indices

```
fit_indices_table <- do.call(rbind, lapply(1:max_factors, function(nf) {
  
  model <- fa_models_oblimin[[nf]]
  
  data.frame(
    Factors = nf,
    Chi_Square = model$STATISTIC,
    df = model$dof,
    p_value = model$PVAL,
    RMSEA = model$RMSEA[1],
    RMSEA_lower = model$RMSEA[2],
    RMSEA_upper = model$RMSEA[3],
    TLI = model$TLI,
    BIC = model$BIC,
    RMSR = model$rms,
    Fit = model$fit,
    Fit_off_diagonal = model$fit.off
  )
}))

# Round for easier reading
fit_indices_table_round <- fit_indices_table
fit_indices_table_round[, -1] <- round(fit_indices_table_round[, -1], 4)

print(fit_indices_table_round)
```

```
##        Factors Chi_Square  df p_value  RMSEA RMSEA_lower RMSEA_upper    TLI
## RMSEA        1  2011.4225 377       0 0.1498      0.1438      0.1568 0.5851
## RMSEA1       2  1695.5480 349       0 0.1413      0.1350      0.1485 0.6293
## RMSEA2       3  1398.3845 322       0 0.1315      0.1249      0.1391 0.6775
## RMSEA3       4  1159.3708 296       0 0.1228      0.1158      0.1308 0.7174
## RMSEA4       5   947.9166 271       0 0.1136      0.1062      0.1220 0.7570
## RMSEA5       6   752.2076 247       0 0.1028      0.0948      0.1117 0.8002
##              BIC   RMSR    Fit Fit_off_diagonal
## RMSEA    27.3883 0.1030 0.9060           0.9482
## RMSEA1 -141.1309 0.0840 0.9316           0.9656
## RMSEA2 -296.2018 0.0673 0.9506           0.9779
## RMSEA3 -398.3855 0.0562 0.9614           0.9846
## RMSEA4 -478.2725 0.0476 0.9690           0.9890
## RMSEA5 -547.6769 0.0352 0.9781           0.9940
```

```
write.csv(fit_indices_table_round, file = "Fit.indices.csv")
```

### 4.3 Function for table with cross loadings

```
create_loading_flag_table <- function(fa_object, nfactors, loading_cutoff = 0.30, diff_cutoff = 0.20, max_factors = 7) {
  
  # Extract pattern loadings
  L <- as.data.frame(unclass(fa_object$loadings))
  
  # Rename columns consistently as Factor_1, Factor_2, etc.
  names(L) <- paste0("Factor_", 1:ncol(L))
  
  items <- rownames(L)
  
  # Add missing factor columns so all models can be combined
  for (f in 1:max_factors) {
    colname <- paste0("Factor_", f)
    if (!colname %in% names(L)) {
      L[[colname]] <- NA
    }
  }
  
  # Keep factor columns in order
  factor_cols <- paste0("Factor_", 1:max_factors)
  L <- L[, factor_cols]
  
  # Use only the actually estimated factors for flagging
  actual_factor_cols <- paste0("Factor_", 1:nfactors)
  L_actual <- L[, actual_factor_cols, drop = FALSE]
  
  abs_L <- abs(L_actual)
  
  # Primary factor and loading
  primary_factor <- apply(abs_L, 1, function(x) names(x)[which.max(x)])
  primary_loading_abs <- apply(abs_L, 1, max)
  
  primary_loading_signed <- sapply(1:nrow(L_actual), function(i) {
    pf <- primary_factor[i]
    L_actual[i, pf]
  })
  
  # Second-highest loading
  second_loading_abs <- apply(abs_L, 1, function(x) {
    if (length(x) > 1) {
      sort(x, decreasing = TRUE)[2]
    } else {
      NA
    }
  })
  
  second_factor <- apply(abs_L, 1, function(x) {
    if (length(x) > 1) {
      names(sort(x, decreasing = TRUE))[2]
    } else {
      NA
    }
  })
  
  second_loading_signed <- sapply(1:nrow(L_actual), function(i) {
    sf <- second_factor[i]
    if (is.na(sf)) {
      NA
    } else {
      L_actual[i, sf]
    }
  })
  
  # Count loadings above cutoff
  number_loadings_above_cutoff <- apply(abs_L, 1, function(x) {
    sum(x >= loading_cutoff, na.rm = TRUE)
  })
  
  # Cross-loading flag: 2 or more loadings >= cutoff
  cross_loading_flag <- number_loadings_above_cutoff >= 2
  
  # Ambiguous item flag: primary and secondary loadings are close
  ambiguous_item_flag <- ifelse(
    is.na(second_loading_abs),
    FALSE,
    primary_loading_abs - second_loading_abs < diff_cutoff
  )
  
  # Weak item flag: no loading reaches cutoff
  weak_item_flag <- primary_loading_abs < loading_cutoff
  
  # Combine into one table
  output <- data.frame(
    Number_of_Factors = nfactors,
    Item = items,
    L,
    Primary_Factor = primary_factor,
    Primary_Loading = primary_loading_signed,
    Primary_Loading_abs = primary_loading_abs,
    Second_Factor = second_factor,
    Second_Loading = second_loading_signed,
    Second_Loading_abs = second_loading_abs,
    Loading_Difference_abs = primary_loading_abs - second_loading_abs,
    Number_Loadings_Above_Cutoff = number_loadings_above_cutoff,
    Cross_Loading_Flag = cross_loading_flag,
    Ambiguous_Item_Flag = ambiguous_item_flag,
    Weak_Item_Flag = weak_item_flag,
    row.names = NULL
  )
  
  return(output)
}
```

### 4.4 Loadings table

```
loadings_flag_table <- do.call(rbind, lapply(1:max_factors, function(nf) {
  create_loading_flag_table(
    fa_object = fa_models_oblimin[[nf]],
    nfactors = nf,
    loading_cutoff = loading_cutoff,
    diff_cutoff = diff_cutoff,
    max_factors = max_factors
  )
}))

# Round numeric columns for readability
loadings_flag_table_round <- loadings_flag_table

numeric_cols <- sapply(loadings_flag_table_round, is.numeric)
loadings_flag_table_round[, numeric_cols] <- round(loadings_flag_table_round[, numeric_cols], 4)

print(loadings_flag_table_round)
```

```
##     Number_of_Factors             Item Factor_1 Factor_2 Factor_3 Factor_4
## 1                   1        Work.time   0.4194       NA       NA       NA
## 2                   1        Resp.time   0.4618       NA       NA       NA
## 3                   1     Leisure.time   0.5414       NA       NA       NA
## 4                   1    Personal.time   0.5720       NA       NA       NA
## 5                   1    Relation.time   0.5790       NA       NA       NA
## 6                   1      Health.time   0.3767       NA       NA       NA
## 7                   1        Emot.time   0.5828       NA       NA       NA
## 8                   1     Work.control   0.5479       NA       NA       NA
## 9                   1     Resp.control   0.6914       NA       NA       NA
## 10                  1  Leisure.control   0.7189       NA       NA       NA
## 11                  1 Personal.control   0.7960       NA       NA       NA
## 12                  1 Relation.control   0.6891       NA       NA       NA
## 13                  1   Health.control   0.6386       NA       NA       NA
## 14                  1     Emot.control   0.7458       NA       NA       NA
## 15                  1        Work.prog   0.5113       NA       NA       NA
## 16                  1        Resp.prog   0.5590       NA       NA       NA
## 17                  1     Leisure.prog   0.7344       NA       NA       NA
## 18                  1    Personal.prog   0.7519       NA       NA       NA
## 19                  1    Relation.prog   0.6821       NA       NA       NA
## 20                  1      Health.prog   0.5839       NA       NA       NA
## 21                  1        Emot.prog   0.8102       NA       NA       NA
## 22                  1     Work.content   0.6387       NA       NA       NA
## 23                  1     Resp.content   0.7547       NA       NA       NA
## 24                  1  Leisure.content   0.7774       NA       NA       NA
## 25                  1 Personal.content   0.8424       NA       NA       NA
## 26                  1 Relation.content   0.7388       NA       NA       NA
## 27                  1   Health.content   0.6371       NA       NA       NA
## 28                  1     Emot.content   0.7972       NA       NA       NA
## 29                  1       livingwell   0.7398       NA       NA       NA
## 30                  2        Work.time   0.3257   0.1604       NA       NA
## 31                  2        Resp.time   0.1573   0.4732       NA       NA
## 32                  2     Leisure.time   0.0131   0.8024       NA       NA
## 33                  2    Personal.time   0.0019   0.8686       NA       NA
## 34                  2    Relation.time   0.2175   0.5484       NA       NA
## 35                  2      Health.time  -0.0483   0.6408       NA       NA
## 36                  2        Emot.time   0.2184   0.5583       NA       NA
## 37                  2     Work.control   0.6414  -0.1130       NA       NA
## 38                  2     Resp.control   0.8026  -0.1460       NA       NA
## 39                  2  Leisure.control   0.5092   0.3228       NA       NA
## 40                  2 Personal.control   0.6324   0.2513       NA       NA
## 41                  2 Relation.control   0.6512   0.0634       NA       NA
## 42                  2   Health.control   0.5964   0.0722       NA       NA
## 43                  2     Emot.control   0.7466   0.0146       NA       NA
## 44                  2        Work.prog   0.6426  -0.1709       NA       NA
## 45                  2        Resp.prog   0.6527  -0.1202       NA       NA
## 46                  2     Leisure.prog   0.6362   0.1551       NA       NA
## 47                  2    Personal.prog   0.6708   0.1273       NA       NA
## 48                  2    Relation.prog   0.6603   0.0431       NA       NA
## 49                  2      Health.prog   0.5387   0.0745       NA       NA
## 50                  2        Emot.prog   0.8114   0.0133       NA       NA
## 51                  2     Work.content   0.7676  -0.1625       NA       NA
## 52                  2     Resp.content   0.8275  -0.0858       NA       NA
## 53                  2  Leisure.content   0.6123   0.2525       NA       NA
## 54                  2 Personal.content   0.7143   0.2008       NA       NA
## 55                  2 Relation.content   0.6918   0.0785       NA       NA
## 56                  2   Health.content   0.5954   0.0705       NA       NA
## 57                  2     Emot.content   0.7668   0.0600       NA       NA
## 58                  2       livingwell   0.8077  -0.0819       NA       NA
## 59                  3        Work.time  -0.1994   0.3617   0.7109       NA
## 60                  3        Resp.time  -0.0032   0.5423   0.2363       NA
## 61                  3     Leisure.time   0.1281   0.7496  -0.0599       NA
## 62                  3    Personal.time   0.1068   0.8058  -0.0273       NA
## 63                  3    Relation.time   0.1937   0.5539   0.0857       NA
## 64                  3      Health.time  -0.0028   0.6280  -0.0089       NA
## 65                  3        Emot.time   0.0879   0.6121   0.2159       NA
## 66                  3     Work.control   0.1230   0.0305   0.7527       NA
## 67                  3     Resp.control   0.6433  -0.1236   0.2366       NA
## 68                  3  Leisure.control   0.5884   0.2522  -0.0201       NA
## 69                  3 Personal.control   0.7438   0.1608  -0.0574       NA
## 70                  3 Relation.control   0.6055   0.0629   0.0766       NA
## 71                  3   Health.control   0.5510   0.0589   0.0980       NA
## 72                  3     Emot.control   0.5973   0.0435   0.2178       NA
## 73                  3        Work.prog   0.1919  -0.0531   0.6547       NA
## 74                  3        Resp.prog   0.4873  -0.0941   0.2512       NA
## 75                  3     Leisure.prog   0.7875   0.0482  -0.1134       NA
## 76                  3    Personal.prog   0.8406   0.0125  -0.1370       NA
## 77                  3    Relation.prog   0.6663   0.0074   0.0339       NA
## 78                  3      Health.prog   0.5852   0.0267  -0.0209       NA
## 79                  3        Emot.prog   0.7548  -0.0072   0.1218       NA
## 80                  3     Work.content   0.2951  -0.0522   0.7089       NA
## 81                  3     Resp.content   0.7087  -0.0909   0.2115       NA
## 82                  3  Leisure.content   0.7997   0.1278  -0.1408       NA
## 83                  3 Personal.content   0.8373   0.0972  -0.0619       NA
## 84                  3 Relation.content   0.6887   0.0534   0.0360       NA
## 85                  3   Health.content   0.6451   0.0082  -0.0032       NA
## 86                  3     Emot.content   0.6886   0.0511   0.1487       NA
## 87                  3       livingwell   0.7207  -0.0999   0.1676       NA
## 88                  4        Work.time  -0.2600   0.0845   0.7106   0.2936
## 89                  4        Resp.time   0.0985  -0.0924   0.2621   0.5183
## 90                  4     Leisure.time   0.1401   0.0866  -0.0526   0.7171
## 91                  4    Personal.time   0.1951  -0.0161  -0.0122   0.7833
## 92                  4    Relation.time  -0.1416   0.5290   0.0556   0.5004
## 93                  4      Health.time   0.2005  -0.2013   0.0139   0.6340
## 94                  4        Emot.time  -0.0569   0.2579   0.2128   0.5588
## 95                  4     Work.control   0.0624   0.0106   0.7893   0.0043
## 96                  4     Resp.control   0.5775   0.0292   0.3010  -0.0987
## 97                  4  Leisure.control   0.5792   0.0219   0.0317   0.2751
## 98                  4 Personal.control   0.6346   0.1449  -0.0055   0.1851
## 99                  4 Relation.control   0.0589   0.7777   0.0488  -0.0007
## 100                 4   Health.control   0.5524  -0.0314   0.1536   0.0940
## 101                 4     Emot.control   0.3753   0.2667   0.2556   0.0366
## 102                 4        Work.prog   0.0988   0.0332   0.6965  -0.0607
## 103                 4        Resp.prog   0.4552  -0.0290   0.3140  -0.0634
## 104                 4     Leisure.prog   0.6694   0.1242  -0.0511   0.0920
## 105                 4    Personal.prog   0.6544   0.2290  -0.0851   0.0424
## 106                 4    Relation.prog   0.1632   0.6886   0.0240  -0.0311
## 107                 4      Health.prog   0.5606   0.0129   0.0284   0.0636
## 108                 4        Emot.prog   0.4458   0.3754   0.1609  -0.0070
## 109                 4     Work.content   0.2047   0.0303   0.7614  -0.0620
## 110                 4     Resp.content   0.6718  -0.0066   0.2855  -0.0596
## 111                 4  Leisure.content   0.7507   0.0632  -0.0823   0.1713
## 112                 4 Personal.content   0.7034   0.1634  -0.0037   0.1295
## 113                 4 Relation.content   0.0981   0.8582  -0.0004  -0.0189
## 114                 4   Health.content   0.6616  -0.0471   0.0550   0.0545
## 115                 4     Emot.content   0.3963   0.3686   0.1816   0.0416
## 116                 4       livingwell   0.4951   0.2473   0.2160  -0.0848
## 117                 5        Work.time   0.2918   0.6878  -0.1245   0.0496
## 118                 5        Resp.time   0.5429   0.2527   0.0639  -0.1043
## 119                 5     Leisure.time   0.7987  -0.0500  -0.0373   0.0180
## 120                 5    Personal.time   0.8332  -0.0297   0.1056  -0.0294
## 121                 5    Relation.time   0.5516   0.0549  -0.1895   0.4672
## 122                 5      Health.time   0.6636   0.0100   0.0960  -0.1924
## 123                 5        Emot.time   0.5888   0.1937  -0.0031   0.2156
## 124                 5     Work.control   0.0036   0.7793   0.1360   0.0283
## 125                 5     Resp.control  -0.0802   0.3134   0.4812   0.1009
## 126                 5  Leisure.control   0.3245   0.0183   0.4981   0.0704
## 127                 5 Personal.control   0.2306  -0.0139   0.5237   0.2037
## 128                 5 Relation.control   0.0049   0.0124   0.2175   0.8194
## 129                 5   Health.control   0.1071   0.1423   0.5522   0.0615
## 130                 5     Emot.control   0.0348   0.2284   0.5427   0.3419
## 131                 5        Work.prog  -0.0395   0.7763  -0.1206  -0.0118
## 132                 5        Resp.prog  -0.0097   0.4214  -0.0103  -0.0766
## 133                 5     Leisure.prog   0.1847   0.0298   0.1201   0.0796
## 134                 5    Personal.prog   0.1273  -0.0083   0.1263   0.1854
## 135                 5    Relation.prog   0.0226   0.0961  -0.2402   0.6462
## 136                 5      Health.prog   0.1154   0.0903   0.2012   0.0205
## 137                 5        Emot.prog   0.0487   0.2145   0.1913   0.3491
## 138                 5     Work.content  -0.0371   0.7877   0.1305   0.0230
## 139                 5     Resp.content   0.0030   0.3453   0.3552   0.0057
## 140                 5  Leisure.content   0.2565  -0.0430   0.4385   0.0763
## 141                 5 Personal.content   0.2040   0.0352   0.4574   0.1733
## 142                 5 Relation.content   0.0294   0.0187   0.0789   0.7933
## 143                 5   Health.content   0.1123   0.0977   0.3787  -0.0119
## 144                 5     Emot.content   0.0791   0.1914   0.3909   0.3786
## 145                 5       livingwell  -0.0331   0.2615   0.2633   0.2608
## 146                 6        Work.time   0.6811   0.0663  -0.1971   0.0083
## 147                 6        Resp.time   0.2703  -0.0773   0.0808   0.0600
## 148                 6     Leisure.time  -0.0019   0.0356   0.1659  -0.1073
## 149                 6    Personal.time  -0.0080   0.0102   0.1799   0.0989
## 150                 6    Relation.time   0.0542   0.4973  -0.1106  -0.0469
## 151                 6      Health.time  -0.0431  -0.1496  -0.0691   0.5290
## 152                 6        Emot.time   0.2000   0.2540  -0.0130   0.0355
## 153                 6     Work.control   0.8007   0.0168   0.0998  -0.0285
## 154                 6     Resp.control   0.3365   0.0849   0.4867   0.1137
## 155                 6  Leisure.control   0.0703   0.0450   0.6777  -0.0559
## 156                 6 Personal.control   0.0186   0.1999   0.6125   0.0813
## 157                 6 Relation.control  -0.0039   0.8370   0.1564   0.0040
## 158                 6   Health.control   0.1031   0.0841   0.3684   0.4827
## 159                 6     Emot.control   0.2379   0.3517   0.4455   0.1021
## 160                 6        Work.prog   0.7981  -0.0190  -0.0980  -0.0060
## 161                 6        Resp.prog   0.4476  -0.0772   0.0576   0.1293
## 162                 6     Leisure.prog   0.0660   0.0844   0.2630   0.1670
## 163                 6    Personal.prog   0.0192   0.1994   0.2461   0.1871
## 164                 6    Relation.prog   0.0884   0.6628  -0.1660   0.0760
## 165                 6      Health.prog  -0.0040   0.0667  -0.1080   0.8747
## 166                 6        Emot.prog   0.2187   0.3779   0.1550   0.2145
## 167                 6     Work.content   0.8152   0.0151   0.1035   0.0209
## 168                 6     Resp.content   0.3691   0.0144   0.3445   0.2256
## 169                 6  Leisure.content  -0.0052   0.0791   0.5470   0.1496
## 170                 6 Personal.content   0.0710   0.1925   0.5003   0.1581
## 171                 6 Relation.content   0.0021   0.8253   0.0394   0.0648
## 172                 6   Health.content   0.0267   0.0232   0.1268   0.7666
## 173                 6     Emot.content   0.2000   0.4093   0.3001   0.1571
## 174                 6       livingwell   0.2538   0.2794   0.1732   0.2928
##     Factor_5 Factor_6 Primary_Factor Primary_Loading Primary_Loading_abs
## 1         NA       NA       Factor_1          0.4194              0.4194
## 2         NA       NA       Factor_1          0.4618              0.4618
## 3         NA       NA       Factor_1          0.5414              0.5414
## 4         NA       NA       Factor_1          0.5720              0.5720
## 5         NA       NA       Factor_1          0.5790              0.5790
## 6         NA       NA       Factor_1          0.3767              0.3767
## 7         NA       NA       Factor_1          0.5828              0.5828
## 8         NA       NA       Factor_1          0.5479              0.5479
## 9         NA       NA       Factor_1          0.6914              0.6914
## 10        NA       NA       Factor_1          0.7189              0.7189
## 11        NA       NA       Factor_1          0.7960              0.7960
## 12        NA       NA       Factor_1          0.6891              0.6891
## 13        NA       NA       Factor_1          0.6386              0.6386
## 14        NA       NA       Factor_1          0.7458              0.7458
## 15        NA       NA       Factor_1          0.5113              0.5113
## 16        NA       NA       Factor_1          0.5590              0.5590
## 17        NA       NA       Factor_1          0.7344              0.7344
## 18        NA       NA       Factor_1          0.7519              0.7519
## 19        NA       NA       Factor_1          0.6821              0.6821
## 20        NA       NA       Factor_1          0.5839              0.5839
## 21        NA       NA       Factor_1          0.8102              0.8102
## 22        NA       NA       Factor_1          0.6387              0.6387
## 23        NA       NA       Factor_1          0.7547              0.7547
## 24        NA       NA       Factor_1          0.7774              0.7774
## 25        NA       NA       Factor_1          0.8424              0.8424
## 26        NA       NA       Factor_1          0.7388              0.7388
## 27        NA       NA       Factor_1          0.6371              0.6371
## 28        NA       NA       Factor_1          0.7972              0.7972
## 29        NA       NA       Factor_1          0.7398              0.7398
## 30        NA       NA       Factor_1          0.3257              0.3257
## 31        NA       NA       Factor_2          0.4732              0.4732
## 32        NA       NA       Factor_2          0.8024              0.8024
## 33        NA       NA       Factor_2          0.8686              0.8686
## 34        NA       NA       Factor_2          0.5484              0.5484
## 35        NA       NA       Factor_2          0.6408              0.6408
## 36        NA       NA       Factor_2          0.5583              0.5583
## 37        NA       NA       Factor_1          0.6414              0.6414
## 38        NA       NA       Factor_1          0.8026              0.8026
## 39        NA       NA       Factor_1          0.5092              0.5092
## 40        NA       NA       Factor_1          0.6324              0.6324
## 41        NA       NA       Factor_1          0.6512              0.6512
## 42        NA       NA       Factor_1          0.5964              0.5964
## 43        NA       NA       Factor_1          0.7466              0.7466
## 44        NA       NA       Factor_1          0.6426              0.6426
## 45        NA       NA       Factor_1          0.6527              0.6527
## 46        NA       NA       Factor_1          0.6362              0.6362
## 47        NA       NA       Factor_1          0.6708              0.6708
## 48        NA       NA       Factor_1          0.6603              0.6603
## 49        NA       NA       Factor_1          0.5387              0.5387
## 50        NA       NA       Factor_1          0.8114              0.8114
## 51        NA       NA       Factor_1          0.7676              0.7676
## 52        NA       NA       Factor_1          0.8275              0.8275
## 53        NA       NA       Factor_1          0.6123              0.6123
## 54        NA       NA       Factor_1          0.7143              0.7143
## 55        NA       NA       Factor_1          0.6918              0.6918
## 56        NA       NA       Factor_1          0.5954              0.5954
## 57        NA       NA       Factor_1          0.7668              0.7668
## 58        NA       NA       Factor_1          0.8077              0.8077
## 59        NA       NA       Factor_3          0.7109              0.7109
## 60        NA       NA       Factor_2          0.5423              0.5423
## 61        NA       NA       Factor_2          0.7496              0.7496
## 62        NA       NA       Factor_2          0.8058              0.8058
## 63        NA       NA       Factor_2          0.5539              0.5539
## 64        NA       NA       Factor_2          0.6280              0.6280
## 65        NA       NA       Factor_2          0.6121              0.6121
## 66        NA       NA       Factor_3          0.7527              0.7527
## 67        NA       NA       Factor_1          0.6433              0.6433
## 68        NA       NA       Factor_1          0.5884              0.5884
## 69        NA       NA       Factor_1          0.7438              0.7438
## 70        NA       NA       Factor_1          0.6055              0.6055
## 71        NA       NA       Factor_1          0.5510              0.5510
## 72        NA       NA       Factor_1          0.5973              0.5973
## 73        NA       NA       Factor_3          0.6547              0.6547
## 74        NA       NA       Factor_1          0.4873              0.4873
## 75        NA       NA       Factor_1          0.7875              0.7875
## 76        NA       NA       Factor_1          0.8406              0.8406
## 77        NA       NA       Factor_1          0.6663              0.6663
## 78        NA       NA       Factor_1          0.5852              0.5852
## 79        NA       NA       Factor_1          0.7548              0.7548
## 80        NA       NA       Factor_3          0.7089              0.7089
## 81        NA       NA       Factor_1          0.7087              0.7087
## 82        NA       NA       Factor_1          0.7997              0.7997
## 83        NA       NA       Factor_1          0.8373              0.8373
## 84        NA       NA       Factor_1          0.6887              0.6887
## 85        NA       NA       Factor_1          0.6451              0.6451
## 86        NA       NA       Factor_1          0.6886              0.6886
## 87        NA       NA       Factor_1          0.7207              0.7207
## 88        NA       NA       Factor_3          0.7106              0.7106
## 89        NA       NA       Factor_4          0.5183              0.5183
## 90        NA       NA       Factor_4          0.7171              0.7171
## 91        NA       NA       Factor_4          0.7833              0.7833
## 92        NA       NA       Factor_2          0.5290              0.5290
## 93        NA       NA       Factor_4          0.6340              0.6340
## 94        NA       NA       Factor_4          0.5588              0.5588
## 95        NA       NA       Factor_3          0.7893              0.7893
## 96        NA       NA       Factor_1          0.5775              0.5775
## 97        NA       NA       Factor_1          0.5792              0.5792
## 98        NA       NA       Factor_1          0.6346              0.6346
## 99        NA       NA       Factor_2          0.7777              0.7777
## 100       NA       NA       Factor_1          0.5524              0.5524
## 101       NA       NA       Factor_1          0.3753              0.3753
## 102       NA       NA       Factor_3          0.6965              0.6965
## 103       NA       NA       Factor_1          0.4552              0.4552
## 104       NA       NA       Factor_1          0.6694              0.6694
## 105       NA       NA       Factor_1          0.6544              0.6544
## 106       NA       NA       Factor_2          0.6886              0.6886
## 107       NA       NA       Factor_1          0.5606              0.5606
## 108       NA       NA       Factor_1          0.4458              0.4458
## 109       NA       NA       Factor_3          0.7614              0.7614
## 110       NA       NA       Factor_1          0.6718              0.6718
## 111       NA       NA       Factor_1          0.7507              0.7507
## 112       NA       NA       Factor_1          0.7034              0.7034
## 113       NA       NA       Factor_2          0.8582              0.8582
## 114       NA       NA       Factor_1          0.6616              0.6616
## 115       NA       NA       Factor_1          0.3963              0.3963
## 116       NA       NA       Factor_1          0.4951              0.4951
## 117  -0.2194       NA       Factor_2          0.6878              0.6878
## 118  -0.0248       NA       Factor_1          0.5429              0.5429
## 119   0.1272       NA       Factor_1          0.7987              0.7987
## 120   0.0202       NA       Factor_1          0.8332              0.8332
## 121  -0.0090       NA       Factor_1          0.5516              0.5516
## 122   0.0289       NA       Factor_1          0.6636              0.6636
## 123  -0.1076       NA       Factor_1          0.5888              0.5888
## 124  -0.1039       NA       Factor_2          0.7793              0.7793
## 125   0.1638       NA       Factor_3          0.4812              0.4812
## 126   0.1270       NA       Factor_3          0.4981              0.4981
## 127   0.1828       NA       Factor_3          0.5237              0.5237
## 128  -0.1138       NA       Factor_4          0.8194              0.8194
## 129   0.0320       NA       Factor_3          0.5522              0.5522
## 130  -0.1149       NA       Factor_3          0.5427              0.5427
## 131   0.1953       NA       Factor_2          0.7763              0.7763
## 132   0.5137       NA       Factor_5          0.5137              0.5137
## 133   0.6523       NA       Factor_5          0.6523              0.6523
## 134   0.6492       NA       Factor_5          0.6492              0.6492
## 135   0.4515       NA       Factor_4          0.6462              0.6462
## 136   0.4018       NA       Factor_5          0.4018              0.4018
## 137   0.3318       NA       Factor_4          0.3491              0.3491
## 138   0.0654       NA       Factor_2          0.7877              0.7877
## 139   0.3748       NA       Factor_5          0.3748              0.3748
## 140   0.3781       NA       Factor_3          0.4385              0.4385
## 141   0.3170       NA       Factor_3          0.4574              0.4574
## 142   0.0995       NA       Factor_4          0.7933              0.7933
## 143   0.3196       NA       Factor_3          0.3787              0.3787
## 144   0.0630       NA       Factor_3          0.3909              0.3909
## 145   0.2858       NA       Factor_5          0.2858              0.2858
## 146   0.2782  -0.1784       Factor_1          0.6811              0.6811
## 147   0.4945  -0.0323       Factor_5          0.4945              0.4945
## 148   0.7647   0.1885       Factor_5          0.7647              0.7647
## 149   0.7395   0.0038       Factor_5          0.7395              0.7395
## 150   0.5324   0.0387       Factor_5          0.5324              0.5324
## 151   0.5798  -0.1411       Factor_5          0.5798              0.5798
## 152   0.5563  -0.0962       Factor_5          0.5563              0.5563
## 153  -0.0091  -0.1123       Factor_1          0.8007              0.8007
## 154  -0.1157   0.0251       Factor_3          0.4867              0.4867
## 155   0.2714   0.0657       Factor_3          0.6777              0.6777
## 156   0.1623   0.0696       Factor_3          0.6125              0.6125
## 157  -0.0034  -0.1414       Factor_2          0.8370              0.8370
## 158  -0.0039  -0.2146       Factor_4          0.4827              0.4827
## 159  -0.0130  -0.2122       Factor_3          0.4455              0.4455
## 160  -0.0209   0.1778       Factor_1          0.7981              0.7981
## 161  -0.0031   0.4115       Factor_1          0.4476              0.4476
## 162   0.1495   0.5330       Factor_6          0.5330              0.5330
## 163   0.0884   0.5166       Factor_6          0.5166              0.5166
## 164   0.0387   0.3982       Factor_2          0.6628              0.6628
## 165  -0.0120   0.1016       Factor_4          0.8747              0.8747
## 166   0.0033   0.2118       Factor_2          0.3779              0.3779
## 167  -0.0460   0.0255       Factor_1          0.8152              0.8152
## 168  -0.0447   0.2130       Factor_1          0.3691              0.3691
## 169   0.1787   0.2490       Factor_3          0.5470              0.5470
## 170   0.1285   0.1866       Factor_3          0.5003              0.5003
## 171   0.0196   0.0575       Factor_2          0.8253              0.8253
## 172  -0.0235   0.0136       Factor_4          0.7666              0.7666
## 173   0.0204  -0.0323       Factor_2          0.4093              0.4093
## 174  -0.0798   0.1335       Factor_4          0.2928              0.2928
##     Second_Factor Second_Loading Second_Loading_abs Loading_Difference_abs
## 1            <NA>             NA                 NA                     NA
## 2            <NA>             NA                 NA                     NA
## 3            <NA>             NA                 NA                     NA
## 4            <NA>             NA                 NA                     NA
## 5            <NA>             NA                 NA                     NA
## 6            <NA>             NA                 NA                     NA
## 7            <NA>             NA                 NA                     NA
## 8            <NA>             NA                 NA                     NA
## 9            <NA>             NA                 NA                     NA
## 10           <NA>             NA                 NA                     NA
## 11           <NA>             NA                 NA                     NA
## 12           <NA>             NA                 NA                     NA
## 13           <NA>             NA                 NA                     NA
## 14           <NA>             NA                 NA                     NA
## 15           <NA>             NA                 NA                     NA
## 16           <NA>             NA                 NA                     NA
## 17           <NA>             NA                 NA                     NA
## 18           <NA>             NA                 NA                     NA
## 19           <NA>             NA                 NA                     NA
## 20           <NA>             NA                 NA                     NA
## 21           <NA>             NA                 NA                     NA
## 22           <NA>             NA                 NA                     NA
## 23           <NA>             NA                 NA                     NA
## 24           <NA>             NA                 NA                     NA
## 25           <NA>             NA                 NA                     NA
## 26           <NA>             NA                 NA                     NA
## 27           <NA>             NA                 NA                     NA
## 28           <NA>             NA                 NA                     NA
## 29           <NA>             NA                 NA                     NA
## 30       Factor_2         0.1604             0.1604                 0.1653
## 31       Factor_1         0.1573             0.1573                 0.3159
## 32       Factor_1         0.0131             0.0131                 0.7894
## 33       Factor_1         0.0019             0.0019                 0.8667
## 34       Factor_1         0.2175             0.2175                 0.3309
## 35       Factor_1        -0.0483             0.0483                 0.5925
## 36       Factor_1         0.2184             0.2184                 0.3399
## 37       Factor_2        -0.1130             0.1130                 0.5284
## 38       Factor_2        -0.1460             0.1460                 0.6567
## 39       Factor_2         0.3228             0.3228                 0.1864
## 40       Factor_2         0.2513             0.2513                 0.3811
## 41       Factor_2         0.0634             0.0634                 0.5879
## 42       Factor_2         0.0722             0.0722                 0.5242
## 43       Factor_2         0.0146             0.0146                 0.7320
## 44       Factor_2        -0.1709             0.1709                 0.4717
## 45       Factor_2        -0.1202             0.1202                 0.5325
## 46       Factor_2         0.1551             0.1551                 0.4811
## 47       Factor_2         0.1273             0.1273                 0.5434
## 48       Factor_2         0.0431             0.0431                 0.6172
## 49       Factor_2         0.0745             0.0745                 0.4642
## 50       Factor_2         0.0133             0.0133                 0.7981
## 51       Factor_2        -0.1625             0.1625                 0.6051
## 52       Factor_2        -0.0858             0.0858                 0.7417
## 53       Factor_2         0.2525             0.2525                 0.3598
## 54       Factor_2         0.2008             0.2008                 0.5135
## 55       Factor_2         0.0785             0.0785                 0.6133
## 56       Factor_2         0.0705             0.0705                 0.5249
## 57       Factor_2         0.0600             0.0600                 0.7068
## 58       Factor_2        -0.0819             0.0819                 0.7258
## 59       Factor_2         0.3617             0.3617                 0.3493
## 60       Factor_3         0.2363             0.2363                 0.3060
## 61       Factor_1         0.1281             0.1281                 0.6215
## 62       Factor_1         0.1068             0.1068                 0.6990
## 63       Factor_1         0.1937             0.1937                 0.3601
## 64       Factor_3        -0.0089             0.0089                 0.6192
## 65       Factor_3         0.2159             0.2159                 0.3962
## 66       Factor_1         0.1230             0.1230                 0.6297
## 67       Factor_3         0.2366             0.2366                 0.4067
## 68       Factor_2         0.2522             0.2522                 0.3363
## 69       Factor_2         0.1608             0.1608                 0.5830
## 70       Factor_3         0.0766             0.0766                 0.5290
## 71       Factor_3         0.0980             0.0980                 0.4530
## 72       Factor_3         0.2178             0.2178                 0.3795
## 73       Factor_1         0.1919             0.1919                 0.4628
## 74       Factor_3         0.2512             0.2512                 0.2362
## 75       Factor_3        -0.1134             0.1134                 0.6740
## 76       Factor_3        -0.1370             0.1370                 0.7036
## 77       Factor_3         0.0339             0.0339                 0.6324
## 78       Factor_2         0.0267             0.0267                 0.5585
## 79       Factor_3         0.1218             0.1218                 0.6330
## 80       Factor_1         0.2951             0.2951                 0.4138
## 81       Factor_3         0.2115             0.2115                 0.4972
## 82       Factor_3        -0.1408             0.1408                 0.6589
## 83       Factor_2         0.0972             0.0972                 0.7401
## 84       Factor_2         0.0534             0.0534                 0.6353
## 85       Factor_2         0.0082             0.0082                 0.6370
## 86       Factor_3         0.1487             0.1487                 0.5400
## 87       Factor_3         0.1676             0.1676                 0.5531
## 88       Factor_4         0.2936             0.2936                 0.4170
## 89       Factor_3         0.2621             0.2621                 0.2562
## 90       Factor_1         0.1401             0.1401                 0.5771
## 91       Factor_1         0.1951             0.1951                 0.5882
## 92       Factor_4         0.5004             0.5004                 0.0286
## 93       Factor_2        -0.2013             0.2013                 0.4326
## 94       Factor_2         0.2579             0.2579                 0.3009
## 95       Factor_1         0.0624             0.0624                 0.7269
## 96       Factor_3         0.3010             0.3010                 0.2765
## 97       Factor_4         0.2751             0.2751                 0.3041
## 98       Factor_4         0.1851             0.1851                 0.4496
## 99       Factor_1         0.0589             0.0589                 0.7189
## 100      Factor_3         0.1536             0.1536                 0.3988
## 101      Factor_2         0.2667             0.2667                 0.1086
## 102      Factor_1         0.0988             0.0988                 0.5976
## 103      Factor_3         0.3140             0.3140                 0.1412
## 104      Factor_2         0.1242             0.1242                 0.5452
## 105      Factor_2         0.2290             0.2290                 0.4254
## 106      Factor_1         0.1632             0.1632                 0.5254
## 107      Factor_4         0.0636             0.0636                 0.4970
## 108      Factor_2         0.3754             0.3754                 0.0704
## 109      Factor_1         0.2047             0.2047                 0.5567
## 110      Factor_3         0.2855             0.2855                 0.3863
## 111      Factor_4         0.1713             0.1713                 0.5794
## 112      Factor_2         0.1634             0.1634                 0.5400
## 113      Factor_1         0.0981             0.0981                 0.7601
## 114      Factor_3         0.0550             0.0550                 0.6066
## 115      Factor_2         0.3686             0.3686                 0.0278
## 116      Factor_2         0.2473             0.2473                 0.2478
## 117      Factor_1         0.2918             0.2918                 0.3960
## 118      Factor_2         0.2527             0.2527                 0.2902
## 119      Factor_5         0.1272             0.1272                 0.6715
## 120      Factor_3         0.1056             0.1056                 0.7276
## 121      Factor_4         0.4672             0.4672                 0.0844
## 122      Factor_4        -0.1924             0.1924                 0.4712
## 123      Factor_4         0.2156             0.2156                 0.3732
## 124      Factor_3         0.1360             0.1360                 0.6433
## 125      Factor_2         0.3134             0.3134                 0.1678
## 126      Factor_1         0.3245             0.3245                 0.1737
## 127      Factor_1         0.2306             0.2306                 0.2930
## 128      Factor_3         0.2175             0.2175                 0.6019
## 129      Factor_2         0.1423             0.1423                 0.4099
## 130      Factor_4         0.3419             0.3419                 0.2008
## 131      Factor_5         0.1953             0.1953                 0.5809
## 132      Factor_2         0.4214             0.4214                 0.0923
## 133      Factor_1         0.1847             0.1847                 0.4676
## 134      Factor_4         0.1854             0.1854                 0.4637
## 135      Factor_5         0.4515             0.4515                 0.1947
## 136      Factor_3         0.2012             0.2012                 0.2006
## 137      Factor_5         0.3318             0.3318                 0.0172
## 138      Factor_3         0.1305             0.1305                 0.6572
## 139      Factor_3         0.3552             0.3552                 0.0196
## 140      Factor_5         0.3781             0.3781                 0.0604
## 141      Factor_5         0.3170             0.3170                 0.1403
## 142      Factor_5         0.0995             0.0995                 0.6938
## 143      Factor_5         0.3196             0.3196                 0.0590
## 144      Factor_4         0.3786             0.3786                 0.0123
## 145      Factor_3         0.2633             0.2633                 0.0225
## 146      Factor_5         0.2782             0.2782                 0.4029
## 147      Factor_1         0.2703             0.2703                 0.2242
## 148      Factor_6         0.1885             0.1885                 0.5762
## 149      Factor_3         0.1799             0.1799                 0.5596
## 150      Factor_2         0.4973             0.4973                 0.0351
## 151      Factor_4         0.5290             0.5290                 0.0509
## 152      Factor_2         0.2540             0.2540                 0.3023
## 153      Factor_6        -0.1123             0.1123                 0.6884
## 154      Factor_1         0.3365             0.3365                 0.1502
## 155      Factor_5         0.2714             0.2714                 0.4063
## 156      Factor_2         0.1999             0.1999                 0.4126
## 157      Factor_3         0.1564             0.1564                 0.6806
## 158      Factor_3         0.3684             0.3684                 0.1143
## 159      Factor_2         0.3517             0.3517                 0.0938
## 160      Factor_6         0.1778             0.1778                 0.6202
## 161      Factor_6         0.4115             0.4115                 0.0360
## 162      Factor_3         0.2630             0.2630                 0.2700
## 163      Factor_3         0.2461             0.2461                 0.2705
## 164      Factor_6         0.3982             0.3982                 0.2646
## 165      Factor_3        -0.1080             0.1080                 0.7668
## 166      Factor_1         0.2187             0.2187                 0.1593
## 167      Factor_3         0.1035             0.1035                 0.7117
## 168      Factor_3         0.3445             0.3445                 0.0245
## 169      Factor_6         0.2490             0.2490                 0.2980
## 170      Factor_2         0.1925             0.1925                 0.3079
## 171      Factor_4         0.0648             0.0648                 0.7605
## 172      Factor_3         0.1268             0.1268                 0.6398
## 173      Factor_3         0.3001             0.3001                 0.1092
## 174      Factor_2         0.2794             0.2794                 0.0133
##     Number_Loadings_Above_Cutoff Cross_Loading_Flag Ambiguous_Item_Flag
## 1                              1              FALSE               FALSE
## 2                              1              FALSE               FALSE
## 3                              1              FALSE               FALSE
## 4                              1              FALSE               FALSE
## 5                              1              FALSE               FALSE
## 6                              1              FALSE               FALSE
## 7                              1              FALSE               FALSE
## 8                              1              FALSE               FALSE
## 9                              1              FALSE               FALSE
## 10                             1              FALSE               FALSE
## 11                             1              FALSE               FALSE
## 12                             1              FALSE               FALSE
## 13                             1              FALSE               FALSE
## 14                             1              FALSE               FALSE
## 15                             1              FALSE               FALSE
## 16                             1              FALSE               FALSE
## 17                             1              FALSE               FALSE
## 18                             1              FALSE               FALSE
## 19                             1              FALSE               FALSE
## 20                             1              FALSE               FALSE
## 21                             1              FALSE               FALSE
## 22                             1              FALSE               FALSE
## 23                             1              FALSE               FALSE
## 24                             1              FALSE               FALSE
## 25                             1              FALSE               FALSE
## 26                             1              FALSE               FALSE
## 27                             1              FALSE               FALSE
## 28                             1              FALSE               FALSE
## 29                             1              FALSE               FALSE
## 30                             1              FALSE                TRUE
## 31                             1              FALSE               FALSE
## 32                             1              FALSE               FALSE
## 33                             1              FALSE               FALSE
## 34                             1              FALSE               FALSE
## 35                             1              FALSE               FALSE
## 36                             1              FALSE               FALSE
## 37                             1              FALSE               FALSE
## 38                             1              FALSE               FALSE
## 39                             2               TRUE                TRUE
## 40                             1              FALSE               FALSE
## 41                             1              FALSE               FALSE
## 42                             1              FALSE               FALSE
## 43                             1              FALSE               FALSE
## 44                             1              FALSE               FALSE
## 45                             1              FALSE               FALSE
## 46                             1              FALSE               FALSE
## 47                             1              FALSE               FALSE
## 48                             1              FALSE               FALSE
## 49                             1              FALSE               FALSE
## 50                             1              FALSE               FALSE
## 51                             1              FALSE               FALSE
## 52                             1              FALSE               FALSE
## 53                             1              FALSE               FALSE
## 54                             1              FALSE               FALSE
## 55                             1              FALSE               FALSE
## 56                             1              FALSE               FALSE
## 57                             1              FALSE               FALSE
## 58                             1              FALSE               FALSE
## 59                             2               TRUE               FALSE
## 60                             1              FALSE               FALSE
## 61                             1              FALSE               FALSE
## 62                             1              FALSE               FALSE
## 63                             1              FALSE               FALSE
## 64                             1              FALSE               FALSE
## 65                             1              FALSE               FALSE
## 66                             1              FALSE               FALSE
## 67                             1              FALSE               FALSE
## 68                             1              FALSE               FALSE
## 69                             1              FALSE               FALSE
## 70                             1              FALSE               FALSE
## 71                             1              FALSE               FALSE
## 72                             1              FALSE               FALSE
## 73                             1              FALSE               FALSE
## 74                             1              FALSE               FALSE
## 75                             1              FALSE               FALSE
## 76                             1              FALSE               FALSE
## 77                             1              FALSE               FALSE
## 78                             1              FALSE               FALSE
## 79                             1              FALSE               FALSE
## 80                             1              FALSE               FALSE
## 81                             1              FALSE               FALSE
## 82                             1              FALSE               FALSE
## 83                             1              FALSE               FALSE
## 84                             1              FALSE               FALSE
## 85                             1              FALSE               FALSE
## 86                             1              FALSE               FALSE
## 87                             1              FALSE               FALSE
## 88                             1              FALSE               FALSE
## 89                             1              FALSE               FALSE
## 90                             1              FALSE               FALSE
## 91                             1              FALSE               FALSE
## 92                             2               TRUE                TRUE
## 93                             1              FALSE               FALSE
## 94                             1              FALSE               FALSE
## 95                             1              FALSE               FALSE
## 96                             2               TRUE               FALSE
## 97                             1              FALSE               FALSE
## 98                             1              FALSE               FALSE
## 99                             1              FALSE               FALSE
## 100                            1              FALSE               FALSE
## 101                            1              FALSE                TRUE
## 102                            1              FALSE               FALSE
## 103                            2               TRUE                TRUE
## 104                            1              FALSE               FALSE
## 105                            1              FALSE               FALSE
## 106                            1              FALSE               FALSE
## 107                            1              FALSE               FALSE
## 108                            2               TRUE                TRUE
## 109                            1              FALSE               FALSE
## 110                            1              FALSE               FALSE
## 111                            1              FALSE               FALSE
## 112                            1              FALSE               FALSE
## 113                            1              FALSE               FALSE
## 114                            1              FALSE               FALSE
## 115                            2               TRUE                TRUE
## 116                            1              FALSE               FALSE
## 117                            1              FALSE               FALSE
## 118                            1              FALSE               FALSE
## 119                            1              FALSE               FALSE
## 120                            1              FALSE               FALSE
## 121                            2               TRUE                TRUE
## 122                            1              FALSE               FALSE
## 123                            1              FALSE               FALSE
## 124                            1              FALSE               FALSE
## 125                            2               TRUE                TRUE
## 126                            2               TRUE                TRUE
## 127                            1              FALSE               FALSE
## 128                            1              FALSE               FALSE
## 129                            1              FALSE               FALSE
## 130                            2               TRUE               FALSE
## 131                            1              FALSE               FALSE
## 132                            2               TRUE                TRUE
## 133                            1              FALSE               FALSE
## 134                            1              FALSE               FALSE
## 135                            2               TRUE                TRUE
## 136                            1              FALSE               FALSE
## 137                            2               TRUE                TRUE
## 138                            1              FALSE               FALSE
## 139                            3               TRUE                TRUE
## 140                            2               TRUE                TRUE
## 141                            2               TRUE                TRUE
## 142                            1              FALSE               FALSE
## 143                            2               TRUE                TRUE
## 144                            2               TRUE                TRUE
## 145                            0              FALSE                TRUE
## 146                            1              FALSE               FALSE
## 147                            1              FALSE               FALSE
## 148                            1              FALSE               FALSE
## 149                            1              FALSE               FALSE
## 150                            2               TRUE                TRUE
## 151                            2               TRUE                TRUE
## 152                            1              FALSE               FALSE
## 153                            1              FALSE               FALSE
## 154                            2               TRUE                TRUE
## 155                            1              FALSE               FALSE
## 156                            1              FALSE               FALSE
## 157                            1              FALSE               FALSE
## 158                            2               TRUE                TRUE
## 159                            2               TRUE                TRUE
## 160                            1              FALSE               FALSE
## 161                            2               TRUE                TRUE
## 162                            1              FALSE               FALSE
## 163                            1              FALSE               FALSE
## 164                            2               TRUE               FALSE
## 165                            1              FALSE               FALSE
## 166                            1              FALSE                TRUE
## 167                            1              FALSE               FALSE
## 168                            2               TRUE                TRUE
## 169                            1              FALSE               FALSE
## 170                            1              FALSE               FALSE
## 171                            1              FALSE               FALSE
## 172                            1              FALSE               FALSE
## 173                            2               TRUE                TRUE
## 174                            0              FALSE                TRUE
##     Weak_Item_Flag
## 1            FALSE
## 2            FALSE
## 3            FALSE
## 4            FALSE
## 5            FALSE
## 6            FALSE
## 7            FALSE
## 8            FALSE
## 9            FALSE
## 10           FALSE
## 11           FALSE
## 12           FALSE
## 13           FALSE
## 14           FALSE
## 15           FALSE
## 16           FALSE
## 17           FALSE
## 18           FALSE
## 19           FALSE
## 20           FALSE
## 21           FALSE
## 22           FALSE
## 23           FALSE
## 24           FALSE
## 25           FALSE
## 26           FALSE
## 27           FALSE
## 28           FALSE
## 29           FALSE
## 30           FALSE
## 31           FALSE
## 32           FALSE
## 33           FALSE
## 34           FALSE
## 35           FALSE
## 36           FALSE
## 37           FALSE
## 38           FALSE
## 39           FALSE
## 40           FALSE
## 41           FALSE
## 42           FALSE
## 43           FALSE
## 44           FALSE
## 45           FALSE
## 46           FALSE
## 47           FALSE
## 48           FALSE
## 49           FALSE
## 50           FALSE
## 51           FALSE
## 52           FALSE
## 53           FALSE
## 54           FALSE
## 55           FALSE
## 56           FALSE
## 57           FALSE
## 58           FALSE
## 59           FALSE
## 60           FALSE
## 61           FALSE
## 62           FALSE
## 63           FALSE
## 64           FALSE
## 65           FALSE
## 66           FALSE
## 67           FALSE
## 68           FALSE
## 69           FALSE
## 70           FALSE
## 71           FALSE
## 72           FALSE
## 73           FALSE
## 74           FALSE
## 75           FALSE
## 76           FALSE
## 77           FALSE
## 78           FALSE
## 79           FALSE
## 80           FALSE
## 81           FALSE
## 82           FALSE
## 83           FALSE
## 84           FALSE
## 85           FALSE
## 86           FALSE
## 87           FALSE
## 88           FALSE
## 89           FALSE
## 90           FALSE
## 91           FALSE
## 92           FALSE
## 93           FALSE
## 94           FALSE
## 95           FALSE
## 96           FALSE
## 97           FALSE
## 98           FALSE
## 99           FALSE
## 100          FALSE
## 101          FALSE
## 102          FALSE
## 103          FALSE
## 104          FALSE
## 105          FALSE
## 106          FALSE
## 107          FALSE
## 108          FALSE
## 109          FALSE
## 110          FALSE
## 111          FALSE
## 112          FALSE
## 113          FALSE
## 114          FALSE
## 115          FALSE
## 116          FALSE
## 117          FALSE
## 118          FALSE
## 119          FALSE
## 120          FALSE
## 121          FALSE
## 122          FALSE
## 123          FALSE
## 124          FALSE
## 125          FALSE
## 126          FALSE
## 127          FALSE
## 128          FALSE
## 129          FALSE
## 130          FALSE
## 131          FALSE
## 132          FALSE
## 133          FALSE
## 134          FALSE
## 135          FALSE
## 136          FALSE
## 137          FALSE
## 138          FALSE
## 139          FALSE
## 140          FALSE
## 141          FALSE
## 142          FALSE
## 143          FALSE
## 144          FALSE
## 145           TRUE
## 146          FALSE
## 147          FALSE
## 148          FALSE
## 149          FALSE
## 150          FALSE
## 151          FALSE
## 152          FALSE
## 153          FALSE
## 154          FALSE
## 155          FALSE
## 156          FALSE
## 157          FALSE
## 158          FALSE
## 159          FALSE
## 160          FALSE
## 161          FALSE
## 162          FALSE
## 163          FALSE
## 164          FALSE
## 165          FALSE
## 166          FALSE
## 167          FALSE
## 168          FALSE
## 169          FALSE
## 170          FALSE
## 171          FALSE
## 172          FALSE
## 173          FALSE
## 174           TRUE
```

```
write.csv(loadings_flag_table_round, file = "Loadings.csv")
```

### 4.5 cross-loaded or problematic items

```
problem_items_table <- subset(
  loadings_flag_table_round,
  Cross_Loading_Flag == TRUE |
    Ambiguous_Item_Flag == TRUE |
    Weak_Item_Flag == TRUE
)

print(problem_items_table)
```

```
##     Number_of_Factors             Item Factor_1 Factor_2 Factor_3 Factor_4
## 30                  2        Work.time   0.3257   0.1604       NA       NA
## 39                  2  Leisure.control   0.5092   0.3228       NA       NA
## 59                  3        Work.time  -0.1994   0.3617   0.7109       NA
## 92                  4    Relation.time  -0.1416   0.5290   0.0556   0.5004
## 96                  4     Resp.control   0.5775   0.0292   0.3010  -0.0987
## 101                 4     Emot.control   0.3753   0.2667   0.2556   0.0366
## 103                 4        Resp.prog   0.4552  -0.0290   0.3140  -0.0634
## 108                 4        Emot.prog   0.4458   0.3754   0.1609  -0.0070
## 115                 4     Emot.content   0.3963   0.3686   0.1816   0.0416
## 121                 5    Relation.time   0.5516   0.0549  -0.1895   0.4672
## 125                 5     Resp.control  -0.0802   0.3134   0.4812   0.1009
## 126                 5  Leisure.control   0.3245   0.0183   0.4981   0.0704
## 130                 5     Emot.control   0.0348   0.2284   0.5427   0.3419
## 132                 5        Resp.prog  -0.0097   0.4214  -0.0103  -0.0766
## 135                 5    Relation.prog   0.0226   0.0961  -0.2402   0.6462
## 137                 5        Emot.prog   0.0487   0.2145   0.1913   0.3491
## 139                 5     Resp.content   0.0030   0.3453   0.3552   0.0057
## 140                 5  Leisure.content   0.2565  -0.0430   0.4385   0.0763
## 141                 5 Personal.content   0.2040   0.0352   0.4574   0.1733
## 143                 5   Health.content   0.1123   0.0977   0.3787  -0.0119
## 144                 5     Emot.content   0.0791   0.1914   0.3909   0.3786
## 145                 5       livingwell  -0.0331   0.2615   0.2633   0.2608
## 150                 6    Relation.time   0.0542   0.4973  -0.1106  -0.0469
## 151                 6      Health.time  -0.0431  -0.1496  -0.0691   0.5290
## 154                 6     Resp.control   0.3365   0.0849   0.4867   0.1137
## 158                 6   Health.control   0.1031   0.0841   0.3684   0.4827
## 159                 6     Emot.control   0.2379   0.3517   0.4455   0.1021
## 161                 6        Resp.prog   0.4476  -0.0772   0.0576   0.1293
## 164                 6    Relation.prog   0.0884   0.6628  -0.1660   0.0760
## 166                 6        Emot.prog   0.2187   0.3779   0.1550   0.2145
## 168                 6     Resp.content   0.3691   0.0144   0.3445   0.2256
## 173                 6     Emot.content   0.2000   0.4093   0.3001   0.1571
## 174                 6       livingwell   0.2538   0.2794   0.1732   0.2928
##     Factor_5 Factor_6 Primary_Factor Primary_Loading Primary_Loading_abs
## 30        NA       NA       Factor_1          0.3257              0.3257
## 39        NA       NA       Factor_1          0.5092              0.5092
## 59        NA       NA       Factor_3          0.7109              0.7109
## 92        NA       NA       Factor_2          0.5290              0.5290
## 96        NA       NA       Factor_1          0.5775              0.5775
## 101       NA       NA       Factor_1          0.3753              0.3753
## 103       NA       NA       Factor_1          0.4552              0.4552
## 108       NA       NA       Factor_1          0.4458              0.4458
## 115       NA       NA       Factor_1          0.3963              0.3963
## 121  -0.0090       NA       Factor_1          0.5516              0.5516
## 125   0.1638       NA       Factor_3          0.4812              0.4812
## 126   0.1270       NA       Factor_3          0.4981              0.4981
## 130  -0.1149       NA       Factor_3          0.5427              0.5427
## 132   0.5137       NA       Factor_5          0.5137              0.5137
## 135   0.4515       NA       Factor_4          0.6462              0.6462
## 137   0.3318       NA       Factor_4          0.3491              0.3491
## 139   0.3748       NA       Factor_5          0.3748              0.3748
## 140   0.3781       NA       Factor_3          0.4385              0.4385
## 141   0.3170       NA       Factor_3          0.4574              0.4574
## 143   0.3196       NA       Factor_3          0.3787              0.3787
## 144   0.0630       NA       Factor_3          0.3909              0.3909
## 145   0.2858       NA       Factor_5          0.2858              0.2858
## 150   0.5324   0.0387       Factor_5          0.5324              0.5324
## 151   0.5798  -0.1411       Factor_5          0.5798              0.5798
## 154  -0.1157   0.0251       Factor_3          0.4867              0.4867
## 158  -0.0039  -0.2146       Factor_4          0.4827              0.4827
## 159  -0.0130  -0.2122       Factor_3          0.4455              0.4455
## 161  -0.0031   0.4115       Factor_1          0.4476              0.4476
## 164   0.0387   0.3982       Factor_2          0.6628              0.6628
## 166   0.0033   0.2118       Factor_2          0.3779              0.3779
## 168  -0.0447   0.2130       Factor_1          0.3691              0.3691
## 173   0.0204  -0.0323       Factor_2          0.4093              0.4093
## 174  -0.0798   0.1335       Factor_4          0.2928              0.2928
##     Second_Factor Second_Loading Second_Loading_abs Loading_Difference_abs
## 30       Factor_2         0.1604             0.1604                 0.1653
## 39       Factor_2         0.3228             0.3228                 0.1864
## 59       Factor_2         0.3617             0.3617                 0.3493
## 92       Factor_4         0.5004             0.5004                 0.0286
## 96       Factor_3         0.3010             0.3010                 0.2765
## 101      Factor_2         0.2667             0.2667                 0.1086
## 103      Factor_3         0.3140             0.3140                 0.1412
## 108      Factor_2         0.3754             0.3754                 0.0704
## 115      Factor_2         0.3686             0.3686                 0.0278
## 121      Factor_4         0.4672             0.4672                 0.0844
## 125      Factor_2         0.3134             0.3134                 0.1678
## 126      Factor_1         0.3245             0.3245                 0.1737
## 130      Factor_4         0.3419             0.3419                 0.2008
## 132      Factor_2         0.4214             0.4214                 0.0923
## 135      Factor_5         0.4515             0.4515                 0.1947
## 137      Factor_5         0.3318             0.3318                 0.0172
## 139      Factor_3         0.3552             0.3552                 0.0196
## 140      Factor_5         0.3781             0.3781                 0.0604
## 141      Factor_5         0.3170             0.3170                 0.1403
## 143      Factor_5         0.3196             0.3196                 0.0590
## 144      Factor_4         0.3786             0.3786                 0.0123
## 145      Factor_3         0.2633             0.2633                 0.0225
## 150      Factor_2         0.4973             0.4973                 0.0351
## 151      Factor_4         0.5290             0.5290                 0.0509
## 154      Factor_1         0.3365             0.3365                 0.1502
## 158      Factor_3         0.3684             0.3684                 0.1143
## 159      Factor_2         0.3517             0.3517                 0.0938
## 161      Factor_6         0.4115             0.4115                 0.0360
## 164      Factor_6         0.3982             0.3982                 0.2646
## 166      Factor_1         0.2187             0.2187                 0.1593
## 168      Factor_3         0.3445             0.3445                 0.0245
## 173      Factor_3         0.3001             0.3001                 0.1092
## 174      Factor_2         0.2794             0.2794                 0.0133
##     Number_Loadings_Above_Cutoff Cross_Loading_Flag Ambiguous_Item_Flag
## 30                             1              FALSE                TRUE
## 39                             2               TRUE                TRUE
## 59                             2               TRUE               FALSE
## 92                             2               TRUE                TRUE
## 96                             2               TRUE               FALSE
## 101                            1              FALSE                TRUE
## 103                            2               TRUE                TRUE
## 108                            2               TRUE                TRUE
## 115                            2               TRUE                TRUE
## 121                            2               TRUE                TRUE
## 125                            2               TRUE                TRUE
## 126                            2               TRUE                TRUE
## 130                            2               TRUE               FALSE
## 132                            2               TRUE                TRUE
## 135                            2               TRUE                TRUE
## 137                            2               TRUE                TRUE
## 139                            3               TRUE                TRUE
## 140                            2               TRUE                TRUE
## 141                            2               TRUE                TRUE
## 143                            2               TRUE                TRUE
## 144                            2               TRUE                TRUE
## 145                            0              FALSE                TRUE
## 150                            2               TRUE                TRUE
## 151                            2               TRUE                TRUE
## 154                            2               TRUE                TRUE
## 158                            2               TRUE                TRUE
## 159                            2               TRUE                TRUE
## 161                            2               TRUE                TRUE
## 164                            2               TRUE               FALSE
## 166                            1              FALSE                TRUE
## 168                            2               TRUE                TRUE
## 173                            2               TRUE                TRUE
## 174                            0              FALSE                TRUE
##     Weak_Item_Flag
## 30           FALSE
## 39           FALSE
## 59           FALSE
## 92           FALSE
## 96           FALSE
## 101          FALSE
## 103          FALSE
## 108          FALSE
## 115          FALSE
## 121          FALSE
## 125          FALSE
## 126          FALSE
## 130          FALSE
## 132          FALSE
## 135          FALSE
## 137          FALSE
## 139          FALSE
## 140          FALSE
## 141          FALSE
## 143          FALSE
## 144          FALSE
## 145           TRUE
## 150          FALSE
## 151          FALSE
## 154          FALSE
## 158          FALSE
## 159          FALSE
## 161          FALSE
## 164          FALSE
## 166          FALSE
## 168          FALSE
## 173          FALSE
## 174           TRUE
```

### 4.6 Print sorted loadings for each model

```
for (nf in 1:max_factors) {
  
  cat("\n\n====================================\n")
  cat("Oblimin EFA:", nf, "factor(s)\n")
  cat("====================================\n")
  
  sorted_model <- fa.sort(fa_models_oblimin[[nf]])
  
  print(sorted_model$loadings, cutoff = loading_cutoff)
}
```

```
## 
## 
## ====================================
## Oblimin EFA: 1 factor(s)
## ====================================
## 
## Loadings:
##                  ML1  
## Personal.content 0.842
## Emot.prog        0.810
## Emot.content     0.797
## Personal.control 0.796
## Leisure.content  0.777
## Resp.content     0.755
## Personal.prog    0.752
## Emot.control     0.746
## livingwell       0.740
## Relation.content 0.739
## Leisure.prog     0.734
## Leisure.control  0.719
## Resp.control     0.691
## Relation.control 0.689
## Relation.prog    0.682
## Work.content     0.639
## Health.control   0.639
## Health.content   0.637
## Health.prog      0.584
## Emot.time        0.583
## Relation.time    0.579
## Personal.time    0.572
## Resp.prog        0.559
## Work.control     0.548
## Leisure.time     0.541
## Work.prog        0.511
## Resp.time        0.462
## Work.time        0.419
## Health.time      0.377
## 
##                   ML1
## SS loadings    12.761
## Proportion Var  0.440
## 
## 
## ====================================
## Oblimin EFA: 2 factor(s)
## ====================================
## 
## Loadings:
##                  ML2    ML1   
## Resp.content      0.827       
## Emot.prog         0.811       
## livingwell        0.808       
## Resp.control      0.803       
## Work.content      0.768       
## Emot.content      0.767       
## Emot.control      0.747       
## Personal.content  0.714       
## Relation.content  0.692       
## Personal.prog     0.671       
## Relation.prog     0.660       
## Resp.prog         0.653       
## Relation.control  0.651       
## Work.prog         0.643       
## Work.control      0.641       
## Leisure.prog      0.636       
## Personal.control  0.632       
## Leisure.content   0.612       
## Health.control    0.596       
## Health.content    0.595       
## Health.prog       0.539       
## Leisure.control   0.509  0.323
## Work.time         0.326       
## Personal.time            0.869
## Leisure.time             0.802
## Health.time              0.641
## Emot.time                0.558
## Relation.time            0.548
## Resp.time                0.473
## 
##                   ML2   ML1
## SS loadings    10.591 3.133
## Proportion Var  0.365 0.108
## Cumulative Var  0.365 0.473
## 
## 
## ====================================
## Oblimin EFA: 3 factor(s)
## ====================================
## 
## Loadings:
##                  ML3    ML1    ML2   
## Personal.prog     0.841              
## Personal.content  0.837              
## Leisure.content   0.800              
## Leisure.prog      0.787              
## Emot.prog         0.755              
## Personal.control  0.744              
## livingwell        0.721              
## Resp.content      0.709              
## Relation.content  0.689              
## Emot.content      0.689              
## Relation.prog     0.666              
## Health.content    0.645              
## Resp.control      0.643              
## Relation.control  0.606              
## Emot.control      0.597              
## Leisure.control   0.588              
## Health.prog       0.585              
## Health.control    0.551              
## Resp.prog         0.487              
## Personal.time            0.806       
## Leisure.time             0.750       
## Health.time              0.628       
## Emot.time                0.612       
## Relation.time            0.554       
## Resp.time                0.542       
## Work.control                    0.753
## Work.time                0.362  0.711
## Work.content                    0.709
## Work.prog                       0.655
## 
##                  ML3   ML1   ML2
## SS loadings    9.240 2.894 2.471
## Proportion Var 0.319 0.100 0.085
## Cumulative Var 0.319 0.418 0.504
## 
## 
## ====================================
## Oblimin EFA: 4 factor(s)
## ====================================
## 
## Loadings:
##                  ML1    ML4    ML3    ML2   
## Leisure.content   0.751                     
## Personal.content  0.703                     
## Resp.content      0.672                     
## Leisure.prog      0.669                     
## Health.content    0.662                     
## Personal.prog     0.654                     
## Personal.control  0.635                     
## Leisure.control   0.579                     
## Resp.control      0.577         0.301       
## Health.prog       0.561                     
## Health.control    0.552                     
## livingwell        0.495                     
## Resp.prog         0.455         0.314       
## Emot.prog         0.446  0.375              
## Emot.content      0.396  0.369              
## Emot.control      0.375                     
## Relation.content         0.858              
## Relation.control         0.778              
## Relation.prog            0.689              
## Relation.time            0.529         0.500
## Work.control                    0.789       
## Work.content                    0.761       
## Work.time                       0.711       
## Work.prog                       0.696       
## Personal.time                          0.783
## Leisure.time                           0.717
## Health.time                            0.634
## Emot.time                              0.559
## Resp.time                              0.518
## 
##                  ML1   ML4   ML3   ML2
## SS loadings    5.758 2.762 2.803 2.666
## Proportion Var 0.199 0.095 0.097 0.092
## Cumulative Var 0.199 0.294 0.390 0.482
## 
## 
## ====================================
## Oblimin EFA: 5 factor(s)
## ====================================
## 
## Loadings:
##                  ML4    ML5    ML3    ML2    ML1   
## Personal.time     0.833                            
## Leisure.time      0.799                            
## Health.time       0.664                            
## Emot.time         0.589                            
## Relation.time     0.552                0.467       
## Resp.time         0.543                            
## Work.content             0.788                     
## Work.control             0.779                     
## Work.prog                0.776                     
## Work.time                0.688                     
## Health.control                  0.552              
## Emot.control                    0.543  0.342       
## Personal.control                0.524              
## Leisure.control   0.324         0.498              
## Resp.control             0.313  0.481              
## Personal.content                0.457         0.317
## Leisure.content                 0.439         0.378
## Emot.content                    0.391  0.379       
## Health.content                  0.379         0.320
## Relation.control                       0.819       
## Relation.content                       0.793       
## Relation.prog                          0.646  0.451
## Emot.prog                              0.349  0.332
## Leisure.prog                                  0.652
## Personal.prog                                 0.649
## Resp.prog                0.421                0.514
## Health.prog                                   0.402
## Resp.content             0.345  0.355         0.375
## livingwell                                         
## 
##                  ML4   ML5   ML3   ML2   ML1
## SS loadings    3.179 3.062 2.593 2.630 2.403
## Proportion Var 0.110 0.106 0.089 0.091 0.083
## Cumulative Var 0.110 0.215 0.305 0.395 0.478
## 
## 
## ====================================
## Oblimin EFA: 6 factor(s)
## ====================================
## 
## Loadings:
##                  ML4    ML5    ML1    ML3    ML6    ML2   
## Work.content      0.815                                   
## Work.control      0.801                                   
## Work.prog         0.798                                   
## Work.time         0.681                                   
## Resp.prog         0.448                              0.412
## Resp.content      0.369         0.345                     
## Relation.control         0.837                            
## Relation.content         0.825                            
## Relation.prog            0.663                       0.398
## Emot.content             0.409  0.300                     
## Emot.prog                0.378                            
## Leisure.control                 0.678                     
## Personal.control                0.612                     
## Leisure.content                 0.547                     
## Personal.content                0.500                     
## Resp.control      0.337         0.487                     
## Emot.control             0.352  0.445                     
## Health.prog                            0.875              
## Health.content                         0.767              
## Health.control                  0.368  0.483              
## livingwell                                                
## Leisure.time                                  0.765       
## Personal.time                                 0.740       
## Health.time                            0.529  0.580       
## Emot.time                                     0.556       
## Relation.time            0.497                0.532       
## Resp.time                                     0.495       
## Leisure.prog                                         0.533
## Personal.prog                                        0.517
## 
##                  ML4   ML5   ML1   ML3   ML6   ML2
## SS loadings    3.217 2.837 2.585 2.273 2.589 1.363
## Proportion Var 0.111 0.098 0.089 0.078 0.089 0.047
## Cumulative Var 0.111 0.209 0.298 0.376 0.466 0.513
```

### 4.7 Factor correlations for each model

```
for (nf in 2:max_factors) {
  
  cat("\n\n====================================\n")
  cat("Factor correlations:", nf, "factor model\n")
  cat("====================================\n")
  
  print(round(fa_models_oblimin[[nf]]$Phi, 4))
}
```

```
## 
## 
## ====================================
## Factor correlations: 2 factor model
## ====================================
##        ML2    ML1
## ML2 1.0000 0.5411
## ML1 0.5411 1.0000
## 
## 
## ====================================
## Factor correlations: 3 factor model
## ====================================
##        ML3    ML1    ML2
## ML3 1.0000 0.5126 0.4569
## ML1 0.5126 1.0000 0.2085
## ML2 0.4569 0.2085 1.0000
## 
## 
## ====================================
## Factor correlations: 4 factor model
## ====================================
##        ML1    ML4    ML3    ML2
## ML1 1.0000 0.5833 0.4453 0.3736
## ML4 0.5833 1.0000 0.4014 0.3636
## ML3 0.4453 0.4014 1.0000 0.2312
## ML2 0.3736 0.3636 0.2312 1.0000
## 
## 
## ====================================
## Factor correlations: 5 factor model
## ====================================
##        ML4    ML5    ML3    ML2    ML1
## ML4 1.0000 0.3084 0.3361 0.4041 0.3063
## ML5 0.3084 1.0000 0.3621 0.4066 0.3146
## ML3 0.3361 0.3621 1.0000 0.3677 0.3556
## ML2 0.4041 0.4066 0.3677 1.0000 0.4086
## ML1 0.3063 0.3146 0.3556 0.4086 1.0000
## 
## 
## ====================================
## Factor correlations: 6 factor model
## ====================================
##        ML4    ML5    ML1    ML3    ML6    ML2
## ML4 1.0000 0.4513 0.3803 0.3723 0.2327 0.1811
## ML5 0.4513 1.0000 0.4450 0.3692 0.3126 0.2953
## ML1 0.3803 0.4450 1.0000 0.4133 0.2653 0.2157
## ML3 0.3723 0.3692 0.4133 1.0000 0.3036 0.2457
## ML6 0.2327 0.3126 0.2653 0.3036 1.0000 0.1102
## ML2 0.1811 0.2953 0.2157 0.2457 0.1102 1.0000
```

##Classical Test Theory

### 4.8 item-scale correlations

```
# Assuming 'data' is your data frame containing the variables of interest
totalcor <- WB.base.filt.V2[c(4:10,12:18,20:26,28:34,36)]

# Calculate the correlation matrix
cor_matrix_short <- cor(totalcor, use = "complete.obs")
cor_matrix_short
```

```
##                  Work.time Resp.time Leisure.time Personal.time Relation.time
## Work.time        1.0000000 0.4369636    0.3249806     0.3080164     0.3816303
## Resp.time        0.4369636 1.0000000    0.4793927     0.5426761     0.4094890
## Leisure.time     0.3249806 0.4793927    1.0000000     0.6953489     0.5889410
## Personal.time    0.3080164 0.5426761    0.6953489     1.0000000     0.5482196
## Relation.time    0.3816303 0.4094890    0.5889410     0.5482196     1.0000000
## Health.time      0.2426300 0.4221326    0.4414005     0.5718932     0.3819408
## Emot.time        0.4234449 0.4317188    0.5765574     0.5808871     0.5628283
## Work.control     0.6030941 0.2796362    0.1989722     0.2611164     0.2651387
## Resp.control     0.3005981 0.4139866    0.2336507     0.2298852     0.2540289
## Leisure.control  0.2314916 0.3887757    0.5337440     0.5083401     0.4307746
## Personal.control 0.2396024 0.3643583    0.4321300     0.5358574     0.4310266
## Relation.control 0.3146154 0.2675258    0.3164336     0.3183049     0.5627329
## Health.control   0.2972129 0.2689407    0.2309212     0.3592006     0.3266122
## Emot.control     0.3469437 0.3532857    0.3045704     0.3702495     0.3483486
## Work.prog        0.4818004 0.2233842    0.1598125     0.1791600     0.2844772
## Resp.prog        0.2672631 0.3888556    0.2372837     0.1753985     0.2637915
## Leisure.prog     0.1826841 0.2729732    0.4831666     0.3988420     0.4266152
## Personal.prog    0.1966377 0.3147740    0.4326975     0.4141024     0.3610918
## Relation.prog    0.2549526 0.2068076    0.3445806     0.3295652     0.5688619
## Health.prog      0.2313106 0.2852909    0.2164272     0.3363074     0.2605151
## Emot.prog        0.3264678 0.3169667    0.3599587     0.4032892     0.4206332
## Work.content     0.5428370 0.2816655    0.2196245     0.2618610     0.3162884
## Resp.content     0.3361480 0.4571963    0.3320463     0.3108434     0.2963342
## Leisure.content  0.2140417 0.2363304    0.5204358     0.4936588     0.4490268
## Personal.content 0.2292416 0.3122927    0.4673856     0.5370859     0.4576335
## Relation.content 0.2994490 0.2398585    0.3778919     0.3544468     0.5776240
## Health.content   0.2279398 0.2030313    0.2658097     0.3560024     0.2838360
## Emot.content     0.3281485 0.3421500    0.3626781     0.4335086     0.4204743
## livingwell       0.2629497 0.2865934    0.3370461     0.2947652     0.3606924
##                  Health.time Emot.time Work.control Resp.control
## Work.time          0.2426300 0.4234449    0.6030941    0.3005981
## Resp.time          0.4221326 0.4317188    0.2796362    0.4139866
## Leisure.time       0.4414005 0.5765574    0.1989722    0.2336507
## Personal.time      0.5718932 0.5808871    0.2611164    0.2298852
## Relation.time      0.3819408 0.5628283    0.2651387    0.2540289
## Health.time        1.0000000 0.5176718    0.1040820    0.1888496
## Emot.time          0.5176718 1.0000000    0.3474050    0.3026321
## Work.control       0.1040820 0.3474050    1.0000000    0.5016471
## Resp.control       0.1888496 0.3026321    0.5016471    1.0000000
## Leisure.control    0.2998085 0.4207961    0.3805462    0.6300796
## Personal.control   0.3292566 0.4322968    0.3816587    0.6090691
## Relation.control   0.1682439 0.4029250    0.3810727    0.4898303
## Health.control     0.4049047 0.3272007    0.3890314    0.5793591
## Emot.control       0.2479285 0.4974581    0.4752110    0.6516184
## Work.prog          0.1110642 0.2837622    0.6039858    0.3904057
## Resp.prog          0.2315630 0.2515339    0.3461095    0.5224240
## Leisure.prog       0.2688395 0.3375424    0.2802470    0.4750848
## Personal.prog      0.2344919 0.3695621    0.3087617    0.4991116
## Relation.prog      0.1457627 0.4035101    0.3135119    0.4256009
## Health.prog        0.4806785 0.2852560    0.2333437    0.3711956
## Emot.prog          0.1852036 0.5114544    0.4566292    0.4966817
## Work.content       0.1405639 0.3826215    0.7367924    0.5127475
## Resp.content       0.2397947 0.3237419    0.4485438    0.6950039
## Leisure.content    0.2736490 0.3904734    0.3495016    0.5006396
## Personal.content   0.2794044 0.4449595    0.3933647    0.5113510
## Relation.content   0.1597659 0.4641643    0.3408837    0.4536469
## Health.content     0.4472704 0.2761133    0.3394793    0.4186287
## Emot.content       0.1830207 0.5068810    0.4312247    0.4647741
## livingwell         0.2181236 0.3570481    0.4355360    0.5161496
##                  Leisure.control Personal.control Relation.control
## Work.time              0.2314916        0.2396024        0.3146154
## Resp.time              0.3887757        0.3643583        0.2675258
## Leisure.time           0.5337440        0.4321300        0.3164336
## Personal.time          0.5083401        0.5358574        0.3183049
## Relation.time          0.4307746        0.4310266        0.5627329
## Health.time            0.2998085        0.3292566        0.1682439
## Emot.time              0.4207961        0.4322968        0.4029250
## Work.control           0.3805462        0.3816587        0.3810727
## Resp.control           0.6300796        0.6090691        0.4898303
## Leisure.control        1.0000000        0.7682627        0.4699206
## Personal.control       0.7682627        1.0000000        0.5996157
## Relation.control       0.4699206        0.5996157        1.0000000
## Health.control         0.5154102        0.5689275        0.4463747
## Emot.control           0.5991102        0.6303601        0.6177964
## Work.prog              0.2656444        0.3024903        0.2687934
## Resp.prog              0.3540130        0.3673008        0.2639645
## Leisure.prog           0.5495606        0.5527746        0.4176232
## Personal.prog          0.5155356        0.6710875        0.4701077
## Relation.prog          0.3869989        0.4816035        0.6197717
## Health.prog            0.3028245        0.4190295        0.3151069
## Emot.prog              0.4949133        0.5904127        0.5245396
## Work.content           0.4121285        0.4577236        0.3697424
## Resp.content           0.5124743        0.5461085        0.4257651
## Leisure.content        0.6746198        0.6889546        0.4534730
## Personal.content       0.6053951        0.7246092        0.5403954
## Relation.content       0.4587427        0.5483593        0.7813890
## Health.content         0.3890322        0.4582404        0.3260694
## Emot.content           0.4812726        0.5686798        0.6013467
## livingwell             0.4576546        0.5304289        0.5616216
##                  Health.control Emot.control Work.prog Resp.prog Leisure.prog
## Work.time             0.2972129    0.3469437 0.4818004 0.2672631    0.1826841
## Resp.time             0.2689407    0.3532857 0.2233842 0.3888556    0.2729732
## Leisure.time          0.2309212    0.3045704 0.1598125 0.2372837    0.4831666
## Personal.time         0.3592006    0.3702495 0.1791600 0.1753985    0.3988420
## Relation.time         0.3266122    0.3483486 0.2844772 0.2637915    0.4266152
## Health.time           0.4049047    0.2479285 0.1110642 0.2315630    0.2688395
## Emot.time             0.3272007    0.4974581 0.2837622 0.2515339    0.3375424
## Work.control          0.3890314    0.4752110 0.6039858 0.3461095    0.2802470
## Resp.control          0.5793591    0.6516184 0.3904057 0.5224240    0.4750848
## Leisure.control       0.5154102    0.5991102 0.2656444 0.3540130    0.5495606
## Personal.control      0.5689275    0.6303601 0.3024903 0.3673008    0.5527746
## Relation.control      0.4463747    0.6177964 0.2687934 0.2639645    0.4176232
## Health.control        1.0000000    0.6028029 0.3017429 0.2894793    0.3716464
## Emot.control          0.6028029    1.0000000 0.3616331 0.3800081    0.4460263
## Work.prog             0.3017429    0.3616331 1.0000000 0.5434585    0.3418788
## Resp.prog             0.2894793    0.3800081 0.5434585 1.0000000    0.5417895
## Leisure.prog          0.3716464    0.4460263 0.3418788 0.5417895    1.0000000
## Personal.prog         0.4260816    0.4264982 0.3523673 0.5094952    0.7287645
## Relation.prog         0.3009110    0.4211423 0.4071952 0.4569164    0.5892617
## Health.prog           0.5313280    0.3706003 0.2704738 0.3861025    0.4785921
## Emot.prog             0.4783589    0.6537522 0.4515769 0.4571346    0.6334912
## Work.content          0.3783663    0.5102604 0.6609794 0.4581904    0.3860396
## Resp.content          0.4868765    0.5226520 0.3941760 0.6256682    0.5622711
## Leisure.content       0.5063880    0.4952780 0.2719353 0.3827458    0.6462428
## Personal.content      0.5255521    0.5955393 0.3928233 0.3975350    0.6206163
## Relation.content      0.3728927    0.5527670 0.3130993 0.3302289    0.4789912
## Health.content        0.5937496    0.3878643 0.2315303 0.2912854    0.4703943
## Emot.content          0.4733877    0.7317348 0.3842687 0.3644468    0.5308481
## livingwell            0.4560062    0.5607064 0.4290333 0.4393241    0.5482112
##                  Personal.prog Relation.prog Health.prog Emot.prog Work.content
## Work.time            0.1966377     0.2549526   0.2313106 0.3264678    0.5428370
## Resp.time            0.3147740     0.2068076   0.2852909 0.3169667    0.2816655
## Leisure.time         0.4326975     0.3445806   0.2164272 0.3599587    0.2196245
## Personal.time        0.4141024     0.3295652   0.3363074 0.4032892    0.2618610
## Relation.time        0.3610918     0.5688619   0.2605151 0.4206332    0.3162884
## Health.time          0.2344919     0.1457627   0.4806785 0.1852036    0.1405639
## Emot.time            0.3695621     0.4035101   0.2852560 0.5114544    0.3826215
## Work.control         0.3087617     0.3135119   0.2333437 0.4566292    0.7367924
## Resp.control         0.4991116     0.4256009   0.3711956 0.4966817    0.5127475
## Leisure.control      0.5155356     0.3869989   0.3028245 0.4949133    0.4121285
## Personal.control     0.6710875     0.4816035   0.4190295 0.5904127    0.4577236
## Relation.control     0.4701077     0.6197717   0.3151069 0.5245396    0.3697424
## Health.control       0.4260816     0.3009110   0.5313280 0.4783589    0.3783663
## Emot.control         0.4264982     0.4211423   0.3706003 0.6537522    0.5102604
## Work.prog            0.3523673     0.4071952   0.2704738 0.4515769    0.6609794
## Resp.prog            0.5094952     0.4569164   0.3861025 0.4571346    0.4581904
## Leisure.prog         0.7287645     0.5892617   0.4785921 0.6334912    0.3860396
## Personal.prog        1.0000000     0.6582780   0.5196763 0.6561947    0.3784893
## Relation.prog        0.6582780     1.0000000   0.4233128 0.6636616    0.4049565
## Health.prog          0.5196763     0.4233128   1.0000000 0.5181923    0.3099426
## Emot.prog            0.6561947     0.6636616   0.5181923 1.0000000    0.5144407
## Work.content         0.3784893     0.4049565   0.3099426 0.5144407    1.0000000
## Resp.content         0.5510132     0.4694865   0.4619343 0.5902862    0.6225346
## Leisure.content      0.6234896     0.4669752   0.4267380 0.5739108    0.4175793
## Personal.content     0.6808147     0.4936666   0.4487165 0.6641848    0.4796678
## Relation.content     0.5645697     0.7177161   0.3936072 0.6504963    0.4237391
## Health.content       0.4596950     0.3705942   0.7255751 0.5158479    0.3931623
## Emot.content         0.4938362     0.4906068   0.4153599 0.7704670    0.5424488
## livingwell           0.5517561     0.5413929   0.5160846 0.6103012    0.5825835
##                  Resp.content Leisure.content Personal.content Relation.content
## Work.time           0.3361480       0.2140417        0.2292416        0.2994490
## Resp.time           0.4571963       0.2363304        0.3122927        0.2398585
## Leisure.time        0.3320463       0.5204358        0.4673856        0.3778919
## Personal.time       0.3108434       0.4936588        0.5370859        0.3544468
## Relation.time       0.2963342       0.4490268        0.4576335        0.5776240
## Health.time         0.2397947       0.2736490        0.2794044        0.1597659
## Emot.time           0.3237419       0.3904734        0.4449595        0.4641643
## Work.control        0.4485438       0.3495016        0.3933647        0.3408837
## Resp.control        0.6950039       0.5006396        0.5113510        0.4536469
## Leisure.control     0.5124743       0.6746198        0.6053951        0.4587427
## Personal.control    0.5461085       0.6889546        0.7246092        0.5483593
## Relation.control    0.4257651       0.4534730        0.5403954        0.7813890
## Health.control      0.4868765       0.5063880        0.5255521        0.3728927
## Emot.control        0.5226520       0.4952780        0.5955393        0.5527670
## Work.prog           0.3941760       0.2719353        0.3928233        0.3130993
## Resp.prog           0.6256682       0.3827458        0.3975350        0.3302289
## Leisure.prog        0.5622711       0.6462428        0.6206163        0.4789912
## Personal.prog       0.5510132       0.6234896        0.6808147        0.5645697
## Relation.prog       0.4694865       0.4669752        0.4936666        0.7177161
## Health.prog         0.4619343       0.4267380        0.4487165        0.3936072
## Emot.prog           0.5902862       0.5739108        0.6641848        0.6504963
## Work.content        0.6225346       0.4175793        0.4796678        0.4237391
## Resp.content        1.0000000       0.6164059        0.6752895        0.5123767
## Leisure.content     0.6164059       1.0000000        0.7894298        0.5317012
## Personal.content    0.6752895       0.7894298        1.0000000        0.6125792
## Relation.content    0.5123767       0.5317012        0.6125792        1.0000000
## Health.content      0.5436428       0.5857885        0.5606821        0.4182573
## Emot.content        0.5755704       0.5963736        0.7320955        0.6539894
## livingwell          0.6128525       0.5358514        0.6226620        0.5567263
##                  Health.content Emot.content livingwell
## Work.time             0.2279398    0.3281485  0.2629497
## Resp.time             0.2030313    0.3421500  0.2865934
## Leisure.time          0.2658097    0.3626781  0.3370461
## Personal.time         0.3560024    0.4335086  0.2947652
## Relation.time         0.2838360    0.4204743  0.3606924
## Health.time           0.4472704    0.1830207  0.2181236
## Emot.time             0.2761133    0.5068810  0.3570481
## Work.control          0.3394793    0.4312247  0.4355360
## Resp.control          0.4186287    0.4647741  0.5161496
## Leisure.control       0.3890322    0.4812726  0.4576546
## Personal.control      0.4582404    0.5686798  0.5304289
## Relation.control      0.3260694    0.6013467  0.5616216
## Health.control        0.5937496    0.4733877  0.4560062
## Emot.control          0.3878643    0.7317348  0.5607064
## Work.prog             0.2315303    0.3842687  0.4290333
## Resp.prog             0.2912854    0.3644468  0.4393241
## Leisure.prog          0.4703943    0.5308481  0.5482112
## Personal.prog         0.4596950    0.4938362  0.5517561
## Relation.prog         0.3705942    0.4906068  0.5413929
## Health.prog           0.7255751    0.4153599  0.5160846
## Emot.prog             0.5158479    0.7704670  0.6103012
## Work.content          0.3931623    0.5424488  0.5825835
## Resp.content          0.5436428    0.5755704  0.6128525
## Leisure.content       0.5857885    0.5963736  0.5358514
## Personal.content      0.5606821    0.7320955  0.6226620
## Relation.content      0.4182573    0.6539894  0.5567263
## Health.content        1.0000000    0.5166172  0.5699094
## Emot.content          0.5166172    1.0000000  0.6069268
## livingwell            0.5699094    0.6069268  1.0000000
```

```
write.csv(cor_matrix_short, file = "cor_matrix.csv")

# Calculate p-values for correlation coefficients
library(Hmisc)
p_values_short <- rcorr(as.matrix(totalcor), type = "pearson")$P
write.csv(p_values_short, "pvalues_short.csv")
```

No items were dropped based on item-scale correlations

### 4.9 Create subscales

```
## Create subscales scores in reduced data: 

WB.base.filt.V2$wellbeing <- (WB.base.filt.V2$Resp.control + WB.base.filt.V2$Leisure.control + WB.base.filt.V2$Personal.control + WB.base.filt.V2$Relation.control + WB.base.filt.V2$Health.control + WB.base.filt.V2$Emot.control + WB.base.filt.V2$Resp.prog + WB.base.filt.V2$Leisure.prog + WB.base.filt.V2$Personal.prog + WB.base.filt.V2$Relation.prog + WB.base.filt.V2$Health.prog + WB.base.filt.V2$Emot.prog + 
WB.base.filt.V2$Resp.content + WB.base.filt.V2$Leisure.content + WB.base.filt.V2$Personal.content + WB.base.filt.V2$Relation.content + WB.base.filt.V2$Health.content + WB.base.filt.V2$Emot.content + WB.base.filt.V2$livingwell)/19

WB.base.filt.V2$workfunctioning <- (WB.base.filt.V2$Work.time + WB.base.filt.V2$Work.control + WB.base.filt.V2$Work.prog + WB.base.filt.V2$Work.content)/4

WB.base.filt.V2$enoughtime <- (WB.base.filt.V2$Resp.time + WB.base.filt.V2$Leisure.time + WB.base.filt.V2$Personal.time + WB.base.filt.V2$Relation.time + WB.base.filt.V2$Health.time + WB.base.filt.V2$Emot.time)/6
```

##Item-response theory

```
## Final short scale

WB.IRT <- WB.EFA.2
WB.IRT.complete <- WB.IRT[complete.cases(WB.IRT),]

WB.IRT.complete <- WB.IRT.complete[c(2,3,4,5,6,7,1,8,15,22,9:14,16:21,23:29)]

library(mirt)

model <- mirt.model('
F1 = 1,2,3,4,5,6
F2 = 7,8,9,10
F3 = 11,12,13,14,15,16,17,18,19,20,21,22,23,24,25,26,27,28,29')

mod1 <- mirt(WB.IRT.complete, model)
itemfit <- itemfit(mod1, QMC = TRUE)
summary(mod1)
```

```
##                     F1    F2    F3    h2
## Resp.time        0.732             0.536
## Leisure.time     0.871             0.759
## Personal.time    0.921             0.847
## Relation.time    0.796             0.633
## Health.time      0.719             0.516
## Emot.time        0.806             0.649
## Work.time              0.720       0.518
## Work.control           0.907       0.822
## Work.prog              0.803       0.645
## Work.content           0.892       0.795
## Resp.control                 0.741 0.549
## Leisure.control              0.732 0.535
## Personal.control             0.829 0.688
## Relation.control             0.734 0.538
## Health.control               0.638 0.407
## Emot.control                 0.765 0.584
## Resp.prog                    0.568 0.323
## Leisure.prog                 0.768 0.590
## Personal.prog                0.792 0.628
## Relation.prog                0.710 0.504
## Health.prog                  0.594 0.353
## Emot.prog                    0.843 0.711
## Resp.content                 0.785 0.617
## Leisure.content              0.797 0.636
## Personal.content             0.874 0.765
## Relation.content             0.778 0.605
## Health.content               0.648 0.420
## Emot.content                 0.812 0.660
## livingwell                   0.754 0.569
## 
## SS loadings:  3.941 2.78 10.683 
## Proportion Var:  0.136 0.096 0.368 
## 
## Factor correlations: 
## 
##    F1 F2 F3
## F1  1      
## F2  0  1   
## F3  0  0  1
```

```
write.csv(itemfit, file = "IRT.itemfit.csv")

# IRT parameters
IRT_parms <- coef(mod1, IRTpars = TRUE, simplify = TRUE)
IRT_parms$items
```

```
##                        a1       a2       a3         b1         b2          b3
## Resp.time        1.828384 0.000000 0.000000 -2.6361049 -1.2875837 -0.81413319
## Leisure.time     3.021916 0.000000 0.000000 -1.7764109 -0.7476474 -0.42836682
## Personal.time    4.010301 0.000000 0.000000 -1.7241953 -0.7989132 -0.52322133
## Relation.time    2.235451 0.000000 0.000000 -2.1609006 -1.1893135 -0.81681922
## Health.time      1.758598 0.000000 0.000000 -2.2316648 -0.7330161 -0.17485418
## Emot.time        2.314014 0.000000 0.000000 -2.1792017 -0.8338718 -0.42055254
## Work.time        0.000000 1.764920 0.000000 -1.8233176 -1.1063079 -0.76954277
## Work.control     0.000000 3.658150 0.000000 -0.9885567 -0.4018881 -0.12519955
## Work.prog        0.000000 2.294480 0.000000 -1.2733761 -0.4945210 -0.08282803
## Work.content     0.000000 3.350787 0.000000 -0.8270448 -0.2989996  0.03934716
## Resp.control     0.000000 0.000000 1.876379 -2.2849407 -1.0420845 -0.65745060
## Leisure.control  0.000000 0.000000 1.827325 -2.0411087 -0.8462183 -0.31254016
## Personal.control 0.000000 0.000000 2.527185 -1.9687983 -0.5543290 -0.19538282
## Relation.control 0.000000 0.000000 1.836860 -1.8846488 -0.8039174 -0.33728158
## Health.control   0.000000 0.000000 1.411248 -2.1818256 -0.7795192 -0.05285511
## Emot.control     0.000000 0.000000 2.018572 -1.5455236 -0.6108249 -0.14020833
## Resp.prog        0.000000 0.000000 1.175822 -2.4661973 -0.9375241 -0.04552385
## Leisure.prog     0.000000 0.000000 2.043049 -1.7299753 -0.5503526  0.11050000
## Personal.prog    0.000000 0.000000 2.209931 -1.9104459 -0.5698695  0.03247798
## Relation.prog    0.000000 0.000000 1.717168 -2.1102958 -0.9641752 -0.03766081
## Health.prog      0.000000 0.000000 1.256155 -1.9720875 -0.4910643  0.42264881
## Emot.prog        0.000000 0.000000 2.670037 -1.6337053 -0.4976860  0.09603545
## Resp.content     0.000000 0.000000 2.159275 -1.5318108 -0.4014210  0.02417543
## Leisure.content  0.000000 0.000000 2.248967 -1.4839151 -0.4894686 -0.06623527
## Personal.content 0.000000 0.000000 3.066778 -1.2116246 -0.2611469  0.09664584
## Relation.content 0.000000 0.000000 2.107344 -1.5295641 -0.5031164 -0.11980311
## Health.content   0.000000 0.000000 1.449293 -1.4563936 -0.0167461  0.57514034
## Emot.content     0.000000 0.000000 2.370727 -1.3570263 -0.3742753  0.07028010
## livingwell       0.000000 0.000000 1.955755 -1.6381297 -0.6313972 -0.15422049
##                         b4
## Resp.time        0.9622151
## Leisure.time     0.9598882
## Personal.time    0.8566931
## Relation.time    0.8117209
## Health.time      1.5366624
## Emot.time        1.2116113
## Work.time        0.7786091
## Work.control     0.9065070
## Work.prog        1.0733701
## Work.content     1.1226425
## Resp.control     1.2638311
## Leisure.control  1.2977621
## Personal.control 1.1574609
## Relation.control 1.2834235
## Health.control   1.7968379
## Emot.control     1.4919852
## Resp.prog        2.2715943
## Leisure.prog     1.6570112
## Personal.prog    1.5757892
## Relation.prog    1.4844675
## Health.prog      2.2493319
## Emot.prog        1.4867647
## Resp.content     1.7154385
## Leisure.content  1.8294239
## Personal.content 1.5440010
## Relation.content 1.4561689
## Health.content   2.3151053
## Emot.content     1.5365328
## livingwell       1.5339466
```

```
IRT_items_subset <- IRT_parms$items[1:29, ]
write.csv(IRT_items_subset, file = "IRT.parms3.csv")
```

IRT showed that all items had high discrimination within their
factors. All were > 1.0.

```
wellbeing <- WB.IRT.complete[c(11:29)]
time <- WB.IRT.complete[c(1:6)]
work <- WB.IRT.complete[c(7:10)]


# Calculate Cronbach's alpha
alpha_result_wellbeing <- psych::alpha(wellbeing, max = 100)

# Extract Cronbach's alpha
alpha_result_wellbeing <- alpha_result_wellbeing$total$raw_alpha

# Print Cronbach's alpha
print(alpha_result_wellbeing)
```

```
## [1] 0.9547908
```

```
# Calculate Cronbach's alpha
alpha_result_time <- psych::alpha(time, max = 100)

# Extract Cronbach's alpha
alpha_result_time <- alpha_result_time$total$raw_alpha

# Print Cronbach's alpha
print(alpha_result_time)
```

```
## [1] 0.865659
```

```
# Calculate Cronbach's alpha
alpha_result_work <- psych::alpha(work, max = 100)

# Extract Cronbach's alpha
alpha_result_work <- alpha_result_work$total$raw_alpha

# Print Cronbach's alpha
print(alpha_result_work)
```

```
## [1] 0.864247
```

Chronbach’s alpha for wellbeing = .95 Chronbach’s alpha for time =
.87 Chronbach’s alpha for work = .86

##Differential Item Functioning

Now, we want to do differential item functioning (DIF)

```
## Bring in demographic data that we need

Demo <- read.csv('/Users/sperrys/University of Michigan Dropbox/Sarah Sperry/EmoTe Lab/Audrey Stromberg Research/Well-being Project/Data/demo.csv')

Demos <- Demo[c(1,5,6)]

Demos$gender <- as.factor(Demos$gender)
Demos$race <- as.factor(Demos$race)

##Add it into our WB data

WBdemo <- merge(WB.base.filt.V2, Demos, by = "record_id")


## Differential Item Functioning

install.packages("MPsychoR")
install.packages("Gifi")
install.packages("smacof")
require("car")
require("psych")
require("colorspace")
require("lmerTest")
if (!require("eRm")) {install.packages("eRm"); require("eRm")}  ## Rasch models
if (!require("ltm")) {install.packages("ltm"); require("ltm")}  ## other IRT models
if (!require("mirt")) {install.packages("mirt"); require("mirt")}  ## today we use it for 1D-assessment 
if (!require("GPCMlasso")) {install.packages("GPCMlasso"); require("GPCMlasso")}   ## flexible DIF assessment

## ------------------------ DIF using GPCMlasso ---------------------------

install.packages("lordif")
library(lordif)

##Look at Sex
Candidateitems <- WBdemo[,c(4:36,42)]

# Convert tibble to data frame
Candidateitems_df <- as.data.frame(Candidateitems)

# Assuming Candidateitems_df is your data frame
Candidateitems_df[] <- lapply(Candidateitems_df, factor)

Candidateitems_df$gender <- as.factor(Candidateitems_df$gender)
summary(Candidateitems_df$gender)

## In order to do DIF each group must have at least 5 cases. gender non-binary only has 2 cases so we need to get rid of this

Candidateitems_df2 <- Candidateitems_df %>%
  filter(gender != "Non-binary/genderqueer")

filtered_df <- Candidateitems_df[Candidateitems_df$gender != "Non-binary/genderqueer", ]
# Drop unused levels from the gender factor
filtered_df$gender <- droplevels(filtered_df$gender)

summary(Candidateitems_df2)


gender.DIF <- lordif(filtered_df[c(1:33)],filtered_df$gender)
summary(gender.DIF)
print(gender.DIF)
```

## 5 Test-retest

```
## rename variables to be more usable
library(dplyr)
WB.df.2 <- WB.df %>%
  rename(Work.time = wb_1_1, 
         Resp.time = wb_2_1, 
         Leisure.time = wb_3_1, 
         Personal.time = wb_4_1, 
         Relation.time = wb_5_1, 
         Health.time = wb_6_1, 
         Emot.time = wb_7_1, 
         Other.time = wb_8_1, 
         Work.control = wb_1_2, 
         Resp.control = wb_2_2, 
         Leisure.control = wb_3_2, 
         Personal.control = wb_4_2, 
         Relation.control = wb_5_2, 
         Health.control = wb_6_2, 
         Emot.control = wb_7_2, 
         Other.control = wb_8_2,
         Work.prog = wb_1_3, 
         Resp.prog = wb_2_3, 
         Leisure.prog = wb_3_3, 
         Personal.prog = wb_4_3, 
         Relation.prog = wb_5_3, 
         Health.prog = wb_6_3, 
         Emot.prog = wb_7_3, 
         Other.prog = wb_8_3, 
         Work.content = wb_1_4, 
         Resp.content = wb_2_4, 
         Leisure.content = wb_3_4, 
         Personal.content = wb_4_4, 
         Relation.content = wb_5_4, 
         Health.content = wb_6_4, 
         Emot.content = wb_7_4, 
         Other.content = wb_8_4,
         livingwell = wb_5)

WB.df.2$date <- as.POSIXct(WB.df.2$wb_part_b_timestamp, format = "%m/%d/%y")

## Filter for complete quant surveys 

WB.df.3 <- WB.df.2 %>%
  filter(wellness_survey_part_b_complete== 2)

## Choose variable we want

WB.df.4 <- WB.df.3[c(1,2,36:69)]

## Recode redcap_event_name
WB.df.4 <- WB.df.4 %>%
  mutate(Time = case_when(
    redcap_event_name == "baseline_arm_1" ~ 0,
    redcap_event_name == "month_2_arm_1" ~ 1
  ))

## Calculate how much actual time there was between assessments 

library(lubridate)

WB.df.4$startdate <- as.POSIXct(WB.df.4$wb_part_b_timestamp, format = "%m/%d/%y", tz='UTC')


calculate_day_in_study <- function(data) {
  data <- data %>%
    arrange(record_id, startdate) %>%
    group_by(record_id) %>%
    mutate(timediff = as.integer(difftime(startdate, first(startdate), units = "days"))) %>%
    ungroup() }

WB.df.4 <- calculate_day_in_study(WB.df.4)


## Calculate subscale scores 

## Create subscales scores in reduced data: 

WB.df.4$wellbeing <- (WB.df.4$Resp.control + WB.df.4$Leisure.control + WB.df.4$Personal.control + WB.df.4$Relation.control + WB.df.4$Health.control + WB.df.4$Emot.control + WB.df.4$Resp.prog + WB.df.4$Leisure.prog + WB.df.4$Personal.prog + WB.df.4$Relation.prog + WB.df.4$Health.prog + WB.df.4$Emot.prog + 
WB.df.4$Resp.content + WB.df.4$Leisure.content + WB.df.4$Personal.content + WB.df.4$Relation.content + WB.df.4$Health.content + WB.df.4$Emot.content + WB.df.4$livingwell)/19

WB.df.4$workfunctioning <- (WB.df.4$Work.time + WB.df.4$Work.control + WB.df.4$Work.prog + WB.df.4$Work.content)/4

WB.df.4$enoughtime <- (WB.df.4$Resp.time + WB.df.4$Leisure.time + WB.df.4$Personal.time + WB.df.4$Relation.time + WB.df.4$Health.time + WB.df.4$Emot.time)/6


## plot individual slope lines of each of their scores from time 0 to time 1 

library(ggplot2)

wellbeingplot <- ggplot(WB.df.4, aes(x = Time, y = wellbeing, group = record_id)) +
  geom_line(aes(color = factor(record_id)), show.legend = FALSE) + 
  geom_point(aes(color = factor(record_id)), show.legend = FALSE) +
  labs(x = "Time Point", y = "Score", color = "Individual") +
  theme_minimal() + theme(legend.position = "FALSE")

workplot <- ggplot(WB.df.4, aes(x = Time, y = workfunctioning, group = record_id)) +
  geom_line(aes(color = factor(record_id)), show.legend = FALSE) + 
  geom_point(aes(color = factor(record_id)), show.legend = FALSE) +
  labs(x = "Time Point", y = "Score", color = "Individual") +
  theme_minimal() + theme(legend.position = "FALSE")

timeplot <- ggplot(WB.df.4, aes(x = Time, y = enoughtime, group = record_id)) +
  geom_line(aes(color = factor(record_id)), show.legend = FALSE) + 
  geom_point(aes(color = factor(record_id)), show.legend = FALSE) +
  labs(x = "Time Point", y = "Score", color = "Individual") +
  theme_minimal() + theme(legend.position = "FALSE")


## Quantify within and between person variance in our scale from one time point to the next 

library(lme4)
timemodel <- lmer(wellbeing ~ timediff + (1 | record_id), WB.df.4)
summary(timemodel)
```

```
## Linear mixed model fit by REML. t-tests use Satterthwaite's method [
## lmerModLmerTest]
## Formula: wellbeing ~ timediff + (1 | record_id)
##    Data: WB.df.4
## 
## REML criterion at convergence: 915
## 
## Scaled residuals: 
##      Min       1Q   Median       3Q      Max 
## -3.15661 -0.46550  0.07861  0.46491  2.99563 
## 
## Random effects:
##  Groups    Name        Variance Std.Dev.
##  record_id (Intercept) 0.4874   0.6982  
##  Residual              0.3509   0.5924  
## Number of obs: 365, groups:  record_id, 193
## 
## Fixed effects:
##               Estimate Std. Error         df t value Pr(>|t|)    
## (Intercept)  3.358e+00  6.537e-02  2.672e+02  51.370   <2e-16 ***
## timediff    -1.342e-03  7.005e-04  1.826e+02  -1.916    0.057 .  
## ---
## Signif. codes:  0 '***' 0.001 '**' 0.01 '*' 0.05 '.' 0.1 ' ' 1
## 
## Correlation of Fixed Effects:
##          (Intr)
## timediff -0.421
```

```
# Extract variance components
vc <- VarCorr(timemodel)
vc
```

```
##  Groups    Name        Std.Dev.
##  record_id (Intercept) 0.69816 
##  Residual              0.59239
```

```
# Extract the variance components
sigma2_between <- attr(vc, "sc")^2
sigma2_within <- as.numeric(vc$record_id)

# Calculate the ICCs
icc_within <- sigma2_within / (sigma2_within + sigma2_between)
icc_between <- sigma2_between / (sigma2_within + sigma2_between)

# Print the ICCs
icc_within  ## value = 0.58
```

```
## [1] 0.5814105
```

```
icc_between ## value = 0.42
```

```
## [1] 0.4185895
```

```
workmodel <- lmer(workfunctioning ~ timediff + (1 | record_id), WB.df.4)
summary(workmodel)
```

```
## Linear mixed model fit by REML. t-tests use Satterthwaite's method [
## lmerModLmerTest]
## Formula: workfunctioning ~ timediff + (1 | record_id)
##    Data: WB.df.4
## 
## REML criterion at convergence: 1093.3
## 
## Scaled residuals: 
##      Min       1Q   Median       3Q      Max 
## -2.49284 -0.48854  0.07126  0.52155  2.36674 
## 
## Random effects:
##  Groups    Name        Variance Std.Dev.
##  record_id (Intercept) 0.7830   0.8849  
##  Residual              0.5795   0.7613  
## Number of obs: 365, groups:  record_id, 193
## 
## Fixed effects:
##               Estimate Std. Error         df t value Pr(>|t|)    
## (Intercept)  3.328e+00  8.332e-02  2.705e+02   39.94   <2e-16 ***
## timediff    -4.766e-04  8.999e-04  1.854e+02   -0.53    0.597    
## ---
## Signif. codes:  0 '***' 0.001 '**' 0.01 '*' 0.05 '.' 0.1 ' ' 1
## 
## Correlation of Fixed Effects:
##          (Intr)
## timediff -0.425
```

```
# Extract variance components
vc <- VarCorr(workmodel)
vc
```

```
##  Groups    Name        Std.Dev.
##  record_id (Intercept) 0.88485 
##  Residual              0.76128
```

```
# Extract the variance components
sigma2_between <- attr(vc, "sc")^2
sigma2_within <- as.numeric(vc$record_id)

# Calculate the ICCs
icc_within <- sigma2_within / (sigma2_within + sigma2_between)
icc_between <- sigma2_between / (sigma2_within + sigma2_between)

# Print the ICCs
icc_within  ## value = 0.57
```

```
## [1] 0.574651
```

```
icc_between ## value = 0.43
```

```
## [1] 0.425349
```

```
enoughtimemodel <- lmer(enoughtime ~ timediff + (1 | record_id), WB.df.4)
summary(enoughtimemodel)
```

```
## Linear mixed model fit by REML. t-tests use Satterthwaite's method [
## lmerModLmerTest]
## Formula: enoughtime ~ timediff + (1 | record_id)
##    Data: WB.df.4
## 
## REML criterion at convergence: 940.1
## 
## Scaled residuals: 
##     Min      1Q  Median      3Q     Max 
## -3.1882 -0.4702  0.0698  0.5570  2.6231 
## 
## Random effects:
##  Groups    Name        Variance Std.Dev.
##  record_id (Intercept) 0.4060   0.6372  
##  Residual              0.4328   0.6579  
## Number of obs: 365, groups:  record_id, 193
## 
## Fixed effects:
##               Estimate Std. Error         df t value Pr(>|t|)    
## (Intercept)  3.623e+00  6.521e-02  2.887e+02  55.557   <2e-16 ***
## timediff    -1.639e-03  7.738e-04  1.893e+02  -2.118   0.0355 *  
## ---
## Signif. codes:  0 '***' 0.001 '**' 0.01 '*' 0.05 '.' 0.1 ' ' 1
## 
## Correlation of Fixed Effects:
##          (Intr)
## timediff -0.469
```

```
# Extract variance components
vc <- VarCorr(enoughtimemodel)
vc
```

```
##  Groups    Name        Std.Dev.
##  record_id (Intercept) 0.63717 
##  Residual              0.65785
```

```
# Extract the variance components
sigma2_between <- attr(vc, "sc")^2
sigma2_within <- as.numeric(vc$record_id)

# Calculate the ICCs
icc_within <- sigma2_within / (sigma2_within + sigma2_between)
icc_between <- sigma2_between / (sigma2_within + sigma2_between)

# Print the ICCs
icc_within  ## value = 0.48
```

```
## [1] 0.4840321
```

```
icc_between ## value = 0.52
```

```
## [1] 0.5159679
```

```
## 

# Reshape data to wide format
library(tidyr)

wellbeingdf <- WB.df.4[c("record_id", "Time", "wellbeing")]

data_wide <- wellbeingdf %>%
  pivot_wider(names_from = Time, values_from = wellbeing, names_prefix = "time_")

# Remove rows with NAs
data_wide <- na.omit(data_wide)

# Calculate the test-retest correlation
test_retest_correlation <- cor(data_wide$time_0, data_wide$time_1)

# Print the correlation
test_retest_correlation ## VALUE IS 0.57
```

```
## [1] 0.5735565
```

```
workdf <- WB.df.4[c("record_id", "Time", "workfunctioning")]

data_wide <- workdf %>%
  pivot_wider(names_from = Time, values_from = workfunctioning, names_prefix = "time_")

# Remove rows with NAs
data_wide <- na.omit(data_wide)

# Calculate the test-retest correlation
test_retest_correlation <- cor(data_wide$time_0, data_wide$time_1)

# Print the correlation
test_retest_correlation ## VALUE IS 0.58
```

```
## [1] 0.577556
```

```
enoughtime <- WB.df.4[c("record_id", "Time", "enoughtime")]

data_wide <- enoughtime %>%
  pivot_wider(names_from = Time, values_from = enoughtime, names_prefix = "time_")

# Remove rows with NAs
data_wide <- na.omit(data_wide)

# Calculate the test-retest correlation
test_retest_correlation <- cor(data_wide$time_0, data_wide$time_1)

# Print the correlation
test_retest_correlation ## VALUE IS 0.49
```

```
## [1] 0.490331
```

## 6 Convergent and Divergent Validity

Bring in all of our other data

```
asrm <- read.csv('/Users/sperrys/University of Michigan Dropbox/Sarah Sperry/EmoTe Lab/Audrey Stromberg Research/Well-being Project/Data/asrm.csv')

audit <- read.csv('/Users/sperrys/University of Michigan Dropbox/Sarah Sperry/EmoTe Lab/Audrey Stromberg Research/Well-being Project/Data/audit_c.csv')

dudit <- read.csv('/Users/sperrys/University of Michigan Dropbox/Sarah Sperry/EmoTe Lab/Audrey Stromberg Research/Well-being Project/Data/dudit_c.csv')

cudit <- read.csv('/Users/sperrys/University of Michigan Dropbox/Sarah Sperry/EmoTe Lab/Audrey Stromberg Research/Well-being Project/Data/cudit_c.csv')

phq <- read.csv('/Users/sperrys/University of Michigan Dropbox/Sarah Sperry/EmoTe Lab/Audrey Stromberg Research/Well-being Project/Data/phq9.csv')

gad <- read.csv('/Users/sperrys/University of Michigan Dropbox/Sarah Sperry/EmoTe Lab/Audrey Stromberg Research/Well-being Project/Data/gad7.csv')

sf12 <- read.csv('/Users/sperrys/University of Michigan Dropbox/Sarah Sperry/EmoTe Lab/Audrey Stromberg Research/Well-being Project/Data/sf12.csv')


scales1 <- merge(asrm,phq, by = "record_id")
scales1 <- merge(scales1,gad, by = "record_id")

smallerscales <- merge(audit, dudit, by = "record_id")
smallerscales <- merge(smallerscales, cudit, by = "record_id")
smallerscales <- merge(smallerscales, sf12, by = "record_id")


WBscales <- merge(WB.base.filt.V2, scales1, by = "record_id")
WBscales2 <- merge(WB.base.filt.V2, smallerscales, by = "record_id")

## Descriptive statistics
Descriptives <- WBscales[c(1, 50, 76, 89)]
Descriptives2 <- WBscales2[c(1, 125, 126, 66, 58, 49)]
Descriptivesall <- merge(Descriptives, Descriptives2, by = "record_id")

table1::table1(~phq_score + gad_score + asrm_score + audit_c_total + dudit_c_total + cudit_c_total + pcs12_tscore + mcs12_tscore,  Descriptivesall)
```

|  | Overall (N=87) |
| --- | --- |
| phq\_score |  |
| Mean (SD) | 7.33 (6.46) |
| Median [Min, Max] | 5.00 [0, 27.0] |
| gad\_score |  |
| Mean (SD) | 6.64 (5.95) |
| Median [Min, Max] | 6.00 [0, 21.0] |
| asrm\_score |  |
| Mean (SD) | 1.91 (2.92) |
| Median [Min, Max] | 1.00 [0, 13.0] |
| audit\_c\_total |  |
| Mean (SD) | 1.44 (2.06) |
| Median [Min, Max] | 1.00 [0, 10.0] |
| dudit\_c\_total |  |
| Mean (SD) | 0.655 (1.99) |
| Median [Min, Max] | 0 [0, 11.0] |
| cudit\_c\_total |  |
| Mean (SD) | 1.01 (2.28) |
| Median [Min, Max] | 0 [0, 10.0] |
| pcs12\_tscore |  |
| Mean (SD) | 45.5 (12.1) |
| Median [Min, Max] | 48.8 [18.3, 64.6] |
| mcs12\_tscore |  |
| Mean (SD) | 41.7 (11.8) |
| Median [Min, Max] | 42.5 [10.8, 60.2] |

```
subscales <- WBscales[c(89,76, 50,40,41,39)]
cor <- cor(subscales)

# Create corrplot
library(corrplot)
corrplot(cor, method = "circle", type = "upper", order = "hclust",
         tl.col = "black", tl.srt = 45, diag = FALSE)
```

```
## Regression Models
## library car for VIF based on Reviewer request 
library (car)

moodmodel <- lm(phq_score ~ wellbeing + workfunctioning + enoughtime, data = WBscales)
summary (moodmodel)
```

```
## 
## Call:
## lm(formula = phq_score ~ wellbeing + workfunctioning + enoughtime, 
##     data = WBscales)
## 
## Residuals:
##      Min       1Q   Median       3Q      Max 
## -12.4206  -3.0175  -0.6768   2.4799  13.3330 
## 
## Coefficients:
##                 Estimate Std. Error t value Pr(>|t|)    
## (Intercept)      21.0006     2.1870   9.602 5.67e-16 ***
## wellbeing        -5.4139     0.7150  -7.572 1.63e-11 ***
## workfunctioning  -0.4232     0.4891  -0.865  0.38889    
## enoughtime        1.6437     0.6244   2.632  0.00978 ** 
## ---
## Signif. codes:  0 '***' 0.001 '**' 0.01 '*' 0.05 '.' 0.1 ' ' 1
## 
## Residual standard error: 4.638 on 103 degrees of freedom
## Multiple R-squared:  0.4777, Adjusted R-squared:  0.4625 
## F-statistic:  31.4 on 3 and 103 DF,  p-value: 1.689e-14
```

```
vif(moodmodel)
```

```
##       wellbeing workfunctioning      enoughtime 
##        1.917771        1.446718        1.451325
```

```
moodmodel2 <- lm(asrm_score ~ wellbeing + workfunctioning + enoughtime, data = WBscales)
summary (moodmodel2)
```

```
## 
## Call:
## lm(formula = asrm_score ~ wellbeing + workfunctioning + enoughtime, 
##     data = WBscales)
## 
## Residuals:
##     Min      1Q  Median      3Q     Max 
## -2.2500 -1.7552 -1.3486  0.3933 11.3219 
## 
## Coefficients:
##                 Estimate Std. Error t value Pr(>|t|)
## (Intercept)      1.08859    1.31887   0.825    0.411
## wellbeing       -0.09169    0.43117  -0.213    0.832
## workfunctioning  0.03484    0.29496   0.118    0.906
## enoughtime       0.23702    0.37657   0.629    0.530
## 
## Residual standard error: 2.797 on 103 degrees of freedom
## Multiple R-squared:  0.004348,   Adjusted R-squared:  -0.02465 
## F-statistic: 0.1499 on 3 and 103 DF,  p-value: 0.9295
```

```
vif(moodmodel2)
```

```
##       wellbeing workfunctioning      enoughtime 
##        1.917771        1.446718        1.451325
```

```
moodmodel3 <- lm(gad_score ~ wellbeing + workfunctioning + enoughtime, data = WBscales)
summary (moodmodel3)
```

```
## 
## Call:
## lm(formula = gad_score ~ wellbeing + workfunctioning + enoughtime, 
##     data = WBscales)
## 
## Residuals:
##    Min     1Q Median     3Q    Max 
## -8.181 -3.733 -1.089  2.488 16.003 
## 
## Coefficients:
##                 Estimate Std. Error t value Pr(>|t|)    
## (Intercept)      17.2274     2.3771   7.247 8.06e-11 ***
## wellbeing        -3.8780     0.7771  -4.990 2.46e-06 ***
## workfunctioning  -0.6278     0.5316  -1.181   0.2404    
## enoughtime        1.2983     0.6787   1.913   0.0585 .  
## ---
## Signif. codes:  0 '***' 0.001 '**' 0.01 '*' 0.05 '.' 0.1 ' ' 1
## 
## Residual standard error: 5.041 on 103 degrees of freedom
## Multiple R-squared:  0.3102, Adjusted R-squared:  0.2901 
## F-statistic: 15.44 on 3 and 103 DF,  p-value: 2.298e-08
```

```
vif(moodmodel3)
```

```
##       wellbeing workfunctioning      enoughtime 
##        1.917771        1.446718        1.451325
```

```
tab_model(moodmodel, moodmodel2, moodmodel3, show.std = TRUE)
```

|  | phq\_score | | | | | asrm\_score | | | | | gad\_score | | | | |
| --- | --- | --- | --- | --- | --- | --- | --- | --- | --- | --- | --- | --- | --- | --- | --- |
| Predictors | Estimates | std. Beta | CI | standardized CI | p | Estimates | std. Beta | CI | standardized CI | p | Estimates | std. Beta | CI | standardized CI | p |
| (Intercept) | 21.00 | 0.00 | 16.66 – 25.34 | -0.14 – 0.14 | **<0.001** | 1.09 | 0.00 | -1.53 – 3.70 | -0.19 – 0.19 | 0.411 | 17.23 | 0.00 | 12.51 – 21.94 | -0.16 – 0.16 | **<0.001** |
| wellbeing | -5.41 | -0.75 | -6.83 – -4.00 | -0.94 – -0.55 | **<0.001** | -0.09 | -0.03 | -0.95 – 0.76 | -0.30 – 0.24 | 0.832 | -3.88 | -0.57 | -5.42 – -2.34 | -0.79 – -0.34 | **<0.001** |
| workfunctioning | -0.42 | -0.07 | -1.39 – 0.55 | -0.24 – 0.10 | 0.389 | 0.03 | 0.01 | -0.55 – 0.62 | -0.22 – 0.25 | 0.906 | -0.63 | -0.12 | -1.68 – 0.43 | -0.31 – 0.08 | 0.240 |
| enoughtime | 1.64 | 0.23 | 0.41 – 2.88 | 0.06 – 0.40 | **0.010** | 0.24 | 0.07 | -0.51 – 0.98 | -0.16 – 0.31 | 0.530 | 1.30 | 0.19 | -0.05 – 2.64 | -0.01 – 0.38 | 0.059 |
| Observations | 107 | | | | | 107 | | | | | 107 | | | | |
| R^2^ / R^2^ adjusted | 0.478 / 0.463 | | | | | 0.004 / -0.025 | | | | | 0.310 / 0.290 | | | | |

```
auditmod <- lm(audit_c_total ~ wellbeing + workfunctioning + enoughtime, data = WBscales2)
summary (auditmod)
```

```
## 
## Call:
## lm(formula = audit_c_total ~ wellbeing + workfunctioning + enoughtime, 
##     data = WBscales2)
## 
## Residuals:
##     Min      1Q  Median      3Q     Max 
## -3.6403 -1.2197 -0.3578  0.9464  5.2794 
## 
## Coefficients:
##                 Estimate Std. Error t value Pr(>|t|)    
## (Intercept)       2.1719     0.9812   2.213  0.02955 *  
## wellbeing        -1.4959     0.3450  -4.336 3.97e-05 ***
## workfunctioning   0.3417     0.2163   1.580  0.11783    
## enoughtime        0.8813     0.2968   2.969  0.00388 ** 
## ---
## Signif. codes:  0 '***' 0.001 '**' 0.01 '*' 0.05 '.' 0.1 ' ' 1
## 
## Residual standard error: 1.878 on 85 degrees of freedom
## Multiple R-squared:  0.1854, Adjusted R-squared:  0.1567 
## F-statistic:  6.45 on 3 and 85 DF,  p-value: 0.0005487
```

```
vif(auditmod)
```

```
##       wellbeing workfunctioning      enoughtime 
##        2.124334        1.408043        1.628898
```

```
duditmod <- lm(dudit_c_total ~ wellbeing + workfunctioning + enoughtime, data = WBscales2)
summary (duditmod)
```

```
## 
## Call:
## lm(formula = dudit_c_total ~ wellbeing + workfunctioning + enoughtime, 
##     data = WBscales2)
## 
## Residuals:
##     Min      1Q  Median      3Q     Max 
## -1.8639 -0.8784 -0.5491 -0.2114  9.8251 
## 
## Coefficients:
##                 Estimate Std. Error t value Pr(>|t|)  
## (Intercept)      -0.4559     1.0344  -0.441   0.6605  
## wellbeing        -0.1825     0.3637  -0.502   0.6172  
## workfunctioning  -0.1356     0.2280  -0.595   0.5537  
## enoughtime        0.6065     0.3129   1.938   0.0559 .
## ---
## Signif. codes:  0 '***' 0.001 '**' 0.01 '*' 0.05 '.' 0.1 ' ' 1
## 
## Residual standard error: 1.98 on 85 degrees of freedom
## Multiple R-squared:  0.05149,    Adjusted R-squared:  0.01801 
## F-statistic: 1.538 on 3 and 85 DF,  p-value: 0.2106
```

```
vif(duditmod)
```

```
##       wellbeing workfunctioning      enoughtime 
##        2.124334        1.408043        1.628898
```

```
cuditmod <- lm(cudit_c_total ~ wellbeing + workfunctioning + enoughtime, data = WBscales2)
summary (cuditmod)
```

```
## 
## Call:
## lm(formula = cudit_c_total ~ wellbeing + workfunctioning + enoughtime, 
##     data = WBscales2)
## 
## Residuals:
##     Min      1Q  Median      3Q     Max 
## -2.6203 -1.1548 -0.8352  0.0655  8.2304 
## 
## Coefficients:
##                 Estimate Std. Error t value Pr(>|t|)  
## (Intercept)     -0.57096    1.17045  -0.488   0.6269  
## wellbeing       -0.40029    0.41153  -0.973   0.3335  
## workfunctioning -0.03895    0.25795  -0.151   0.8803  
## enoughtime       0.84765    0.35406   2.394   0.0189 *
## ---
## Signif. codes:  0 '***' 0.001 '**' 0.01 '*' 0.05 '.' 0.1 ' ' 1
## 
## Residual standard error: 2.24 on 85 degrees of freedom
## Multiple R-squared:  0.06648,    Adjusted R-squared:  0.03353 
## F-statistic: 2.018 on 3 and 85 DF,  p-value: 0.1175
```

```
vif(cuditmod)
```

```
##       wellbeing workfunctioning      enoughtime 
##        2.124334        1.408043        1.628898
```

```
MCSmodel <- lm(mcs12_tscore ~ wellbeing + workfunctioning + enoughtime, data = WBscales2)
summary (MCSmodel)
```

```
## 
## Call:
## lm(formula = mcs12_tscore ~ wellbeing + workfunctioning + enoughtime, 
##     data = WBscales2)
## 
## Residuals:
##     Min      1Q  Median      3Q     Max 
## -24.926  -3.838   1.241   5.702  17.700 
## 
## Coefficients:
##                 Estimate Std. Error t value Pr(>|t|)    
## (Intercept)       13.330      4.672   2.853  0.00543 ** 
## wellbeing          9.258      1.643   5.636 2.22e-07 ***
## workfunctioning    1.077      1.030   1.046  0.29843    
## enoughtime        -1.810      1.413  -1.281  0.20367    
## ---
## Signif. codes:  0 '***' 0.001 '**' 0.01 '*' 0.05 '.' 0.1 ' ' 1
## 
## Residual standard error: 8.941 on 85 degrees of freedom
## Multiple R-squared:  0.4332, Adjusted R-squared:  0.4132 
## F-statistic: 21.66 on 3 and 85 DF,  p-value: 1.64e-10
```

```
vif(MCSmodel)
```

```
##       wellbeing workfunctioning      enoughtime 
##        2.124334        1.408043        1.628898
```

```
PCSmodel <- lm(pcs12_tscore ~ wellbeing + workfunctioning + enoughtime, data = WBscales2)
summary (PCSmodel)
```

```
## 
## Call:
## lm(formula = pcs12_tscore ~ wellbeing + workfunctioning + enoughtime, 
##     data = WBscales2)
## 
## Residuals:
##     Min      1Q  Median      3Q     Max 
## -27.964  -7.996   2.801   9.635  18.628 
## 
## Coefficients:
##                 Estimate Std. Error t value Pr(>|t|)    
## (Intercept)       40.472      6.310   6.413 7.67e-09 ***
## wellbeing          2.127      2.219   0.958    0.341    
## workfunctioning    1.213      1.391   0.872    0.385    
## enoughtime        -1.636      1.909  -0.857    0.394    
## ---
## Signif. codes:  0 '***' 0.001 '**' 0.01 '*' 0.05 '.' 0.1 ' ' 1
## 
## Residual standard error: 12.08 on 85 degrees of freedom
## Multiple R-squared:  0.03751,    Adjusted R-squared:  0.003541 
## F-statistic: 1.104 on 3 and 85 DF,  p-value: 0.352
```

```
vif(PCSmodel)
```

```
##       wellbeing workfunctioning      enoughtime 
##        2.124334        1.408043        1.628898
```

```
tab_model(auditmod,duditmod,cuditmod, show.std = TRUE)
```

|  | audit\_c\_total | | | | | dudit\_c\_total | | | | | cudit\_c\_total | | | | |
| --- | --- | --- | --- | --- | --- | --- | --- | --- | --- | --- | --- | --- | --- | --- | --- |
| Predictors | Estimates | std. Beta | CI | standardized CI | p | Estimates | std. Beta | CI | standardized CI | p | Estimates | std. Beta | CI | standardized CI | p |
| (Intercept) | 2.17 | -0.00 | 0.22 – 4.12 | -0.19 – 0.19 | **0.030** | -0.46 | 0.00 | -2.51 – 1.60 | -0.21 – 0.21 | 0.661 | -0.57 | 0.00 | -2.90 – 1.76 | -0.21 – 0.21 | 0.627 |
| wellbeing | -1.50 | -0.62 | -2.18 – -0.81 | -0.90 – -0.33 | **<0.001** | -0.18 | -0.08 | -0.91 – 0.54 | -0.38 – 0.23 | 0.617 | -0.40 | -0.15 | -1.22 – 0.42 | -0.45 – 0.16 | 0.333 |
| workfunctioning | 0.34 | 0.18 | -0.09 – 0.77 | -0.05 – 0.41 | 0.118 | -0.14 | -0.07 | -0.59 – 0.32 | -0.32 – 0.17 | 0.554 | -0.04 | -0.02 | -0.55 – 0.47 | -0.27 – 0.23 | 0.880 |
| enoughtime | 0.88 | 0.37 | 0.29 – 1.47 | 0.12 – 0.62 | **0.004** | 0.61 | 0.26 | -0.02 – 1.23 | -0.01 – 0.53 | 0.056 | 0.85 | 0.32 | 0.14 – 1.55 | 0.05 – 0.59 | **0.019** |
| Observations | 89 | | | | | 89 | | | | | 89 | | | | |
| R^2^ / R^2^ adjusted | 0.185 / 0.157 | | | | | 0.051 / 0.018 | | | | | 0.066 / 0.034 | | | | |

```
tab_model(MCSmodel,PCSmodel, show.std = TRUE)
```

|  | mcs12\_tscore | | | | | pcs12\_tscore | | | | |
| --- | --- | --- | --- | --- | --- | --- | --- | --- | --- | --- |
| Predictors | Estimates | std. Beta | CI | standardized CI | p | Estimates | std. Beta | CI | standardized CI | p |
| (Intercept) | 13.33 | -0.00 | 4.04 – 22.62 | -0.16 – 0.16 | **0.005** | 40.47 | 0.00 | 27.92 – 53.02 | -0.21 – 0.21 | **<0.001** |
| wellbeing | 9.26 | 0.67 | 5.99 – 12.52 | 0.43 – 0.91 | **<0.001** | 2.13 | 0.15 | -2.28 – 6.54 | -0.16 – 0.46 | 0.341 |
| workfunctioning | 1.08 | 0.10 | -0.97 – 3.12 | -0.09 – 0.29 | 0.298 | 1.21 | 0.11 | -1.55 – 3.98 | -0.14 – 0.36 | 0.385 |
| enoughtime | -1.81 | -0.13 | -4.62 – 1.00 | -0.34 – 0.07 | 0.204 | -1.64 | -0.12 | -5.43 – 2.16 | -0.39 – 0.15 | 0.394 |
| Observations | 89 | | | | | 89 | | | | |
| R^2^ / R^2^ adjusted | 0.433 / 0.413 | | | | | 0.038 / 0.004 | | | | |

```
figuredf <- WBscales[c(41,76)]

# Plotting using ggplot2
ggplot(figuredf, aes(x = phq_score, y = as.numeric(as.character(enoughtime)))) +
  geom_point(position = position_jitter(width = 0.2, height = 0), alpha = 0.6) +
  geom_smooth(method = "lm", se = FALSE) +
  labs(title = "Scatter plots with Linear Regression Lines",
       x = "Depression",
       y = "Have enough time to spend on...") +
  theme_minimal() +
  theme(legend.position = "none")
```

```
Correlations <- WBscales2[c(39,40,41,49,58,66,125,126)]
cors <- cor(Correlations)

# Create corrplot
library(corrplot)
corrplot(cors, method = "circle", type = "upper", order = "hclust",
         tl.col = "black", tl.srt = 45, diag = FALSE)
```

```
Correlations <- WBscales[c(39,40,41,76,50,89)]
cor(Correlations)
```

```
##                   wellbeing workfunctioning  enoughtime   phq_score  asrm_score
## wellbeing        1.00000000      0.55534520  0.55731818 -0.66198956  0.02035382
## workfunctioning  0.55534520      1.00000000  0.29348868 -0.42250393  0.01976973
## enoughtime       0.55731818      0.29348868  1.00000000 -0.21207511  0.06251634
## phq_score       -0.66198956     -0.42250393 -0.21207511  1.00000000 -0.05069864
## asrm_score       0.02035382      0.01976973  0.06251634 -0.05069864  1.00000000
## gad_score       -0.52498357     -0.37495616 -0.16071057  0.68315019 -0.03267049
##                   gad_score
## wellbeing       -0.52498357
## workfunctioning -0.37495616
## enoughtime      -0.16071057
## phq_score        0.68315019
## asrm_score      -0.03267049
## gad_score        1.00000000
```

## 7 Satisfaction Survey

```
WB.SS <- WB.df.3 [c(1, 2, 73:87)]

## Filter for complete quant surveys 

WB.SS <- WB.SS %>%
  filter(wellness_measure_satisfaction_survey_complete== 2)


## rename for interpretability

WB.SS.2 <- WB.SS %>%
  rename(takeagain = wb_ss_1, 
         howoften = wb_ss_1a, 
         exampledifficulty = wb_ss_2, 
         timedifficulty = wb_ss_3a, 
         progressdifficulty = wb_ss_3b, 
         controldifficulty = wb_ss_3c, 
         contentdifficulty = wb_ss_3d, 
         impactedwellness = wb_ss_4a, 
         impactedbehavior = wb_ss_4b, 
         impactedgoals = wb_ss_4c, 
         qual.like = wb_ss_5, 
         qual.other = wb_ss_6)
        
WB.SS.2$takeagain <- as.factor(WB.SS.2$takeagain)
summary(WB.SS.2$takeagain)
```

```
##   0   1   2 
##   4 153  11
```

```
WB.SS.2$howoften <- as.factor(WB.SS.2$howoften)

summary(WB.SS.2$howoften)
```

```
##   0   1   2 
## 119  44   5
```

```
## CREATE PLOTS FOR QUESTION 3

Question3 <- WB.SS.2[c("exampledifficulty", "timedifficulty", "progressdifficulty", "controldifficulty", "contentdifficulty")]

# Reshape data from wide to long format
df_long <- gather(Question3, key = "Variable", value = "Response")

df_long <- df_long %>%
  mutate(Response = case_when(
    Response == 0 ~ "Very Difficult",
    Response == 1 ~ "Difficult",
    Response == 2 ~ "Neutral",
    Response == 3 ~ "Easy",
    Response == 4 ~ "Very Easy"
  ))


# Reorder levels of Response variable
df_long$Response <- factor(df_long$Response, 
                           levels = c("Very Easy", "Easy", "Neutral", "Difficult", "Very Difficult"))

# Plotting using ggplot2
plot.q1 <- ggplot(df_long, aes(x = Variable, y = ..count.., fill = as.factor(Response))) +
  
  # Horizontal layered bar graph
  geom_bar(position = "stack", alpha = 0.8) +
  
  # Adjusting bar width and colors
  scale_fill_viridis_d(name = "Response") +
  
  # Styling and labels
  theme_minimal() +
  labs(
    x = "Domain",
    y = "Count",
    fill = "Response",
    title = "How easy or difficult did you find it to:"
  ) +
  
  # Flip the coordinates to make it horizontal
  coord_flip()

# Display the plot
print(plot.q1)
```

```
## CREATE PLOTS FOR QUESTION 4

Question4 <- WB.SS.2[c("impactedwellness", "impactedbehavior", "impactedgoals")]

# Reshape data from wide to long format
df_long <- gather(Question4, key = "Variable", value = "Response")

df_long <- df_long %>%
  mutate(Response = case_when(
    Response == 0 ~ "Strongly Disagree",
    Response == 1 ~ "Disagree",
    Response == 2 ~ "Neutral",
    Response == 3 ~ "Agree",
    Response == 4 ~ "Strongly Agree"
  ))


# Reorder levels of Response variable
df_long$Response <- factor(df_long$Response, 
                           levels = c("Strongly Agree", "Agree", "Neutral", "Disagree", "Strongly Disagree"))

# Plotting using ggplot2
plot.q3 <- ggplot(df_long, aes(x = Variable, y = ..count.., fill = as.factor(Response))) +
  
  # Horizontal layered bar graph
  geom_bar(position = "stack", alpha = 0.8) +
  
  # Adjusting bar width and colors
  scale_fill_viridis_d(name = "Response") +
  
  # Styling and labels
  theme_minimal() +
  labs(
    x = "Domain",
    y = "Count",
    fill = "Response",
    title = "Completing the survey:"
  ) +
  
  # Flip the coordinates to make it horizontal
  coord_flip()

# Display the plot
print(plot.q3)
```

Results: 4 said they would not take it again 153 said they would take it
again 11 were undecided

119 said they would take it every 2 months 44 said they would take it
every 6 months 5 said once per year
